# PrEP Implementation Science: State-of-the-Art and Research Agenda

**DOI:** 10.7448/IAS.18.4.20527

**Published:** 2015-07-20

**Authors:** Carlos F Cáceres, Kenneth H Mayer, Rachel Baggaley, Kevin R O’Reilly

## Introduction

The emergence of the HIV pandemic marked the end of the 20th century in many ways, including an unprecedented global effort to reduce HIV transmission and related mortality and to mitigate its social consequences. Like the pandemic itself, the HIV response became increasingly global, reinforced by extraordinary community involvement, trans-disciplinary efforts and a dynamic relationship between evidence, policy making and human rights principles. Early in the new century, in 2001, the UN General Assembly Special Sessions’ agreements defined the moral need to extend the benefits of effective HIV treatment, already available in high-income countries, to lower- and middle-income countries, which were home to the majority of people living with HIV. Such mechanisms as the Global Fund to Fight AIDS, Tuberculosis and Malaria and the US President's Emergency Plan for AIDS Relief were crucial to support countries’ efforts to scale up treatment.

A decade later, the number of people on antiretroviral treatment (ART) globally had increased significantly, with an associated reduction in HIV-related morbidity and mortality, changing the life perspectives of many generations in the most affected countries. However, despite the now well-recognized preventive effects of ART in suppressing HIV infectiousness in treated patients, HIV incidence remains unacceptably high. This is particularly true in subgroups of the general population in generalized epidemic settings (e.g. young women in Africa) and in key populations [KPs; e.g. men and transgender women (TW) who have sex with men, injection drug users and sex workers] in both concentrated and generalized epidemics. KPs are characterized not only by their high HIV incidence but also by the existence of laws, stigma, discrimination and social and economic exclusion (i.e. structural barriers that limit their access to prevention and care services and increase their social vulnerability to disease). It is increasingly clear that the response to HIV will not be sustainable if the number of infections is not significantly reduced in all affected populations.

## Discussion

For the past two decades, research has been ongoing to identify new biomedical strategies to prevent HIV infection. In the past few years, some such studies have demonstrated the efficacy of more potent combination ART-based regimes to prevent perinatal transmission, voluntary male medical circumcision to prevent HIV acquisition by men through vaginal intercourse and ART initiation at higher CD4 levels among people living with HIV to prevent HIV transmission to serodiscordant partners. The most recent addition to this list is oral pre-exposure prophylaxis (PrEP) to prevent HIV sexual transmission through anal and/or vaginal intercourse, regardless of partners’ gender.

After the development of a PrEP concept and conduct of animal studies, several trials assessed the efficacy of oral PrEP based on the daily usage of tenofovir (TDF) or tenofovir/emtricitabine (TDF/FTC) among people at high risk, and showed that if users adhered to the regime, preventive efficacy was very high. Concerns about the inability to ensure adherence among real-life users have receded after the release, in early 2015, of results from the PROUD trial. This trial, undertaken in sexual health clinics in the United Kingdom among men who have sex with men (MSM) at high risk, showed both high adherence and high effectiveness in a real-world setting.

In 2012, WHO released a conditional recommendation for PrEP use among seronegative partners in serodiscordant couples and among members of KPs, and suggested the implementation of demonstration studies to carefully identify issues relevant in potential PrEP implementation. In 2014, WHO released a strong recommendation for the inclusion of PrEP as an additional prevention choice in combination prevention packages oriented to MSM, making it clear that combination prevention includes not only a combination of individual-level biomedical and behavioural strategies, but also the conduct of interventions to remove structural barriers to prevention. The concept of alternative PrEP strategies (i.e. using different drugs besides tenofovir, and variations in the timing and forms of PrEP) is gaining increasing support and continues to be the focus of ongoing research.

Of note, so far oral PrEP uptake has been slower than might be expected from the magnitude of potential benefit. Moreover, it has been the focus of controversy among stakeholders. Some implementers and policy makers in various countries have raised concerns about potential low adherence leading to low effectiveness and drug resistance, “behavioural disinhibition” (people increasing risk taking because of perceived protection) leading to lower impact, potential drug toxicity, and high cost leading to low sustainability and competition with treatment. Advocates of prevention among sex workers and people who inject drugs (PWID) have argued that authoritarian states could implement mandatory PrEP programmes for KPs, resulting in human rights violations, or simply in the neglect of other effective prevention interventions, such as harm reduction strategies for PWID and condom programming for sex workers. In any event, if PrEP is to be considered as an additional prevention option for inclusion within a comprehensive HIV programme, then engaging with affected communities, and acknowledging that PrEP use is a choice that will only be appropriate for and desired by some people, will be fundamental.

Before PrEP becomes a feasible prevention strategy in specific settings, its potential impact within combination prevention programmes must be more clearly assessed and articulated (e.g. how it could contribute to reduction in incidence most efficiently and when it would be most cost-effective in combination prevention packages). Likewise, political, legal, economic and social issues raised by PrEP (e.g. commitment, sustainability, feasibility, acceptability and equal access) will have to be better understood and, if possible, resolved, and regulatory issues will have to be addressed. If PrEP is to be optimally implemented, strategies designed to improve adherence, monitor drug safety and use it better in a combination prevention framework must be assessed at the population level in the context of the opportunities and challenges faced by the health systems and the communities where it will be provided.

Achieving those goals will require effective dissemination of new evidence in a number of areas and further implementation research from various disciplinary perspectives (i.e. social science, policy and ethics; health systems; and economics, including modelling of cost, effectiveness and cost-effectiveness, among others). Discussion of new evidence on those topics, as well as case studies of potential PrEP implementation in diverse environments, could improve the scope of parameters available for decision making at present. For example, the inclusion of PrEP in public programmes in countries where resources are limited will require guidance on how to focus this new intervention based on geography, population group and/or individual risk to maximize impact and cost-effectiveness.

In light of the needs just described, NEMUS and WHO sought to contribute to a scholarly discussion of current conditions for PrEP implementation, social science issues, potential impact and efficiency and country case studies. This would take place through the preparation of a series of white papers for publication as a special issue of a peer-reviewed journal. A number of global leaders in HIV research and policy agreed to collaborate in this series, and papers were written between August 2014 and April 2015 for final publication in *JIAS* in mid-2015. The papers identify and cover the main aspects of the complex yet promising discussions around PrEP implementation today. In the remainder of this paper, we summarize the content of this special issue.

The opening contribution, by Cáceres *et al*. [1], starts with a historical account of the research and contextual elements that provided the evidence and interpretations guiding the recent or present (global) debates around PrEP. It then moves to analyse the key issues of this debate, based on the main arguments expressed, and to define which among them seem fundamental and yet remain unresolved. Finally, it summarizes the challenges, the opportunities and the pending tasks to ensure that PrEP is given balanced consideration in the combination prevention framework, in local responses as well as globally.

The second paper, by Cremin *et al*. [2], uses mathematical modelling to estimate the potential utility of a seasonal PrEP regimen whereby female partners of miners in Gaza, Mozambique, receive PrEP during December when their partners return home from the mines. They conclude that, given the potential for increased HIV transmission between miners returning in December and their partners in this setting (as expressed by the high numbers of pregnancies observed in this population some months after this visit), PrEP use by the latter could be a useful means of HIV prevention, and perhaps the only option for couples who wish to conceive.

Also using mathematical modelling, the third contribution, by Ying *et al*. [3], evaluates the cost-effectiveness of PrEP compared with expanding ART provision for HIV treatment to prevent incident HIV cases, an important question for policy makers considering PrEP implementation. They conducted micro-costing and time and motion analyses to estimate the real-world delivery costs of PrEP in an open-label prospective study of PrEP and ART delivery targeted to high-risk serodiscordant couples in Uganda (the Partners Demonstration Project). They found that, if implemented in public clinics, the annual cost of PrEP as a bridge to ART per high-risk serodiscordant couple is less than $100. They concluded that PrEP for people at high risk has the potential to cost-effectively prevent HIV infections in high-prevalence settings.

The next contribution in this series, by Auerbach and Hoppe [4], starts by acknowledging that PrEP raises a number of important social and psychological questions that must be attended if PrEP scale-up is sought as part of combination prevention, and especially if population-level impact is expected. They assess the subjective and social meanings of PrEP and its relationship with notions of safety, trust and control/power. Rather than providing answers to specific questions, they display the variety of current and potential issues raised by PrEP implementation, and call for increasing engagement of social scientists in their analysis.

The next four contributions constitute localized case studies that assess the feasibility and potential impact of PrEP strategies.

Venter *et al*. [5] assess the feasibility of PrEP implementation in southern Africa. The authors conclude that such implementation is, in principle, feasible, but recognize the remaining uncertainty of how to implement this strategy so that the populations most in need can be reached urgently for the greatest impact. They suggest the selection of specific risk groups and service environments in which PrEP can be distributed safely and cost-effectively while being mindful of ethical issues.

Providing a case study from Thailand, Colby *et al*. [6] start by recognizing that HIV has re-emerged in Thailand, resulting in very high prevalence and incidence among MSM, TW and, to a lesser extent, PWID. Given the conduct of clinical trials to assess efficacy in the recent past, PrEP may play a role for some higher-HIV-risk populations in Thailand and other countries in Asia experiencing similarly high incidence among specific KPs. Moreover, they consider that PrEP demonstration projects, as well as clinical trials of alternative PrEP regimes slated to begin in 2015 in Thailand, will provide additional data and experience on how to implement PrEP for individuals at high risk in the community. They conclude by stating that despite significant remaining challenges to the wider use of PrEP, it holds promise as a highly effective additional method for use as part of a combined HIV prevention strategy for MSM and TW in Thailand.

Veloso *et al*. provide a case study from Brazil [7]. They focus on a number of challenges and opportunities to incorporate PrEP within the continuum of HIV care and prevention for MSM and TW in Brazil. In their view, the universal access to health care provided through the Brazilian Unified Health System and the range of prevention and care services available country-wide to HIV-positive and at-risk MSM and TW are main facilitators for the implementation of a PrEP programme in Brazil. Simultaneously, low levels of PrEP awareness among MSM and TW and health care providers, low HIV testing frequency and low HIV risk perception among MSM and TW are core challenges to be addressed. They conclude by stating the potential importance of ongoing demonstration projects to resolve remaining challenges for PrEP implementation in the Brazilian context.

Then, Mayer *et al*. [8] analyse the case of the United States, which has been, in a way, the epicentre of PrEP research initiatives and discussions. They state that since the initial approval of the use of TDF/FTC for anti-HIV PrEP by the US Food and Drug Administration in 2012, uptake was initially limited to early adopters, but more recent community surveys and expert opinion suggest wider acceptance in some KPs. They explain that demonstration projects are underway to determine the best practices for reaching racial and ethnic minority communities, youth and at-risk heterosexuals in primary care settings, as well as sexually transmitted infection clinics. They also describe the challenges posed by clinicians who feel unprepared to prescribe PrEP, as well as by situations where PrEP is not covered by health insurance programmes. They describe current efforts to address those issues, and conclude that PrEP implementation in the United States is a work in progress, with increasing awareness and uptake among some individuals in KPs.

The following paper, by Celum *et al*. [9], focuses on the increased risk faced by adolescent and young women in Africa, in part due to contextual factors (e.g. gender norms and relationship dynamics, limited access to reproductive and sexual health services). Authors reviewed behavioral, economic and biomedical approaches to HIV prevention for this population, emphasizing the barriers, opportunities and implications for implementing PrEP in this group. They found: (1) behavioral interventions have had limited impact in part due to not effectively addressing the context, broader sexual norms and expectations, and structural factors that increase risk and vulnerability; and that (2) of available biomedical strategies, daily oral PrEP has the greatest evidence for protection, although adherence was low in two placebo-controlled trials in young African women. So they conclude by stating that social marketing, adherence support and behavioral economic interventions could be incorporated into oral PrEP demonstration projects among young African women to increase demand and optimize uptake and effective use of oral PrEP.

The final paper in this series, by Hankins *et al*. [10], starts by considering the unexpected difficulties in potential PrEP scale-up after the successful conclusion of PrEP clinical trials, and explains that such difficulties reflect the complexity of integration of PrEP schemes within combination HIV prevention strategies. They explore the principles of ethics that can inform resource allocation decision making anchored in distributive justice concerning the introduction of PrEP at a time when universal access to ART remains to be assured. They also describe the current regulatory situation with respect to TDF/FTC and its cost in low- and middle-income countries and cost-effectiveness in different populations at higher risk of HIV exposure. Finally, they describe the role of advocacy in moving the PrEP agenda forward. They conclude that PrEP has the potential to contribute significantly to HIV prevention as long as it is tailored to those who can most benefit from it and if current regulatory and pricing barriers can be overcome.

## Conclusions

In our view, this is a collection of timely contributions from global leaders in HIV research and policy which addresses geographic diversity, adopts a trans-disciplinary stance and covers a variety of the complex issues raised by PrEP. As it will become accessible to all, we honestly hope that it will remain a valuable resource for policy makers, programme managers, researchers and activists around the world at a moment of a paradigm shift of the global response to HIV.
