# Supplementary material for: PrEP Implementation Science: State-of-the-Art and Research Agenda
Source: J Int AIDS Soc. 2015 Jul 20;18(4Suppl 3):20527. doi: 10.7448/IAS.18.4.20527 (PMC4581083; doi:10.7448/IAS.18.4.20527)
Supplement: Related texts

# Sub article: PrEP implementation: moving from trials to policy and practice

**DOI:** 10.7448/IAS.18.4.20222

**Published:** 2015-07-20

**Authors:** Carlos F Cáceres, Kevin R O'Reilly, Kenneth H Mayer, Rachel Baggaley

**Affiliations:** 1 Center for Interdisciplinary Studies in Sexuality, AIDS and Society, Cayetano Heredia University, Lima, Peru; 2 HIV Programme, World Health Organization, Geneva, Switzerland; 3 Fenway Health, Beth Israel Deaconess Medical Center Boston, Harvard Medical School, Boston, MA, USA

**Keywords:** HIV, PrEP, key populations, scale-up, implementation science

## Abstract

**Introduction:**

It is increasingly clear that the HIV response will not be sustainable if the number of infections is not significantly reduced.

**Discussion:**

For two decades, research has been ongoing to identify new behavioural and biomedical strategies to prevent HIV infection. In the past few years, the efficacy of several new strategies has been demonstrated, including oral pre-exposure prophylaxis (PrEP; i.e. daily use of tenofovir/emtricitabine). Because several social, political and logistic barriers remain, however, optimal PrEP implementation will require a better dissemination of new evidence in a number of areas and additional implementation research from various disciplinary perspectives (i.e. social science, policy and ethics; health systems; and economics, including cost-effectiveness studies). Discussion of new evidence on those topics, as well as case studies of potential PrEP implementation in diverse environments, can improve the understanding of the role that PrEP may play in addressing the global HIV/AIDS epidemic.

In light of these needs, the Network for Multidisciplinary Studies in ARV-based HIV Prevention (NEMUS) and the World Health Organization (WHO) were honoured to co-organize a special issue of *JIAS* aimed at contributing to a scholarly discussion of current conditions surrounding PrEP implementation, potential impact and efficiency, social science concerns and the study of PrEP implementation in specific country cases. The papers included in this monograph identify and cover many of the main aspects of the complex yet promising discussions around PrEP implementation today.

**Conclusions:**

This is a collection of timely contributions from global leaders in HIV research and policy that addresses geographic diversity, uses a trans-disciplinary approach and covers a variety of the complex issues raised by PrEP. As this publication will become accessible to all, we hope that it will remain a valuable resource for policy makers, programme managers, researchers and activists around the world at a moment of a paradigm shift of the global response to HIV.

## References

[1] Cáceres CF, Koechlin F, Goicochea P, Sow P-S, O'Reilly K, Mayer K (2015). The promises and challenges of pre-exposure prophylaxis as part of the emerging paradigm of combination HIV prevention. J Int AIDS Soc.

[2] Cremin I, Morales F, Jewell BL, O'Reilly K, Hallett TB (2015). Seasonal PrEP for partners of migrant miners in southern Mozambique: a highly focused PrEP intervention. J Int AIDS Soc.

[3] Ying R, Heffron R, Celum C, Baeten JM, Katabira E, Bulya N (2015). Cost-effectiveness of pre-exposure prophylaxis targeted to high-risk serodiscordant couples as a bridge to sustained ART use in Kampala, Uganda. J Int AIDS Soc.

[4] Auerbach JD, Hoppe TA (2015). Beyond “getting drugs into bodies:” Social science perspectives on pre-exposure prophylaxis for HIV. J Int AIDS Soc.

[5] Venter F, Cowan F, Black V, Rebe K, Bekker LG (2015). Pre exposure prophylaxis in Southern Africa: feasible or not?. J Int AIDS Soc.

[6] Colby DJ, Srithanaviboonchai K, Vanichseni S, Ongwandee S, Phanuphak N, Martin MM (2015). HIV pre-exposure prophylaxis and health and community systems in the Global South: Thailand case study. J Int AIDS Soc.

[7] Veloso V, Mesquita F, Grinsztejn B (2015). Pre-exposure prophylaxis for men and transgender women who have sex with men – opportunities and challenges for implementation in Brazil. JIAS.

[8] Mayer KH, Hosek S, Cohen S, Liu AL, Pickett J, Warren M (2015). Antiretroviral pre-exposure prophylaxis implementation in the United States: a work in progress. J Int AIDS Soc.

[9] Celum CL, Delany-Moretlwe S, McConnell M, van Rooyen H, Bekker L-G, Kurth A (2015). Rethinking HIV prevention to prepare for oral PrEP implementation for young African women. J Int AIDS Soc.

[10] Hankins C, Macklin R, Warren M (2015). Translating PrEP effectiveness into public health impact: key considerations for distributive justice, access, availability, and affordability. J Int AIDS Soc.

# Sub article: The promises and challenges of pre-exposure prophylaxis as part of the emerging paradigm of combination HIV prevention

**DOI:** 10.7448/IAS.18.4.19949

**Published:** 2015-07-20

**Authors:** Carlos F Cáceres, Florence Koechlin, Pedro Goicochea, Papa-Salif Sow, Kevin R O'Reilly, Kenneth H Mayer, Peter Godfrey-Faussett

**Affiliations:** 1 Center for Interdisciplinary Studies in Sexuality, AIDS and Society, Universidad Peruana Cayetano Heredia, Lima, Peru; 2 Network for Multidisciplinary Studies in ARV-Based HIV Prevention (NEMUS), Lima, Peru; 3 HIV Department, World Health Organization, Geneva, Switzerland; 4 Center for AIDS Prevention Studies, University of California – San Francisco, San Francisco, CA, USA; 5 The Bill and Melinda Gates Foundation, Seattle, WA, USA; 6 The Fenway Institute, Fenway Health, Boston, MA, USA; 7 Joint United Nations Programme on HIV/AIDS, Geneva, Switzerland

**Keywords:** HIV prevention, pre-exposure prophylaxis, public health, health policy, antiretrovirals

## Abstract

**Introduction:**

Towards the end of the twentieth century, significant success was achieved in reducing incidence in several global HIV epidemics through ongoing prevention strategies. However, further progress in risk reduction was uncertain. For one thing, it was clear that social vulnerability had to be addressed, through research on interventions addressing health systems and other structural barriers. As soon as antiretroviral treatment became available, researchers started to conceive that antiretrovirals might play a role in decreasing either susceptibility in uninfected people or infectiousness among people living with HIV. In this paper we focus on the origin, present status, and potential contribution of pre-exposure prophylaxis (PrEP) within the combination HIV prevention framework.

**Discussion:**

After a phase of controversy, PrEP efficacy trials took off. By 2015, daily oral PrEP, using tenofovir alone or in combination with emtricitabine, has been proven efficacious, though efficacy seems heavily contingent upon adherence to pill uptake. Initial demonstration projects after release of efficacy results have shown that PrEP can be implemented in real settings and adherence can be high, leading to high effectiveness. Despite its substantial potential, beliefs persist about unfeasibility in real-life settings due to stigma, cost, adherence, and potential risk compensation barriers.

**Conclusions:**

The strategic synergy of behavioural change communication, biomedical strategies (including PrEP), and structural programmes is providing the basis for the combination HIV prevention framework. If PrEP is to ever become a key component of that framework, several negative beliefs must be confronted based on emerging evidence; moreover, research gaps regarding PrEP implementation must be filled, and appropriate prioritization strategies must be set up. Those challenges are significant, proportional to the impact that PrEP implementation may have in the global response to HIV.

## Introduction

By the end of the twentieth century, two decades after the HIV epidemic became visible, substantial success in slowing down HIV transmission had been achieved with ongoing prevention strategies, but it seemed far from stopping epidemic growth [1, 2]. Conversely, the use of combined antiretroviral treatment (ART) to prevent disease progression [3] and the development of effective regimes to prevent mother-to-child transmission (PMTCT) [4] were major steps in the HIV response.

As a corollary to “highly active antiretroviral treatment,” people discussed the possibility of “highly active prevention” [5]. The development of effective antiretrovirals (ARVs) led, from the start, to conceiving of biomedical prevention tools to decrease either susceptibility in uninfected people or infectiousness among people living with HIV (PLH). Given the lack of an effective HIV vaccine, key trials addressed several testable strategies that applied biomedical principles to HIV prevention, including sexually transmitted infection (STI) control, male medical circumcision, and the oral or topical use of ARV drugs to reduce susceptibility among the uninfected [6–14], as well as earlier ART initiation among positives to reduce infectivity [15]. Over a decade later, several new prevention strategies have been proven efficacious, and others are very promising [16, 17]. Among newly available technologies, evidence of real-life effectiveness is quickly accumulating for oral PrEP.

Simultaneously, in line with the WHO Social Determinants of Health approach [18], HIV risk is now understood as resulting from individual, interpersonal, community-level, and social-structural factors [19, 20]. In response to those, “structural interventions” targeting legal, institutional, social, cultural, and economic determinants of HIV vulnerability are considered essential to the HIV response [21].

This new environment of bio-behavioural, individual-level interventions, along with appropriate social-structural strategies, is consolidating a framework for combination prevention [22–24]. In this paper, we focus on the origin, present status, and potential contribution of PrEP within the combination HIV prevention framework.

## Discussion

### Pharmacologic prophylaxis in public health and the concept of HIV PrEP

Over the past few decades, the use of specific medications to prevent clinical conditions has become standard practice in preventive medicine [25–32]. Within the HIV field, secondary prophylaxis against *Pneumocystis carinii* pneumonia with cotrimoxazole was introduced among PLH [33]. After several evolving regimes, in 2013 WHO recommended that all women living with HIV found to be pregnant should start regular ART, whereas newborns should receive a six-week ARV course [34]. Over time, PMTCT approaches and successes have played a key role in shaping the initial thinking about using PrEP to prevent sexually acquired HIV infections.

### Early controversies around oral PrEP clinical trials

An overview of the complex history of ideas and research that contributed to the development of PrEP is relevant as it may, in part, explain some remaining controversies [35]. After animal studies demonstrated the protective effects of pre-exposure ARV use and successful early phase human studies demonstrated safety [36–38], the planning of phase III trials to assess the efficacy of oral PrEP using tenofovir (tenofovir disoproxil fumarate (TDF), Viread^®^) generated interest among HIV prevention scientists, but also some concerns among other stakeholders. Table 1 shows a list of planned PrEP trials in 2004 and 2005, including four trials terminated before or after enrollment had started.

**Table 1:** First generation of HIV pre-exposure prophylaxis trials

Sponsor	Place, expected start date	Population	Exposure	Sample size	Study aim	Duration (months)	Status
NIH/FHI	Cambodia, 2004	Women	Vaginal	960	Safety and efficacy	12	Stopped before start
FHI	Ghana, 2005	Women	Vaginal	400	Safety	12	Completed
FHI	Nigeria, 2005	Women	Vaginal	400	Safety	12	Stopped after enrolling 120
FHI	Cameroon, 2005	Women	Vaginal	400	Safety	12	Stopped after enrolling 400
FHI	Malawi, 2005	Heterosexual men	Penile	400	Safety	12	Stopped before start
CDC	Thailand, 2005	PWID	Parenteral	1200	Safety and efficacy	12	Completed
CDC	Botswana, 2005	Heterosexual men and women	Vaginal/penile	1600	Safety and efficacy	18	Completed
CDC	San Francisco, Atlanta, Boston, USA; 2005	MSM	Penile/rectal	400	Safety	15	Completed
NIH	Peru/Ecuador, 2007	MSM	Penile/rectal	1400	Safety and efficacy	18	Completed

NIH, National Institutes of Health; FHI360, Family Health International; CDC, US Center for Disease Control, PWID, people who inject drugs; MSM, men who have sex with men.

Four trials planned for implementation in Cambodia, Cameroon, Nigeria, and Malawi were cancelled due to controversies. The Cambodia trial, focused on PrEP safety and efficacy among female sex workers, never started due to concerns that, if women became HIV-infected, they would not have access to lifelong treatment, while losing their source of livelihood [35, 39–42]. In early 2005, similar studies in women at high HIV risk were cancelled in Cameroon (due to allegations of inappropriate standards [43]) and Nigeria (where the sponsor considered that conditions for study conduct were inappropriate [35, 37, 44]). In Malawi, the government stopped the trial due to fear of drug resistance developing if participants became infected during implementation [44]. The trial planned among people who injected drugs (PWID) in Thailand risked cancellation due to stakeholders’ allegations of unethical standards (i.e. not offering clean needles and syringes, in addition to methadone, according to WHO guidelines) [45]. The trial finished in May 2011 [12], but PWID organizations have reiterated that the trial was unethical and its conclusions are not acceptable [46].

In May 2005, a stakeholder consultation was convened with the participation of trial funders and community representatives from Cameroon, Ghana, Malawi, and Thailand [47]. Participants agreed that an immediate evaluation of trial design and protocol procedures was needed in ongoing and future trials to ensure compliance with the highest standards of care, civil society participation in trial design and conduct, and availability of mechanisms for feedback and conflict resolution at study sites [44]. Addressing one of the recommendations, the Joint United Nations Programme on HIV/AIDS, in collaboration with Global Advocacy for HIV Prevention (AVAC), consulting with communities, revised and produced a series of guidelines [48–52] for ethical assessment and definition of appropriate standards of study conduct in international HIV research. It conveyed the message that the scientific community had addressed the concerns raised about the ongoing trials, and that, together with communities, a new framework of operation had been established.

### Implementation of the major oral PrEP efficacy trials: mixed results and lessons learned

Increasing global access to ARTs and better-tolerated single and combined formulations, together with resolution of the initial controversies, eventually resulted in the implementation of PrEP trials. Some of the delayed PrEP trials, along with new ones, were rolled out between 2007 and 2013. Those trials included the following: two among women only (i.e. FEM-PrEP [53] and VOICE [54]), in addition to a vaginal gel study (CAPRISA 004 [9]); two among heterosexuals (i.e. Partners PrEP in serodiscordant couples and TDF2 in heterosexuals at high risk [12, 55]); one among men who have sex with men (MSM) and transwomen (iPrEx) in the Americas, South Africa, and Thailand [56]; and one among PWID (the CDC BTS) in Thailand [13]. Given concerns for resistance based on results of a preclinical study in macaques [57], the investigators of iPrEx and TDF2 decided to use, instead of tenofovir alone, the combination of tenofovir and emtricitabine (as offered in the formulation of Truvada^®^ by Gilead); while Partners PrEP opted to have separate arms for TDF and tenofovir - emtricitabine (TDF-FTC) for comparison. Table 2 shows the list of oral PrEP trials conducted, as well as their mixed results.

**Table 2:** PrEP randomized controlled trials and their findings

Study (reference)	Location	Population	Efficacy	

			Point estimate (%)	95% CI
iPrEx (Grant *et al*. 2010)	Peru, Ecuador, Brazil, United States, South Africa, Thailand	MSM	42	18 to 60%
Partners PrEP (Baeten *et al*. 2011)	Kenya, Uganda	Men	84	49 to 94%
		Women	66	19 to 82%
TDF2 (Thigpen *et al*. 2012)	Botswana	Men	80	25 to 97%
		Women	49	22 to 81%
FEM-PrEP (Van Damme *et al*. 2012)	Kenya, Tanzania	Women	6	−52 to 42%
VOICE (Marazzo *et al*. 2013)	South Africa, Uganda Zimbabwe	Women	−4	−50 to 30%
The CDC BTS (Choopaya *et al*. 2013)	Thailand	PWID	49	10 to 72%
Ipergay (Molina *et al*. 2015)	France, Canada	MSM	86	39 to 98%
PROUD (McCormack *et al*. 2015)	United Kingdom	MSM	86	58 to 96%

The protective effects of the first three PrEP trials (Table 2) completed in 2010 to 2011 (i.e. iPrEx, Partners PrEP, and TDF2) [12, 55] generated optimism. The subsequent termination of FEM-PrEP and VOICE due to futility [53, 54], however, led to assessments of the potential sources of such variability. Data on ARV concentration in serum, plasma, PBMCs, and hair showed highly variable adherence within and across sites, which likely explained important differences between intent-to-treat findings and those controlling for effective dose exposure. Overall adherence was extremely low in FEM-PrEP and VOICE, explaining their outcomes [58], as there is no evidence of interference of oral contraceptives in the protective effect of oral PrEP. Retrospective analyses that used mathematical modelling on those data showed that efficacy was strongly associated with detectable drug in serum or tissues. High adherence was associated with over 99% protection in iPrEx [59, 60]. Importantly, these analyses also showed the presence of “forgiveness”: oral PrEP is probably protective with less than daily dosing (although with no less than four doses per week), and such forgiveness may be lower in women due to relatively lower concentrations in vaginal tissue, compared to rectal tissue, after the same oral dose [61].

### Oral PrEP and effectiveness from subsequent studies

Low adherence levels in the efficacy trials raised concerns about the feasibility of PrEP as a public health strategy. Nevertheless it was recognized that real-life adherence to a product of demonstrated effectiveness would probably be different from adherence in a placebo-controlled trial, where participants are told that intervention efficacy is still unclear and that half of them are receiving a placebo [62]. This effect was demonstrated by the open label extension of iPrEx [63], where high levels of adherence were reported, and PrEP reduced incidence among those who consistently took the medication. Likewise, in October 2014, the UK PROUD study of immediate versus delayed PrEP for MSM accessing services at UK sexual health clinics stopped the deferred treatment arm and offered PrEP to all participants, given the protection demonstrated in their ongoing pilot study [64]. Two weeks later, the French Ipergay trial of intermittent, pericoital PrEP terminated the placebo arm based on an interim analysis that showed adherence and “considerable efficacy” [65]. In February 2015, findings from both studies showed similar (86%) effectiveness in preventing HIV infection among MSM at increased risk, who overall showed high adherence. The overall picture is that MSM who are motivated to use PrEP can achieve sufficient adherence to have even greater reduction in HIV as compared to iPrEx findings [66].

In a different epidemic context (i.e. serodiscordant couples in generalized epidemic settings), the Partners Demonstration Project, an open label observational study of PrEP and early treatment in Kenya and Uganda, also showed an overall relative risk reduction of 96% in an interim analysis [67]. These results suggest the use of PrEP as a bridge in serodiscordant couples – whereby the HIV-negative partner takes PrEP for protection while waiting for the HIV-positive partner to start treatment and minimize viral load. Several demonstration projects are starting in other countries, many of which are focusing on female sex workers (FSWs), namely, in Benin, India, Kenya, Senegal, South Africa, and Zimbabwe. It is becoming clear that these demonstration projects will help design PrEP implementation plans as part of combination prevention in programmatic contexts.

### The post-trial context of PrEP: effective need, programmatic dilemmas, and social paradoxes

As of early mid-2015, evidence supporting PrEP effectiveness could justify more active scaling up. However, should PrEP ever become an important component of the global HIV response, several issues need to be tackled.

#### Concerns prior to trial outcomes

Some early concerns have not been fully addressed or have adopted new dimensions. First, PrEP raised substantial resistance because it destabilized the social norm of “100% condom use,” which prevented so many infections in three decades, while in fact that social norm had already started to recede [68, 69]. Second, PrEP was misunderstood as intended to replace condoms, while in fact it was meant to become one element (but never the sole element) of the emerging paradigm of combination prevention [23]. Third, many objected to a perceived medicalization of HIV prevention, although this perception can be interpreted as fear of turning prevention into a mechanical process with no social-structural component; recent studies have shown that successful PrEP implementation retains the need for social interaction and the importance of community buy-in. Fourth, the controversies surrounding the early phases of the international PrEP trials led to fears that PrEP would be implemented in a compulsory way among key populations (e.g. sex workers) or serve as an excuse to not provide basic prevention tools (e.g. harm reduction for injection drug users), paying no attention to human rights [70–74]. From reactions so far, because PrEP is not cheap, its compulsory use seems unlikely with key populations anywhere. Fifth, PLH organizations and some policy makers have feared competition with treatment in an era of decreased resources, although it now seems clear that the PrEP component of HIV response, in order to remain cost-effective, should be focused on small fractions of the population at very high risk, while different strategies should be used with others [71]. Finally, PrEP may have generated a “moral panic” in certain stakeholders concerned about a potential loss of sexual restraints, leading to so-called risk compensation (i.e. having riskier sex and thereby neutralizing the benefit of PrEP). Even within the gay community, this concern has created a certain stigma affecting PrEP [75]. With the current media focus on PrEP and MSM, many assume that MSM should “be responsible and just use condoms,” which provide sufficient protection to them. This view fails to take into account the following: 1) for many MSM, condoms are not a feasible option, for several reasons, including loss of pleasure and power dynamics in relationships [76, 77]; 2) a more nuanced discussion is missing about the potential benefits of PrEP for women, including female sex workers and transwomen, for whom PrEP offers a prevention strategy that is under their control [78–80].

#### Public health and clinical guidance

In 2012, through its standard guideline development procedure, the WHO issued a conditional recommendation for PrEP use among serodiscordant couples and among men and transgender women who have sex with men, from a public health approach. It called for demonstration projects to assess conditions for potential PrEP implementation [81]. In 2014, the WHO updated its guidance and released a strong recommendation for governments to consider adding PrEP components to their combination prevention strategies for MSM in countries with high disease burden in those populations [23]. In the United States, a prophylactic indication for TDF-FTC for PrEP was approved in 2012 [82], and in early 2014 the US Center for Disease Control consolidated the indication of PrEP for people at risk for HIV acquisition [83].

Besides public health guidance (relevant for public prevention programmes set up by countries), clinical guidance is necessary where it is also recognized that PrEP is not meant to be used for life. As with any other prophylaxis, PrEP makes sense during periods of high exposure, which rarely cover an individual's entire life. Pericoital regimes, such as the focus of the Ipergay study [84], may also play a role in transitioning out of PrEP. The context in which oral PrEP may be individually recommended to some people may present some commonalities with the context in which other drugs are currently used and recommended for the prevention of other diseases. For example, statins are used to prevent cardiovascular disease, as they are essentially safe, like ARV drugs, but can rarely cause serious toxicity [85].

#### Population focus

PrEP is recommended for those facing a genuinely high risk of acquiring HIV. Clearly the benefit to be obtained from PrEP depends on the incidence rate of HIV and this factor has to be balanced against the (small) risks of the medication. Although adverse events are infrequent among positives and negatives alike, the benefits of treatment for those living with HIV are very high, whereas the benefits of PrEP to those who are HIV-negative depend entirely on their chance of acquiring infection. Based on available incidence estimations and behavioural data, PrEP use could have a clear positive impact among MSM almost everywhere, among sex workers in many places, among young people in southern Africa, but also among serodiscordant couples and those trying to conceive, and other key populations in various settings (e.g. partners of migrant labourers or truck drivers) [23]. Sound programmatic targeting is crucial to avoid PrEP ending up being prescribed mostly to the “worried well” [86]. Concerns remain as to how to target persons who would benefit from PrEP in generalized epidemics (potentially based on geographies with the highest incidence).

*Access* is determined by specific prevention guidance from normative bodies, drug availability, and a financing regime (e.g. out of pocket, insurance, public health programme). Concerns have been raised about the control that the pharmaceutical industry might have over the prices of PrEP regimes – which, at least in higher income countries, could be difficult to sustain [87–90]. Not without challenges, PrEP is slowly starting to be prescribed to at-risk Americans financed by medical insurance companies, national public health programmes for the poor or disabled, or through the producer of TDF-FTC, Gilead, via a drug assistance programme for underinsured individuals [91]. Some programmes are making PrEP a part of combination prevention, such as New York City's MSM programme [92] or the DREAMS initiative recently announced by U.S. President's Emergency Plan for AIDS Relief (PEPFAR) for 10 priority countries to provide a package of interventions aimed at tackling HIV among adolescent girls and young women [93].

Recent developments in European Union PrEP trials may accelerate regulatory changes there, too. However, TDF-FTC is not only not licensed for use as a preventive measure elsewhere, it is not even available for treatment in a few countries. In many countries, drugs can be used “off-label,” but usually only for acute indications with uncertain diagnosis or for life-threatening situations with no effective standard treatment, which would not be the case for PrEP. Nonetheless, demonstration projects ongoing in Brazil, several African countries, Thailand, and Australia, may lead to local approvals of TDF-FTC use for this indication. Finally, pricing is tied up with commercial decisions based on market estimations and trade agreements and also expressed in drug packaging and marketing.

#### The role of condoms

Given concerns about so-called risk compensation, normative bodies have decided to continue to maintain that PrEP should be used together with condoms [23, 83, 94]. However, initial data from demonstration studies in MSM show that people who choose to take PrEP may, in fact, be those who report episodes of unprotected anal intercourse, and their reported PrEP adherence is already high, with no subsequent risk compensation or change from their present condom use [95]. Hence, at least among MSM, PrEP may become a choice among people at risk due to condomless anal sex, who feel that a daily pill may suit them better than condoms. Perhaps a compromise in PrEP messaging could include stating the following: 1) PrEP does not intend to replace condoms but to add to condom protection; 2) PrEP does not protect against bacterial STIs; 3) PrEP can become especially useful for those who have difficulties with consistent condom use, as long as it is taken as prescribed.

#### Delivery

A few delivery models for local adaptation should be coming out soon from ongoing demonstration projects. It is likely, however, that some people who could benefit most from PrEP are those who find it most difficult to come routinely to a health service. Delivery models should be developed that are appropriate for the populations being served, while simultaneously being “fit for purpose” (e.g. they need to be integrated into the more holistic healthcare needs of the population, able to provide reliable HIV testing services, linked to HIV treatment services, able to detect serious toxicity, and able to refer complex or worrying cases into the broader health system) [96, 97]. Demonstration projects are evaluating delivery models. For example, PROUD delivered PrEP through sexual health clinics with quarterly visits in the United Kingdom [84, 98], whereas demonstration projects among sex workers in Benin and in South Africa are setting models where PrEP might work as part of a combination prevention package of PrEP and treatment as prevention (TasP). In India, PrEP is being evaluated to determine whether it can be implemented among brothel-based and street-based sex workers’ health services; and in Zimbabwe it is being offered in the context of highway-based sex work. US demonstration projects are evaluating customized prevention packages for MSM and transwomen (TW), for example, some that may include PrEP, the “testing and linking” of young MSM of colour to sexual health services, and text-messaging intervention to improve adherence.

#### Adherence, resistance to ARV and secondary effects

Practitioners feared that adherence in real life would be low (as in various trials), leading to resistance to a complex drug inappropriately used in primary care [99, 100]. Nevertheless, open label studies have shown that, among people who perceive its need, adherence can be very high [101]. Adherence must be a central message to users, despite the forgiveness shown by studies so far [59], where participants generally adhered well [63]. Because PrEP is given to HIV-uninfected people, it cannot cause resistance unless the person first acquires HIV and then continues to take PrEP. That is why it is essential to build delivery systems that reliably check the HIV status of those wanting to take or continue to take PrEP and that avoid informal distribution channels. Mathematical models show that most resistance comes from PLH who are not fully adherent to treatment; hence, preventing new infections through the use of PrEP could reduce rather than exacerbate levels of resistance in a community. Finally, stakeholders also feared drug toxicities and secondary effects [102, 103], but the experience so far has shown that they remain at reasonably low levels [104–107].

## Conclusions: perspectives and challenges in 2015 and beyond

A number of studies in the pipeline may streamline ARV-based prevention options even further, including the Ipergay trial (pericoital oral PrEP among MSM; this trial recently dropped the placebo arm [65]); the Ring Study and ASPIRE (designed to determine whether a monthly vaginal ring that delivers dapivirine helps prevent HIV infection in women and is safe for long-term use) [108, 109]; and studies of ARVs with long half-lives, such as rilpivirine and cabotegravir, to be administered parenterally every eight to twelve weeks [110, 111]. Such approaches to PrEP delivery may eventually become more widely applicable than oral PrEP, but it will be several years before they are manufactured, licensed, available, and affordable.

Although individual oral PrEP prescription may become the only form of PrEP available in many places, governments may implement population-focused PrEP programmes for cost-effectiveness, considering costs, affordability, and financing. However, decisions on focused PrEP programmes for populations with uncontrolled ongoing HIV transmission should preferably be based on impact. Given the high price of Truvada^TM^ in high income countries, PrEP programmatic feasibility will in part be defined by the pharmaceutical industry's role in providing access to supplies of TDF or TDF-FTC globally in the near future. The ongoing licensing and pricing of TDF-FTC in many places will be challenging, particularly in the context of new free-trade agreements [112]. Resolving current problems in treatment distribution in many countries and committing to ensuring its supply alongside the start of PrEP programmes will be central [113].

In HIV epidemics concentrated on MSM, self-selection of high-risk men insufficiently protected by condoms, with higher adherence to PrEP and no evidence of risk compensation, as observed in demonstration projects, suggests a desirable fit between a new tool and a population in need. However, it also demonstrates the importance of interdisciplinary studies and critical policy analysis to better understand how PrEP is actually adopted by at-risk communities and, under those conditions, to understand the following: what factors could improve or affect its effectiveness; how different forms of PrEP delivery would avert new infections and what the cost-effectiveness ratio would be; what role mathematical modelling could play in effectiveness and cost-effectiveness analysis; and how policy dialogue could be promoted to ensure that this strategy is considered fairly by governments.

In conclusion, over the years, important research findings have improved our understanding of biomedical and social determinants of HIV transmission. These findings have provided the evidence needed to transform the preventive response with the inclusion of “highly active” prevention approaches, as well as social and structural strategies at various levels, in what is now called a “combination prevention framework.” Among those prevention approaches, oral PrEP using TDF or TDF-FTC has emerged as an evidence-based option for people at risk of acquiring HIV. Despite its substantial potential, appropriate contribution of PrEP to the HIV response implies tackling two kinds of challenges: first, to clarify the numerous misconceptions that have led many to ignore the growing evidence of PrEP's utility; second, to fill research gaps concerning PrEP and its implementation and to resolve a number of issues related to its pertinence in different geographic and epidemiological contexts, health system structures and procedures, access, cost, and appropriate prioritization strategies. These are major challenges, proportional to the magnitude of the change we are witnessing in the dominant HIV prevention paradigm, one in which the impact of PrEP may finally help solidify the foundation of the so-far elusive concept of combination HIV prevention.

## References

[1] Kaufman MR, Cornish F, Zimmerman RS, Johnson BT (2014). Health behavior change models for HIV prevention and AIDS care: practical recommendations for a multi-level approach. J Acquir Immune Defic Syndr.

[2] Lettenmaier C, Kraft JM, Raisanen K, Serlemitsos E (2014). HIV communication capacity strengthening: a critical review. J Acquir Immune Defic Syndr.

[3] Detels R, Muñoz A, McFarlane G, Kingsley LA, Margolick JB, Giorgi J (1998). Effectiveness of potent antiretroviral therapy on time to AIDS and death in men with known HIV infection duration. Multicenter AIDS Cohort Study Investigators. JAMA.

[4] WHO, IATT, UNICEF (2013). Expanding and simplifying treatment for pregnant women living with HIV.

[5] Coates TJ, Richter L, Cáceres C (2008). Behavioural strategies to reduce HIV transmission: how to make them work better. Lancet.

[6] Auvert B, Taljaard D, Lagarde E, Sobngwi-Tambekou J, Sitta R, Puren A (2005). Randomized, controlled intervention trial of male circumcision for reduction of HIV infection risk: the ANRS 1265 trial. PLoS Med.

[7] Bailey RC, Moses S, Parker CB, Agot K, Maclean I, Krieger JN (2007). Male circumcision for HIV prevention in young men in Kisumu, Kenya: a randomised controlled trial. Lancet.

[8] Gray RH, Kigozi G, Serwadda D, Makumbi F, Watya S, Nalugoda F (2007). Male circumcision for HIV prevention in men in Rakai, Uganda: a randomised trial. Lancet.

[9] Abdool Karim Q, Abdool Karim SS, Frohlich JA, Grobler AC, Baxter C, Mansoor LE (2010). Effectiveness and safety of tenofovir gel, an antiretroviral microbicide, for the prevention of HIV infection in women. Science.

[10] Baeten JM, Donnell D, Ndase P, Mugo NR, Campbell JD, Wangisi J (2012). Antiretroviral prophylaxis for HIV prevention in heterosexual men and women. N Engl J Med.

[11] Grant R, Lama J, Anderson P, McMahan V, Liu A, Vargas L (2010). Preexposure chemoprophylaxis for HIV prevention in men who have sex with men. N Engl J Med.

[12] Thigpen MC, Kebaabetswe PM, Paxton LA, Smith DK, Rose CE, Segolodi TM (2012). Antiretroviral preexposure prophylaxis for heterosexual HIV transmission in Botswana. N Engl J Med.

[13] Choopanya K, Martin M, Suntharasamai P, Sangkum U, Mock PA, Leethochawalit M (2013). Antiretroviral prophylaxis for HIV infection in injecting drug users in Bangkok, Thailand (the Bangkok Tenofovir Study): a randomised, double-blind, placebo-controlled phase 3 trial. Lancet.

[14] Boyles S (1995). STD treatment reduces HIV in rural Tanzania. AIDS Wkly.

[15] Cohen MS, Chen YQ, McCauley M, Gamble T, Hosseinipour MC, Kumarasamy N (2011). Prevention of HIV-1 infection with early antiretroviral therapy. N Engl J Med.

[16] Hoffman IF, Taha TE, Padian NS, Kelly CW, Welch JD, Martinson FE (2004). Nonoxynol-9 100 mg gel: multi-site safety study from sub-Saharan Africa. AIDS.

[17] Buchbinder S, Mehrotra D, Duerr A, Fitzgerald D, Mogg R, Li D (2008). Efficacy assessment of a cell-mediated immunity HIV-1 vaccine (the step study): a double-blind, randomised, placebo-controlled, test-of-concept trial. Lancet.

[18] WHO

[19] Buot ML, Docena JP, Ratemo BK, Bittner MJ, Burlew JT, Nuritdinov AR (2014). Beyond race and place: distal sociological determinants of HIV disparities. PLoS One.

[20] Hirsch JS (2014). Labor migration, externalities and ethics: theorizing the meso-level determinants of HIV vulnerability. Soc Sci Med.

[21] Auerbach JD, Parkhurst JO, Caceres CF (2011). Addressing social drivers of HIV/AIDS for the long-term response: conceptual and methodological considerations. Glob Public Health.

[22] Hankins CA, Dybul MR (2013). The promise of pre-exposure prophylaxis with antiretroviral drugs to prevent HIV transmission. Curr Opin HIV AIDS.

[23] WHO, editor (2014). Policy brief: HIV prevention, diagnosis, treatment and care for key populations.

[24] Padian NS, Mccoy SI, Manian S, Wilson D, Schwartländer B, Bertozzi SM (2011). Evaluation of large-scale combination HIV prevention programs: essential issues. J Acquir Immune Defic Syndr.

[25] Biamonte MA, Wanner J, Le Roch KG (2013). Recent advances in malaria drug discovery. Bioorg Med Chem Lett.

[26] Upke IS, Moonasar D, Raman J, Barnes KI, Baker L, Blumberg L (2013). Case management of malaria: treatment and chemoprophylaxis. S Afr Med.

[27] Feldstein CA (2008). Statins as antihypertensives. Recent Pat Cardiovasc Drug Discov.

[28] Simko F, Pechanova O (2009). Potential roles of melatonin and chronotherapy among the new trends in hypertension treatment. J Pineal Res.

[29] Sepanlou SG, Farzadfar F, Jafari E, Danaei G (2012). Cardiovascular disease prevention using fixed dose pharmacotherapy in Iran: updated meta-analyses and mortality estimation. Arch Iran Med.

[30] Steyn DW, Steyn P (2007). Low-dose dopamine for women with severe pre-eclampsia. Cochrane Database Syst Rev.

[31] Genest DS, Falcao S, Michel C, Kajla S, Germano MF, Lacasse AA (2013). Novel role of the renin-angiotensin system in preeclampsia superimposed on chronic hypertension and the effects of exercise in a mouse model. Hypertension.

[32] Dorniak-Wall T, Grivell RM, Dekker GA, Hague W, Dodd JM (2014). The role of L-arginine in the prevention and treatment of pre-eclampsia: a systematic review of randomised trials. J Hum Hypertens.

[33] Podzamczer D, Santin M, Jimenez J, Casanova A, Bolao F, Gudiol GR (1993). Thrice-weekly cotrimoxazole is better than weekly dapsone-pyrimethamine for the primary prevention of Pneumocystis carinii pneumonia in HIV-infected patients. AIDS.

[34] WHO Consolidated guidelines on the use of antiretroviral drugs for treating and preventing HIV infection.

[35] Singh JA, Mills EJ (2005). The abandoned trials of pre-exposure prophylaxis for HIV: what went wrong?. PLoS Med.

[36] Grohskopf LA, Chillag KL, Gvetadze R, Liu AY, Thompson M, Mayer KH (2013). Randomized trial of clinical safety of daily oral tenofovir disoproxil fumarate among HIV-uninfected men who have sex with men in the United States. J Acquir Immune Defic Syndr.

[37] Peterson L, Taylor D, Clarke E, Doh A, Phillips P, Belai G (2006). Findings from a double-blind, randomized placebo-controlled trial of tenofovir disoproxil fumarate (TDF) for prevention of HIV infection in women.

[38] Mayer KH, Karim SA, Kelly C, Maslankowski L, Rees H, Profy AT (2003). Safety and tolerability of vaginal PRO 2000 gel in sexually active HIV-uninfected and abstinent HIV-infected women. AIDS.

[39] Lancet T (2005). The trials of tenofovir trials. Lancet.

[40] Page-Shafer K, Saphonn V, Penh Sun L, Chi vun M, Cooper DA, Kaldor JM (2005). HIV prevention research in resource-limited setting: the experience of planning a trial in Cambodia. Lancet.

[41] Cohen J (2005). More woes for novel HIV prevention approach. Science.

[42] Loff B, Jenkins C, Ditmore M, Overs C, Barbero R (2005). Unethical clinical trials in Thailand: a community response. Lancet.

[43] Mills E, Rachlis B, Wu P, Wong E, Wilson K, Singh S (2005). Media reporting of tenofovir trials in Cambodia and Cameroon. BMC Int Health Hum Rights.

[44] Cairns G, Alcorn K, Bernard EJ, Carter M, Smart T (2006). Preventing HIV.

[45] Chua A, Ford N, Wilson D, Cawthorne P (2005). The tenofovir pre-exposure prophylaxis trial in Thailand: researchers should show more openness in their engagement with the community. PLoS Med.

[46] AIDSMEDS (2013). Trial of PrEP in drug users called into question [Internet].

[47] IAS (2005). Stakeholder consultation to address issues related to tenofovir prophylactic research.

[48] UNAIDS (2000). Ethical considerations in HIV preventive vaccine research.

[49] UNAIDS (2012). Ethical considerationsin biomedical HIV prevention trials.

[50] UNAIDS (2007). Good participatory practice: guidelines for biomedical HIV prevention trials.

[51] UNAIDS (2011). Good participatory practice: guidelines for biomedical HIV prevention trials.

[52] UNAIDS (2004). Ethical considerations in HIV preventive vaccine research.

[53] Van Damme L, Corneli A, Ahmed K, Agot K, Lombaard J, Kapiga S (2012). Preexposure prophylaxis for HIV infection among African women. N Engl J Med.

[54] Marrazzo JM, Ramjee G, Richardson BA, Gomez K, Mgodi N, Nair G (2015). Tenofovir-based preexposure prophylaxis for HIV infection among African women. N Engl J Med.

[55] Baeten JM, Donnell D, Ndase P, Mugo NR, Campbell JD, Wangisi J (2012). Antiretroviral prophylaxis for HIV prevention in heterosexual men and women. N Engl J Med.

[56] Grant RM, Lama JR, Anderson PL, McMahan V, Liu AY, Vargas L (2010). Preexposure chemoprophylaxis for HIV prevention in men who have sex with men – supplemental appendix. N Engl J Med.

[57] Garcia-Lerma JG, Otten RA, Qari SH, Jackson E, Cong ME, Masciotra S (2008). Prevention of rectal SHIV transmission in macaques by daily or intermittent prophylaxis with emtricitabine and tenofovir. PLoS Med.

[58] Murnane PM, Heffron R, Ronald A, Bukusi EA, Donnell D, Mugo NR (2014). Pre-exposure prophylaxis for HIV-1 prevention does not diminish the pregnancy prevention effectiveness of hormonal contraception. AIDS.

[59] Anderson PL, Glidden DV, Liu A, Buchbinder S, Lama JR, Guanira JV (2012). Emtricitabine-tenofovir concentrations and pre-exposure prophylaxis efficacy in men who have sex with men. Sci Transl Med.

[60] Donnell D, Baeten JM, Bumpus NN, Brantley J, Bangsberg DR, Haberer JE (2014). HIV Protective efficacy and correlates of tenofovir blood concentrations in a clinical trial of PrEP for HIV prevention. J Acquir Immune Defic Syndr.

[61] Cortrell ML, Yang KH, Prince HMA, Sykes C, White N, Malone S (2014). Predicting effective Truvada PrEP dosing strategies with a novel PK-PD model incorporating tissue active metabolites and endogenous nuclootides (EN).

[62] Amico KR, Stirratt MJ (2014). Adherence to preexposure prophylaxis: current, emerging, and anticipated bases of evidence. Clin Infect Dis.

[63] Grant RM, Anderson PL, McMahan V, Liu A, Amico KR, Mehrotra M (2014). Uptake of pre-exposure prophylaxis, sexual practices, and HIV incidence in men and transgender women who have sex with men: a cohort study. Lancet Infect Dis.

[64] Medical Research Council Press Office (2014). PROUD study interim analysis finds pre-exposure prophylaxis (PrEP) is highly protective against HIV for gay men and other men who have sex with men in the UK [press release].

[65] ANRS UN grand succes dans la lutte contre le vih/SIDA. Un médicament pris au moment des rapports sexuels réduit efficacement le risque d'infection [Internet]. ANRS.

[66] Cohen S, Vittinghoff E, Anderson P, Doblecki-Lewis S, Bacon O, Chege W (2014). Implementation of PrEP in STD clinics: high uptake and drug detection among MSM in the demonstration project.

[67] Baeten J (2015). Near elimination of HIV transmission in a demonstration project of PrEP and ART.

[68] Guest G, Shattuck D, Johnson L, Akumatey B, Clarke EE, Chen PL (2008). Changes in sexual risk behavior among participants in a PrEP HIV prevention trial. Sex Transm Dis.

[69] Paxton LA (2012). Considerations regarding antiretroviral chemoprophylaxis and heterosexuals in generalized epidemic settings. Curr Opin HIV AIDS.

[70] O'Hara KM (2012). Pre-exposure prophylaxis: where HIV prevention and responsibility intersect. JAAPA.

[71] Bailey TC, Sugarman J (2013). Social justice and HIV vaccine research in the age of pre-exposure prophylaxis and treatment as prevention. Curr HIV Res.

[72] Philpott S (2013). Social justice, public health ethics, and the use of HIV pre-exposure prophylaxis. Am J Prev Med 1.

[73] Rowniak S, Portillo C (2013). Pre-exposure prophylaxis: an ethical discussion. J Assoc Nurses AIDS Care.

[74] Venter F, Allais L, Richter M (2014). Exposure ethics: does HIV pre-exposure prophylaxis raise ethical problems for the health care provider and policy maker?. Bioethics.

[75] Brooks RA, Landovitz RJ, Kaplan RL, Lieber E, Lee SJ, Barkley TW (2012). Sexual risk behaviors and acceptability of HIV pre-exposure prophylaxis among HIV-negative gay and bisexual men in serodiscordant relationships: a mixed methods study. AIDS Patient Care STDS.

[76] Johnson BT, Scott-Sheldon LA, Smoak ND, Lacroix JM, Anderson JR, Carey MP (2009). Behavioral interventions for African Americans to reduce sexual risk of HIV: a meta-analysis of randomized controlled trials. J Acquir Immune Defic Syndr.

[77] Albarracin D, Durantini MR (2010). Are we going to close social gaps in HIV? Likely effects of behavioral HIV-prevention interventions on health disparities. Psychol Health Med.

[78] Peng B, Yang X, Zhang Y, Dai J, Liang H, Zou Y (2012). Willingness to use pre-exposure prophylaxis for HIV prevention among female sex workers: a cross-sectional study in China. HIV/AIDS.

[79] Jackson T, Huang A, Chen H, Gao X, Zhang Y, Zhong X (2013). Predictors of willingness to use HIV pre-exposure prophylaxis among female sex workers in Southwest China. AIDS Care.

[80] Mack N, Evens EM, Tolley EE, Brelsford K, Mackenzie C, Milford C (2014). The importance of choice in the rollout of ARV-based prevention to user groups in Kenya and South Africa: a qualitative study. J Int AIDS Soc.

[81] WHO (2012). Guidance on pre-exposure oral prophylaxis (prep) for serodiscordant couples, men and transgender women who have sex with men at high risk of HIV: recommendations for use in the context of demonstration projects.

[82] FDA (2012). FDA approves first drug for reducing the risk of sexually acquired HIV infection.

[83] CDC (2014). Pre exposure prophylaxis for the prevention of HIV infection in the United States/2014. Guidelines.

[84] Molina J-M (2015). On demand PrEP with oral TDF-FTC in MSM: results of the ANRS Ipergay trial.

[85] D'Agostino RB, Ansell BJ, Mora S, Krumholz HM (2014). Clinical decisions. The guidelines battle on starting statins. N Engl J Med.

[86] Mera R, Ng LK, Magnuson D, Campos A, Silva M, Rawling M (2014). Characteristics of Truvada for pre-exposure prophylaxis users in the US (January 2012–September 2013). HIV Drug Therapy in the Americas.

[87] Gomez GB, Borquez A, Case KK, Wheelock A, Vassall A, Hankins C (2013). The cost and impact of scaling up pre-exposure prophylaxis for HIV prevention: a systematic review of cost-effectiveness modelling studies. PLoS Med.

[88] Hellinger FJ (2013). Assessing the cost effectiveness of pre-exposure prophylaxis for HIV prevention in the US. Pharmacoeconomics.

[89] Verguet S, Stalcup M, Walsh JA (2013). Where to deploy pre-exposure prophylaxis (PrEP) in sub-Saharan Africa?. Sex Transm Infect.

[90] Alistar SS, Grant PM, Bendavid E (2014). Comparative effectiveness and cost-effectiveness of antiretroviral therapy and pre-exposure prophylaxis for HIV prevention in South Africa. BMC Med.

[91] Flash C, Landovitz R, Mera Giler R, Ng L, Magnuson D, Bush Wooley S (2014). Two years of Truvada for pre-exposure prophylaxis utilization in the US. Glasgow: International Congress of Drug Therapy in HIV Infection.

[92] (2015). New York City: Department of Health and Mental Hygiene. Health Department Lounches New PrEP and PEP Campaign: new ways to prevent HIV [Press release].

[93] PEPFAR (2014). The U.S. President's emergency plan for AIDS relief, the Bill & Melinda Gates Foundation, and the Nike Foundation Partner on $210 million initiative to reduce new HIV infections in adolescent girls and young women [Internet].

[94] CONSENSUS COMMITTEE (2012). Southern African guidelines for the safe use of pre-exposure prophylaxis in men who have sex with men who are at risk for HIV infection. Southern African Journal of HIV Medicine.

[95] Marcus JL, Glidden DV, Mayer KH, Liu AY, Buchbinder SP, Amico KR (2013). No evidence of sexual risk compensation in the iPrEx trial of daily oral HIV preexposure prophylaxis. PLoS One.

[96] Pines HA, Gorbach PM, Weiss RE, Shoptaw S, Landovitz RJ, Javanbakht M (2014). Sexual risk trajectories among MSM in the United States: implications for pre-exposure prophylaxis delivery. J Acquir Immune Defic Syndr.

[97] Boffito M, Jackson A, Owen A, Becker S (2014). New approaches to antiretroviral drug delivery: challenges and opportunities associated with the use of long-acting injectable agents. Drugs.

[98] McCormack S (2015). Programatic open-label randomised trial of preesposure prophylaxis: the PROUD study.

[99] Tellalian D, Maznavi K, Bredeek UF, Hardy WD (2013). Pre-exposure prophylaxis (PrEP) for HIV infection: results of a survey of HIV healthcare providers evaluating their knowledge, attitudes, and prescribing practices. AIDS Pat Care STDS.

[100] Gengiah TN, Moosa A, Naidoo A, Mansoor LE (2014). Adherence challenges with drugs for pre-exposure prophylaxis to prevent HIV infection. Int J Clin Pharm.

[101] Grant RM (2015). Scale-up of preesposure prophylaxis in San Francisco to impact HIV incidence.

[102] Ware NC, Wyatt MA, Haberer JE, Baeten JM, Kintu A, Psaros C (2012). What's love got to do with it? Explaining adherence to oral antiretroviral pre-exposure prophylaxis for HIV-serodiscordant couples. J Acquir Immune Defic Syndr.

[103] Wade Taylor S, Mayer KH, Elsesser SM, Mimiaga MJ, O'Cleirigh C, Safren SA (2014). Optimizing content for pre-exposure prophylaxis (PrEP) counseling for men who have sex with men: perspectives of PrEP users and high-risk PrEP naive men. AIDS Behav.

[104] Abraham BK, Gulick R (2012). Next-generation oral preexposure prophylaxis: beyond tenofovir. Curr Opin HIV AIDS.

[105] Jespers V, Millwood IY, Poynten IM, Van Damme L, Kaldor JM (2013). The evolving design and methods for trials evaluating the safety of candidate vaginal microbicides: a systematic review. Sex Transm Dis.

[106] Krakower D, Mayer KH (2012). What primary care providers need to know about preexposure prophylaxis for HIV prevention: a narrative review. Ann Intern Med.

[107] Mayer KH (2014). Antiretroviral chemoprophylaxis: state of evidence and the research agenda. Clin Infect Dis.

[108] ClinicalTrials.gov (2014). Safety and effectiveness of tenofovir gel in the prevention of human immunodeficiency virus (HIV-1) infection in women and the effects of tenofovir gel on the incidence of herpes simplex virus (HSV-2) infection US [Internet].

[109] ClinicalTrials.gov (2014). Project ASPIRE efficacy pilot: achieving superior parental involvement for rehabilitative excellence US [Internet].

[110] ClinicalTrials.gov (2014). A phase IIb study to evaluate a long-acting intramuscular regimen for maintenance of virologic suppression (following induction with an oral regimen of GSK1265744 and Abacavir/Lamivudine) in human immunodeficiency virus type 1 (HIV-1) infected, antiretroviral therapy-naive adult subjects US [Internet].

[111] Spreen W, Ford SL, Chen S, Wilfret D, Margolis D, Gould E (2014). GSK1265744 pharmacokinetics in plasma and tissue following single-dose long-acting (LA) injectable administration in healthy subjects. J Acquir Immune Defic Syndr.

[112] Westerhaus M, Castro A (2006). How do intellectual property law and international trade agreements affect access to antiretroviral therapy?. PLoS Med.

[113] Windisch R, Waiswa P, Neuhann F, Scheibe F, de Savigny D (2011). Scaling up antiretroviral therapy in Uganda: using supply chain management to appraise health systems strengthening. Global Health.

# Sub article: Seasonal PrEP for partners of migrant miners in southern Mozambique: a highly focused PrEP intervention

**DOI:** 10.7448/IAS.18.4.19946

**Published:** 2015-07-20

**Authors:** Ide Cremin, Fernando Morales, Britta L Jewell, Kevin R O'Reilly, Timothy B Hallett

**Affiliations:** 1 Department of Infectious Disease Epidemiology, Imperial College London, London, United Kingdom; 2 ICAP Columbia University, New York, NY, USA; 3 World Health Organization, Geneva, Switzerland

**Keywords:** HIV, pre-exposure prophylaxis, ARV-based prevention, cost-effectiveness, mathematical models

## Abstract

**Introduction:**

To be used most effectively, pre-exposure prophylaxis (PrEP) should be prioritized to those at high risk of acquisition and would ideally be aligned with time periods of increased exposure. Identifying such time periods is not always straightforward, however. Gaza Province in southern Mozambique is characterized by high levels of HIV transmission and circular labour migration to mines in South Africa. A strong seasonal pattern in births is observable, reflecting an increase in conception in December. Given the potential for increased HIV transmission between miners returning in December and their partners in Gaza Province, PrEP use by the latter would be a useful means of HIV prevention, especially for couples who wish to conceive.

**Methods:**

A mathematical model was used to represent population-level adult heterosexual HIV transmission in Gaza Province. Increased HIV acquisition among partners of miners in December, coinciding with the miners’ return from South Africa, is represented. In addition to a PrEP intervention, the scale-up of treatment and recent scale-up of male circumcision that have occurred in Gaza are represented.

**Results:**

Providing time-limited PrEP to the partners of migrant miners, as opposed to providing PrEP all year, would improve the cost per infection averted by 7.5-fold. For the cost per infection averted to be below US$3000, at least 85% of PrEP users would need to be good adherers and PrEP would need to be cheaper than US$115 per person per year. Uncertainty regarding incidence of HIV transmission among partners of miners each year in December has a strong influence on estimates of cost per infection averted.

**Conclusions:**

Providing time-limited PrEP to partners of migrant miners in Gaza Province during periods of increased exposure would be a novel strategy for providing PrEP. This strategy would allow for a better prioritized intervention, with the potential to improve the efficiency of a PrEP intervention considerably, as well as providing important reproductive health benefits.

## Introduction

Pre-exposure prophylaxis (PrEP) is a highly effective prevention method, if adhered to [1]. However, PrEP is also expensive to provide. Thus, effective prioritization to those at highest risk of HIV acquisition is essential. A key policy question regarding PrEP is how PrEP should be prioritized and who would be eligible. Much discussion has focused on prioritizing PrEP for key populations such as men who have sex with men, people who inject drugs, sex workers and sero-discordant couples [2, 3], by age [4], and prioritizing within locations with the highest levels of transmission [5, 6]. In addition, PrEP use for an individual would be a short-term method of prevention and therefore should ideally be aligned with specific time periods of high or increased risk of acquisition. However, identifying specific high-risk time periods may not be straightforward for many population groups. Here, we provide a case study of a setting in which a PrEP intervention could potentially be aligned with time periods of increased risk.

Mozambique's HIV epidemic is characterized by strong geographic variation, with the highest levels of prevalence observed in the southern provinces. Population movement, in the form of population displacement, commercial corridors and migrant labour, is thought to have played a considerable role in shaping the epidemiological context and in producing distinct regional epidemics within Mozambique [7]. Since the gold mining industry began in South Africa in the late nineteenth century, there have been high levels of cross-border circular labour migration from the southern provinces of Mozambique (Maputo, Gaza and Inhambane) to mines in South Africa. The earliest Mozambican prevalence data are from the city of Maputo, where HIV prevalence was low and stable until the early 1990s, followed by a rapid increase with prevalence doubling approximately every two years from 1994 onwards [7].

Gaza Province has the highest levels of HIV prevalence in Mozambique. A 2009 AIDS Indicator Survey estimated prevalence to be 30 and 17% among women and men in Gaza Province, respectively [8]. Approximately 40% of Mozambican miners working in South Africa originate from Gaza Province. Within Gaza, the districts of Xai Xai and Chókwè have strong historical ties to mine migration to South Africa, with approximately half of migrant miners from Gaza originating from these two districts [9]. These men typically spend many years working in the South African mines and return to their homes and families in Gaza periodically [9]. A recent survey of men migrating from southern Mozambique to work in South African mines found that, among men from Gaza, almost all have a spouse or long-term partner in Gaza [9]; in addition, casual and commercial sex at the mines is frequent, with 37% of miners reporting at least one “occasional” partner (not a wife or girlfriend) in the last year and 6.6% reporting more than one paid sexual partner in the previous year.

A seasonal pattern of births is observed in Gaza Province, a high-fertility setting, whereby there is a considerable rise in births each September (Figure 1). Increased frequency of conception during December is hypothesized to be related to labour migrants, including miners, returning from South Africa over the Christmas period. In Mozambique, this seasonal trend in institutional births is specific to the southern region where there is a large volume of labour migration to South Africa; it is not observed in northern provinces, where labour migration is infrequent. Given a seasonal trend of an increase in unprotected sex in December, it can be hypothesized that HIV transmission would also increase during this time period. Providing PrEP to partners of migrant miners to coincide with the miners’ return to Gaza for Christmas would allow a well-defined location, population and time for a PrEP intervention. Furthermore, it would provide reproductive health benefits by allowing these women to conceive while also receiving protection from HIV transmission. The aim of this paper is to estimate the prevention impact and the cost-effectiveness of providing time-limited PrEP to partners of migrant miners in Gaza, Mozambique.

**Figure 1:** The reported number of institutional births each month in Gaza Province and Cabo Delgado Province, Mozambique.

## Methods

Following previous work, we used a deterministic population-level model of heterosexual HIV transmission [10]. The model is stratified according to the natural history of HIV infection, sex, male circumcision, three behavioural risk groups and PrEP use. The model is parameterized for Gaza Province and a full model definition is provided in the technical appendix.

Migrant miners and their partners in Gaza are each represented as a behavioural risk group. The proportion of men who are miners is based on the number of men from Gaza who migrate to work in the mines in South Africa, approximately 15,000 men in 2011. This represents 5% of all men in Gaza, and their partners are assumed to represent an equivalent fraction of all women in Gaza. Time-limited transmission is represented such that all transmission between miners and their partners in Gaza is restricted to December each year. Either the miners or their partners can be infected by another partner at any other time throughout the year.

Infection among the miners that occurs in the mining communities in South Africa is represented in the model as a separate incidence term that the miners are subject to for the first 11 months of each year. This incidence is calibrated to produce the observed level of prevalence among migrant miners from Gaza [11, 12].

Self-reported behavioural data indicate that condom use between migrant miners and their regular partners in Mozambique is very infrequent, with the majority (80%) of miners reporting that they had not used a condom at all with their regular partner in Mozambique at any point in the previous year [9, 12]. For simplicity, it is assumed that all sex between miners and their partners in Gaza is condomless and sex acts are frequent throughout December.

The large expansion of antiretroviral therapy (ART) in Gaza Province since 2005 is represented in the model (Supplementary Figure 3). In 2013, there were approximately 43,000 individuals receiving ART, with a larger number of women receiving ART than men. The scale-up of ART as represented in the model corresponds to coverage among all infected individuals, reaching 40% by 2017 and remaining stable at that level beyond 2017. A dropout rate of 5 per 100 person-years (PY) is assumed. ART is assumed to reduce infectiousness by 91%, as reported in a systematic review and meta-analysis of HIV-1 infectiousness per heterosexual partnership based on prospective studies of sero-discordant couples [13].

The recent scale-up of voluntary medical male circumcision in Gaza Province is also represented. Prior to the scale-up of a male circumcision intervention in 2010 [14], the percentage of men that were circumcised is assumed to be low at approximately 20%, as observed for Gaza Province in the 2003 and 2011 Demographic and Health Surveys [15, 16]. The prevalence of male circumcision is assumed to be the same across all adult men in Gaza Province, including the miners. Data regarding the number of men newly circumcised in Gaza Province was used to approximate how the overall prevalence of male circumcision in Gaza would be expected to increase up to 2013. Male circumcision is assumed to reduce the risk of acquisition by 60% [17–19] and have no effect on onward transmission once infected.

The model is calibrated to sex-specific prevalence estimates for Gaza Province from a 2009 AIDS Indicator Survey (Figure 2). A prevalence estimate for miners, from Gaza specifically, was obtained from a recent survey among Mozambican men working in South African mines [11] and was found to be 26% [11]. A prevalence estimate among partners of miners was not available to calibrate the model – an important limitation. Modelled prevalence among partners of miners is 2.6 times lower than prevalence among the miners, due to an assumed lack of exposure throughout the rest of the year. In settings within South Africa, a similarly high differential in prevalence between men migrating long distances to work in the mines and their rural partners has been observed. From the migrant-sending community of Hlabisa in rural KwaZulu-Natal, men who migrated to work in the gold mines in Carletonville, a destination 700 km away, were 3.2 times more likely to be infected as their rural partners, unlike men who migrated from Hlabisa to work in nearby mines, who had similar prevalence to their rural partners [20]. If the partners of miners in Gaza have a true prevalence significantly higher than the modelled estimate, or their patterns of risk behaviour vary substantially, the projected effect of the intervention could be reduced.

**Figure 2:** Modelled prevalence and incidence. Model calibration to prevalence (a); modelled incidence among men and women (b); modelled incidence among partners of miners (c).

The model includes a representation of a PrEP intervention. The efficacy of PrEP per protected sex act is assumed to be 91% [21], with protection dependent on user adherence. PrEP users are split into two groups of adherers – “poor” (20% of users) or “good” (80% of users) – with a different proportion of their sex acts protected. Only uninfected partners of miners receive PrEP. A fixed percentage of partners receive PrEP for the last six weeks of each year. An additional two weeks of PrEP is provided in order to establish sufficient drug concentration to receive protection for the final four weeks of the year. Analyses are presented for a five-year intervention period (2015 to 2020).

The cost of ART is assumed to be US$294 per person per year, based on recent estimates of total per-patient costs for established adult ART patients on first-line regimens in Mozambique [22]. Given that the cost of providing PrEP in this setting is uncertain, a range is explored, with a maximum of US$300 per person per year, as it is assumed that it would not be substantially more expensive to provide PrEP than to provide ART. ART spending is included in the cost calculations to capture the effect of reduced future ART spending due to infections averted by means of PrEP. The net spending is calculated as the difference between intervention spending (PrEP and ART) and baseline spending (ART only). A health system perspective is used to calculate cost per infection averted. All costs are discounted annually by 3%.

## Results

The model calibration to HIV prevalence data is shown in Figure 2a. The large gender differential in prevalence is captured whereby prevalence among women in 2009 is approximately 30%, and prevalence among men in 2009 is much lower, at approximately 17%. Prevalence among the miners is higher at 26% [11]. Prevalence among women from the early 2000s to 2009 is consistent with ANC sentinel surveillance data from Gaza Province, during which time prevalence increased dramatically and subsequently stabilized.

Modelled incidence is higher for women than men (Figure 2b), and spikes in incidence are due to increased transmission between the miners and their partners each December. Incidence among both men and women is projected to decrease gradually and stabilize in the near future. The representation of time-limited transmission between miners and their partners in Gaza in the model produces a spike in the modelled incidence among partners of the miners each December (Figure 2c).

Assuming that PrEP costs US$300 per person per year and that all uninfected women are eligible to receive PrEP, the cost per infection averted is US$15,647 (Figure 3). Providing PrEP specifically to partners of miners increases the cost per infection averted by over fourfold, to US$71,374. This number reflects the relative level of incidence among partners of miners specifically (who have low incidence throughout the year and are assumed to only have elevated incidence for one month each year) as compared to all women in Gaza. However, providing PrEP to partners of the miners for only the last six weeks of the year reduces the cost per infection averted dramatically, to US$9538. Although much less PrEP is being paid for, a similar number of infections are being averted, as the time period of PrEP use corresponds to the time period of increased incidence.

**Figure 3:** The influence of prioritizing PrEP on the estimated cost per infection averted.

Next, a time-limited PrEP intervention for partners of miners is further explored. Adherence determines impact and poor adherence will degrade impact. The level of adherence is unknowable in advance for a myriad of reasons, and thus a wide range is explored. The relationship between adherence and impact, among partners of the miners specifically, is illustrated for several levels of coverage (Figure 4a).

**Figure 4:** The (a) influence of adherence on impact and (b) influence of adherence and cost on cost per infection averted.

Assuming no economies of scale, as is assumed here, the cost per infection averted is constant over coverage. The influence of adherence and cost on cost per infection averted (among the adult population of Gaza Province), assuming 50% coverage, is illustrated in Figure 4b. For the cost per infection averted to be below US$3000, at least 85% of PrEP users would need to be good adherers and PrEP would need to be cheaper than US$115 per person per year.

The true level of HIV incidence in December among partners of migrant miners and the proportion of new infections among partners of the miners that originate from the miners, as opposed to external partners, are unknown. Modelled incidence among partners of the miners is approximately 7 per 100 PY in December, based on the default model parameterization. In the model, the majority (87%) of new infections each year are from the miners (when they return in December) and the remaining 13% are acquired from other partners throughout the year. The true incidence in December is important as it determines the scope of impact of the PrEP intervention. Therefore, analyses are carried out whereby the incidence in December is lower.

The relative impact (as opposed to absolute impact) is similar regardless of the underlying incidence in December (Figure 5, Table 1). As expected, there is an inverse relationship between incidence in the status quo scenario and cost per infection averted. If incidence among partners of miners in December was 4.6 per 100 PY – an incidence rate observed among high-risk women in Chókwè district in Gaza Province [23] – the estimated cost per infection averted would be US$14,868. If incidence among partners of miners in December was much lower at 2 per 100 PY, the estimated cost per infection averted would be as high as US$32,680.

**Figure 5:** The influence of different assumptions regarding the level of transmission occurring between miners and their partners in December.

**Table 1:** The percentage of infections averted among partners of miners and the discounted cost per infection averted for different assumptions regarding the level of transmission occurring between miners and their partners in December.

	(a) 7 per 100 PY (default)	(b) 4.6 per 100 PY (as observed among high risk women in Chókwè	(c) 3 per 100 PY	(d) 2 per 100 PY
Percentage of infections averted among partners of miners (2015–2020)	43.5%	42.0%	39.9%	36.0%
Discounted cost per infection averted ($)	$9,998	$14,868	$21,262	$32,680

Overall, the estimated costs per infection averted would be considerably lower if PrEP was provided for four or five weeks, rather than six, given that modelled incidence is very low in the weeks prior to the miners’ return in December.

## Discussion

Providing PrEP to partners of migrant miners in Gaza Province during periods of increased exposure would be a novel strategy for providing PrEP (i.e. time-limited PrEP), allowing for a better prioritized intervention with the potential to improve the efficiency of a PrEP intervention considerably, minimize potential side effects and provide reproductive health benefits. Key uncertainties include risk behaviour and HIV incidence among men while at the mines, risk behaviour of their partners in Gaza while the miners are away and behaviour in December when the miners return.

Partners of migrant miners in southern Mozambique have been identified as a vulnerable population, given very low condom use and the disproportionately high HIV prevalence among migrant miners [9]. Risk perception among these women is high, with three-quarters of partners of migrant miners surveyed reporting they felt they were at high risk of becoming infected [9]. Absence of condom use has been attributed to lack of power to negotiate condom use within marriage [9]. Fertility intentions among these women may be an additional barrier to condom use. A 2006 survey among rural married women in Gaza Province found that approximately 70% of women wanted to have more children. Furthermore, desire for more children was higher among migrants’ wives compared to non-migrants’ wives, 76.1 vs 64.3%, respectively [23]. Thus, PrEP would be a useful prevention option for women who are at increased risk of HIV acquisition and also wish to conceive.

A number of important simplifications are made in the model. All HIV transmission between the miners and their partners in Gaza is assumed to happen in the month of December each year, whereas in reality some miners return to Gaza more frequently. Previous modelling found that, for a given level of risk behaviour among migrants, the frequency of returning home strongly influenced the risk of acquiring HIV among rural partners, particularly in the early stages of the epidemic [20]. However, this increased risk was outweighed by the influence of increases in risk behaviour due to circular labour migration [20]. In addition, we assume that the partners of miners experience very low incidence for the duration of the time when the miners are away. If the partners of miners have more external partners than assumed, providing time-limited PrEP would not protect them from other sources of infection throughout the year, though this risk would not substantially affect the cost per infection averted. Finally, uninfected partners of miners are assumed to all have the same risk of acquiring HIV. In practice, any effort to further prioritize these women with respect to risk of acquisition would be beneficial. The model was not stratified by age, and prioritizing PrEP for younger women could potentially improve the cost-effectiveness of the intervention.

The strength of the rationale for such a prioritized time-limited PrEP intervention depends on the following: the extent to which there is an increase in births in September; the extent to which this increase can be attributed to the miners’ return at Christmas; and the extent to which an increase in conception around this time leads to an increase in HIV transmission. The data presented reflect institutional births only, which account for approximately 60% of all births. Thus, the true pattern of monthly births might be either more or less pronounced if the remaining 40% of births, that is, home births, were also included. It is unknown how much of the increase in births in September can be attributed to a high frequency of unprotected intercourse following the return of migrant workers at Christmas, as opposed to a behavioural trend unrelated to migration. There are other labour migrant populations who migrate seasonally from Gaza to neighbouring high prevalence countries, for example farm workers, who are likely to be contributing to the observed seasonal pattern of births. However, time-limited PrEP for the partners of miners would still be a useful intervention if the September increase is partially attributable to the miners’ return at Christmas. Many of the miners return to Gaza for two weeks at Easter, yet this return does not manifest in the monthly pattern of births. A possible “Easter effect” would be attenuated by the fact that not all miners return at Easter, as it is a much shorter time period and the timing of Easter varies each year. It would be difficult to quantify the increase in unprotected sex that would be required to produce the increase in births observed in September. Furthermore, quantifying the increase in HIV transmission by translating an increase in unprotected sex would have many uncertainties. Additional data would be required to establish and directly quantify an increase in HIV acquisition specifically among partners of miners during December before implementing such a PrEP intervention.

Given a strong rationale for the proposed PrEP intervention, the feasibility and practicalities of such an intervention need to be addressed. Firstly, the health-care system in rural Mozambique is particularly weak, with less than 40% of the population having access to basic services [9], although there have been improvements in recent years. Secondly, basic HIV prevention services need to be strengthened and expanded. Thirdly, changes in recruitment policies will result in a steady decline in the number of Mozambican men working in the mines in South Africa. Despite this changing situation, a PrEP intervention in Gaza would still be worthwhile because it would protect partners of men who are currently mining. Finally, such a tailored PrEP intervention would need to be carefully executed to avoid the miners and their partners feeling singled out and consequently stigmatized. The acceptability of PrEP for this population and willingness to adhere would ideally be assessed in advance of implementing this intervention. Furthermore, one challenge of implementing such an intervention will be developing a strategy for identifying miners and their partners in Gaza.

Dynamic models of infectious disease transmission have many uses, one of which is as a tool to aid planning and evaluation of interventions [24, 25]. Mathematical models provide a framework to collate several sources of data and make projections of epidemiological impact and cost-effectiveness of an intervention under different sets of assumptions. This analysis provides an example of how mathematical modelling can be useful to inform policy-relevant decision making. Challenges include the availability of data for model calibration and parameterization, as well as uncertainty about patterns of sexual behaviour in the population modelled. Other cost-effectiveness analyses of PrEP have predicted the cost per infection averted to range between cost-saving and US$67,000, depending on the intervention strategy and behavioural characteristics of the population [4]. Providing time-limited PrEP to the partners of miners in Mozambique is more cost-effective than many strategies investigated previously, and it has the potential to become even more cost-effective if the price of PrEP is lower than assumed.

The analyses for this population may be broadly relevant for other migrant groups in different settings, for whom a prioritized intervention such as PrEP would be beneficial. The epidemiological context in Gaza Province provides a novel opportunity for time-limited PrEP, allowing HIV prevention and reproductive health benefits. Beyond a clear need for additional HIV prevention in Gaza Province, seasonal HIV exposure and transmission would provide a niche for a well-prioritized PrEP intervention.

## Supplementary Material

Seasonal PrEP for partners of migrant miners in southern Mozambique: a highly focused PrEP intervention Click here for additional data file.

## References

[1] Baeten JM, Grant R (2013). Use of antiretrovirals for HIV prevention: what do we know and what don't we know?. Curr HIV/AIDS Rep.

[2] Celum C, Baeten JM (2012). Antiretroviral-based HIV-1 prevention: antiretroviral treatment and pre-exposure prophylaxis. Antivir Ther.

[3] Hankins CA (2014). Untangling the cost-effectiveness knot: who is oral antiretroviral HIV pre-exposure prophylaxis really for?. Expert Rev Pharmacoecon Outcomes Res.

[4] Gomez GB, Borquez A, Case KK, Wheelock A, Vassall A, Hankins C (2013). The cost and impact of scaling up pre-exposure prophylaxis for HIV prevention: a systematic review of cost-effectiveness modelling studies. PLoS Med.

[5] Anderson SJ, Cherutich P, Kilonzo N, Cremin I, Fecht D, Kimanga D (2014). Maximising the effect of combination HIV prevention through prioritisation of the people and places in greatest need: a modelling study. Lancet.

[6] Jones A, Cremin I, Abdullah F, Idoko J, Cherutich P, Kilonzo N (2014). Transformation of HIV from pandemic to low-endemic levels: a public health approach to combination prevention. Lancet.

[7] Foreit KF, Barreto AT, Noya PA, Nhatave I (2001). Population movements and the spread of HIV/AIDS in Mozambique. J Health Hum Serv Adm.

[8] Instituto Nacional de Saúde (INS), Instituto Nacional de Estatística (INE), e ICF Macro (2010). Inquérito Nacional de Prevalência, Riscos Comportamentais e Informação sobre o HIV e SIDA em Moçambique 2009.

[9] Crush J, Raimundo I, Simwlane H, Cau B, Dorey D Migration-induced HIV and AIDS in rural Mozambique and Swaziland. Migration Policy Series No. 53.

[10] Cremin I, Alsallaq R, Dybul M, Piot P, Garnett G, Hallett TB (2013). The new role of antiretrovirals in combination HIV prevention: a mathematical modelling analysis. AIDS.

[11] MISAU, INS, CDC, UCSF, MITRAB, I-TECH (2013). Final report: the integrated biological and behavioral survey among Mozambican workers in South African Mines, 2012.

[12] Chemaitelly H, Shelton JD, Hallett TB, Abu-Raddad LJ (2013). Only a fraction of new HIV infections occur within identifiable stable discordant couples in sub-Saharan Africa. AIDS.

[13] Baggaley RF, White RG, Hollingsworth TD, Boily MC (2013). Heterosexual HIV-1 infectiousness and antiretroviral use: systematic review of prospective studies of discordant couples. Epidemiology.

[14] World Health Organization (2012). Progress in scaling up voluntary medical male circumcision for HIV prevention in East and Southern Africa.

[15] Instituto Nacional de Estatística (INE), e IMinisterio da Saude (MISAU), MEASURE DHS+/ORC Macro (2005). Moçambique Inquérito Demográfico e de Saúde 2003.

[16] IMinisterio da Saude (MISAU), Instituto Nacional de Estatística (INE), e ICF International (ICFI) (2013). Moçambique Inquérito Demográfico e de Saúde 2011.

[17] Bailey RC, Moses S, Parker CB, Agot K, Maclean I, Krieger JN (2007). Male circumcision for HIV prevention in young men in Kisumu, Kenya: a randomised controlled trial. Lancet.

[18] Gray RH, Kigozi G, Serwadda D, Makumbi F, Watya S, Krieger JN (2007). Male circumcision for HIV prevention in men in Rakai, Uganda: a randomised trial. Lancet.

[19] Auvert B, Taljaard D, Lagarde E, Sobngwi-Tambekou J, Sitta R, Puren A (2005). Randomized, controlled intervention trial of male circumcision for reduction of HIV infection risk: the ANRS 1265 trial. PLoS Med.

[20] de Bruyn G, Magaret A, Baeten JM, Lingappa JR, Ndase P, Celum C (2012). Mortality in members of HIV-1 serodiscordant couples in Africa and implications for antiretroviral therapy initiation: results of analyses from a multicenter randomized trial. BMC Infect Dis.

[21] Donnell D, Baeten JM, Bumpus NN, Brantley J, Bangsberg DR, Haberer JE (2014). HIV protective efficacy and correlates of tenofovir blood concentrations in a clinical trial of PrEP for HIV prevention. J Acquir Immune Defic Syndr.

[22] Menzies NA, Berruti AA, Blandford JM (2012). The determinants of HIV treatment costs in resource limited settings. PLoS One.

[23] Feldblum PJ, Enosse S, Dube K, Arnaldo P, Muluana C, Banze R (2014). HIV prevalence and incidence in a cohort of women at higher risk for HIV acquisition in Chokwe, southern Mozambique. PLoS One.

[24] Garnett GP, Cousens S, Hallett TB, Steketee R, Walker N (2011). Mathematical models in the evaluation of health programmes. Lancet.

[25] Delva W, Wilson DP, Abu-Raddad L, Gorgens M, Wilson D, Hallett TB (2012). HIV treatment as prevention: principles of good HIV epidemiology modelling for public health decision-making in all modes of prevention and evaluation. PLoS Med.

# Sub article: Cost-effectiveness of pre-exposure prophylaxis targeted to high-risk serodiscordant couples as a bridge to sustained ART use in Kampala, Uganda

**DOI:** 10.7448/IAS.18.4.20013

**Published:** 2015-07-20

**Authors:** Roger Ying, Monisha Sharma, Renee Heffron, Connie L Celum, Jared M Baeten, Elly Katabira, Nulu Bulya, Ruanne V Barnabas

**Affiliations:** 1 Department of Global Health, University of Washington, Seattle, WA, USA; 2 Department of Epidemiology, University of Washington, Seattle, WA, USA; 3 Department of Medicine, University of Washington, Seattle, WA, USA; 4 Infectious Disease Institute, College of Health Sciences, Makerere University, Kampala, Uganda; 5 Vaccine and Infectious Diseases Division, Fred Hutchinson Cancer Research Center, Seattle, WA, USA

**Keywords:** PrEP, mathematical modelling, serodiscordant couples, cost-effectiveness analysis, ART

## Abstract

**Introduction:**

Despite scale-up of antiretroviral therapy (ART) for treating HIV-positive persons, HIV incidence remains elevated among those at high risk such as persons in serodiscordant partnerships. Antiretrovirals taken by HIV-negative persons as pre-exposure prophylaxis (PrEP) has the potential to avert infections in individuals in serodiscordant partnerships. Evaluating the cost-effectiveness of implementing time-limited PrEP as a short-term bridge during the first six months of ART for the HIV-positive partner to prevent HIV transmission compared to increasing ART coverage is crucial to informing policy-makers considering PrEP implementation.

**Methods:**

To estimate the real world delivery costs of PrEP, we conducted micro-costing and time and motion analyses in an open-label prospective study of PrEP and ART delivery targeted to high-risk serodiscordant couples in Uganda (the Partners Demonstration Project). The cost (in USD, in 2012) of PrEP and ART for serodiscordant couples was assessed, with and without research components, in the study setting. Using Ministry of Health costs, the cost of PrEP and ART provision within a government programme was estimated, as was the cost of providing PrEP in addition to ART. We parameterized an HIV transmission model to estimate the health and economic impacts of 1) PrEP and ART targeted to high-risk serodiscordant couples in the context of current ART use and 2) increasing ART coverage to 55% of HIV-positive persons with CD4 ≤500 cells/µL without PrEP. The incremental cost-effectiveness ratios (ICERs) per HIV infection and disability-adjusted life year (DALY) averted were calculated over 10 years.

**Results:**

The annual cost of PrEP and ART delivery for serodiscordant couples was $1058 per couple in the study setting and $453 in the government setting. The portion of the programme cost due to PrEP was $408 and $92 per couple per year in the study and government settings, respectively. Over 10 years, a programme of PrEP and ART for high-risk serodiscordant couples was projected to avert 43% of HIV infections compared to current practice with an ICER of $1340 per infection averted. This was comparable to ART expansion alone, which would avert 37% of infections with an ICER of $1452.

**Conclusions:**

Using Uganda's gross domestic product per capita of $1681 as a threshold, PrEP and ART for high-risk persons have the potential for synergistic action and are cost-effective in preventing HIV infections in high prevalence settings. The annual cost of PrEP in this programme is less than $100 per serodiscordant couple if implemented in public clinics.

## Introduction

Antiretroviral therapy (ART) to treat HIV-positive persons has expanded to almost 10 million patients in low- and middle-income countries in 2013 [1, 2]. The increased coverage has led to significant health gains, such as an (95% confidence interval (CI): 9.6 to 12.9 years) increase in life expectancy by 11.3 years in KwaZulu-Natal, South Africa, from 2003 to 2011 [3]. Furthermore, ecologic data from Kwa Zulu-Natal indicate that ART is associated with substantial decreases in HIV incidence with a 38% (95% CI: 24–50%) reduction in HIV incidence associated with 30–40% ART coverage relative to <10% ART coverage [4]. However, despite the progress in treatment coverage, an additional 15 million HIV-positive persons who are eligible for ART have yet to start [2]. Thus, current coverage provides modest population-level reduction in HIV transmission. Expanding ART coverage may not be straightforward if asymptomatic persons with higher CD4 counts do not initiate ART and achieve durable viral suppression [5, 6]. Primary prevention strategies to prevent HIV acquisition are also needed particularly among high-risk persons. Antiretrovirals provided to HIV-negative persons as pre-exposure prophylaxis (PrEP) reduce the risk of HIV acquisition by up to 75% (95% CI: 55 to 87%) [7–9], and demonstration projects are currently underway to assess the real-world implementation of various PrEP strategies among target populations, including high-risk HIV serodiscordant couples [10, 11].

Populations such as those that participated in several PrEP trials face high annual risks of HIV acquisition [8, 12, 13], and targeting a package of biomedical and behavioural interventions, including PrEP, condoms, and HIV testing and counselling, to these populations may efficiently reduce HIV incidence [14, 15]. HIV serodiscordant couples represent a high-risk population among whom PrEP was shown to be effective [16]. For serodiscordant couples, the greatest period of risk for HIV transmission occurs when the HIV-positive partner is not virally suppressed, including times prior to and soon after initiating ART and during delayed or deferred therapy [5, 6]. During these high-risk periods, PrEP can be an integral component to a combination prevention strategy [17, 18].

Previous analyses found that targeting PrEP to high-risk groups is cost-effective [18–20]. However, these analyses vary widely in their assumptions about the cost of PrEP services [21]. No prior studies have conducted a micro-costing of the programmatic costs of PrEP implementation. To inform health economic analyses and optimize PrEP delivery strategies for HIV serodiscordant couples, micro-costing was conducted to estimate the additional operational costs of PrEP delivery in an open-label, prospective study. These data were then used in a mathematical model of HIV transmission in Kampala, Uganda, to project long-term health and economic outcomes and estimate cost-effectiveness of PrEP implementation.

## Methods

### Study clinic and intervention

The Partners Demonstration Project aims to assess the feasibility of antiretroviral-based interventions (ART and PrEP) to prevent HIV transmission among high-risk serodiscordant couples [22]. Short-term PrEP is used as a “bridge” to prevent transmission prior to viral suppression in the HIV-positive partner during periods where the HIV-positive partner has not yet initiated ART or may not be virally suppressed. Incremental costs were assessed for the programme as implemented at the Kasangati Health Centre – a peri-urban study clinic associated with the Infectious Diseases Institute approximately 15 km north of Kampala, Uganda.

Participants were recruited from local voluntary HIV testing and counselling clinics and community testing campaigns, and screened at Kasangati Health Centre. Eligible couples had HIV-positive partners that were not using ART, HIV-negative partners with normal renal function and a high level of HIV risk (assessed via a validated scoring tool) [23]. Briefly, the risk score is based on the characteristics of the HIV-negative partner (age and, if male, circumcision status) and the partnership (unprotected sex, number of children, and marriage or cohabitation status). Couples who scored ≥5 were considered to be at high risk and were invited to enrol while couples with lower scores were referred for care at a local HIV clinic. Upon enrolment, the HIV-negative partner was offered PrEP (co-formulated emtricitabine/tenofovir disoproxil fumarate (FTC/TDF)), and the couple received a comprehensive HIV prevention package including couples-based HIV prevention counselling and condoms. ART initiation (on site or via referral to a participant's clinic of choice) followed national treatment guidelines (CD4≤350 cells/µL before April 1, 2014; all HIV serodiscordant couples after April 1, 2014), and PrEP use was recommended until the HIV-positive partner had taken ART for at least six months. Couples returned for visits at one and three months after enrolment, and quarterly, thereafter.

### Clinic and participant characteristics

Enrolled individuals (*N*=292 couples) had a median age of 30 years, with over 95% of HIV-negative partners accepting PrEP and over 80% of HIV-positive partners initiating ART [11]. Participant retention was high with >85% of participants completing their expected visits [11] and over 80% of participants taking PrEP medication by blood tenofovir levels [24].

### Cost analysis

Data collection followed the Clinton Health Access Initiative guidelines for costing HIV interventions [25, 26] and the analysis was done from the payer/programmatic (Ministry of Health) perspective. Study budgets, government price lists and personnel interviews were used to estimate the start-up and recurrent costs of the intervention. Costs and activities were divided into three mutually exclusive categories: research, standard of care for couples counseling with ART delivery, and PrEP delivery. Research costs (e.g. completing informed consent) are excluded from this analysis. Standard of care costs and activities were those considered to be normal practice in couples-based HIV counselling and testing, such as sexual behaviour counselling, ART provision and adherence counselling for HIV-positive persons, and viral load monitoring 12 months after ART initiation. The remaining costs were considered PrEP costs (i.e. additional), and are the focus of this analysis. Time and motion studies were conducted to estimate the time needed to counsel participants for PrEP, the number of couples that could be seen annually and the allocation for joint costs. Data collection was done over three weeks from 20 January to 7 February 2014.

All costs and activities were divided into six mutually exclusive resource categories – start-up, personnel, medication, laboratory monitoring, transportation and building and supplies (see Supplementary Table 1). Data for start-up costs were collected from staff interviews and study budgets. Costs included interviewing, hiring and training staff; developing standard operating procedures; and recruiting participants. Personnel costs consisted of annual staff salaries collected from study budgets and weekly trainings that were directly observed (see Supplementary Table 2). Costs for antiretroviral medication (FTC/TDF/efavirenz (EFV) for treatment of HIV-positive partners, and FTC/TDF as PrEP for HIV-negative partners) were collected from drug price lists for the region. Costs of laboratory monitoring by external facilities and rapid diagnostic tests conducted at the health centre were acquired from invoices. Tests delivered to external facilities include CD4 and viral load measurements for the HIV-positive partner and serum creatinine for the HIV-negative partner. Rapid diagnostics conducted on-site include HIV and pregnancy tests. Transportation time and fuel costs incurred for delivering laboratory specimens were collected from interviews with drivers, reviews of driving records and receipts, and direct observation. Costs of buildings and supplies were collected from the market value of equivalent rental spaces and study budgets, respectively. All capital costs (e.g. vehicles and buildings) were annualized over five years with a discount of 3% [27, 28] and inflated to 2012 USD using Ugandan consumer price indices. Joint costs that required allocation were salaries, building, transportation and supplies.

To estimate clinic capacity, health staff were assumed to work an eight-hour workday for five days a week with a one hour break excluding national holidays and paid leave, in line with Ugandan labour guidelines [29] (see Supplementary Table 3). The amount of time per visit type (screening, enrolment, follow-up) was used to estimate the total number of couples the clinic could enrol in 12 months, assuming a screen-to-enrol ratio of 73% and 12-month retention of 97% as observed during the study follow-up period.

### Ministry of health scenario

To estimate the Ministry of Health cost for a PrEP programme, costs and procedures were revised to reflect a programme that would be implemented by the government. First, private-sector salaries were replaced with public-sector salaries using 2009 estimates adjusted to 2012 using the ratio of Uganda's consumer price indices in 2012 and 2009 [30]. Second, the annual cost of PrEP medication (FTC/TDF) was reduced from current Ugandan private-sector list price ($382) to the lowest estimate as negotiated by the Clinton Health Access Initiative ($75) [31]. Third, viral load tests were assumed to occur only at Month 12 for clinical monitoring, with laboratory tests for viral load and HBV screening replaced with point-of-care tests ($20 and $0.50, respectively) [32, 33]. Finally, a previously validated model of task-shifting used by Médecins Sans Frontières that allowed nurses to prescribe ART, used adherence counsellors and organized HIV support groups [34] was used to estimate the impact of task-shifting on clinic capacity. The task-shifting programme did not impair treatment outcomes and improved ART adherence.

### Cost-effectiveness analysis

The estimated Ministry of Health incremental cost for a PrEP programme was incorporated into a dynamic transmission model of HIV [35] that was parameterized to southwest Uganda using estimates from the literature and a study of home HIV testing and counselling in southwest Uganda [36]. The model is stratified by age, gender and sexual activity, and includes HIV stage by CD4 T-cell count and HIV RNA viral load, and was calibrated to fit HIV incidence and prevalence from the region. Further details can be found in Supplementary Table 4 and Supplementary file 2. The model was used to estimate the cost-effectiveness of 1) implementing a PrEP and ART programme for high-risk serodiscordant couple, or 2) scaling-up ART to the new ART initiation guidelines (CD4≤500 cells/µL). The incremental cost-effectiveness ratios (ICERs) were calculated for each scenario.

The first scenario simulated current ART coverage in Uganda of 40% among all HIV-positive persons, with 60% coverage for persons with CD4 ≤200 cells/µL, 50% for persons with CD4 200–350 cells/µL and 10% for persons with CD4 350–500 cells/µL. The second scenario simulated increased ART coverage for persons with CD4 ≤500 cells/µL as the recently changed ART eligibility criteria are implemented, such that ART coverage for persons with CD4 350–500 cells/µL is 50%, assuming the same coverage of ART as seen with previous ART initiation guidelines. The third scenario simulated PrEP and ART targeted to 90% of high-risk serodiscordant couples. In the model, high-risk serodiscordant couples are defined as those partnerships in which the HIV-negative partner is aged <25 years and belongs to the high sexual activity group (i.e. the top 15th percentile in the number of casual sex partners). Annual drop-out rates from ART and PrEP were assumed to be 6% [37].

To estimate the health and economic impact of the modelled scenarios, costs of PrEP delivery, ART [25] and hospitalization [38] were used to calculate the ICER per HIV infection averted and disability-adjusted life year (DALY) averted for each scenario (ART scale-up or PrEP and ART for high-risk serodiscordant couples). The ICER was calculated using outcomes from a 10-year time horizon, with costs and effectiveness measures discounted by 3% annually. Consistent with health-economic conventions [27], we regard an intervention as very cost-effective if the cost per DALY or HIV infection averted is less than Uganda's per capita gross domestic product (GDP) in 2012 ($1681) [39], and cost-effective if the cost per DALY averted is less than three times Uganda's per capita GDP ($5043).

### Sensitivity analyses

To explore how the ICER of the PrEP programme changes with different programmatic assumptions, sensitivity analyses were conducted. First, the clinic capacity was varied from 200 to 1500 couples retained for 12 months, assuming a constant number of staff and 97% patient retention over 12 months. Second, the cost of PrEP delivery based on clinic capacity (200 to 1500 couples retained at Month 12), efficacies of ART and PrEP for reducing HIV transmission (73 to 99% [40] and 77 to 98% [41], respectively), drop-out rate from ART and PrEP (0 to 10%), annual discount rate (0 to 10%) and ART cost ($100 to $500 per person per year) were all varied independently to estimate the sensitivity of the ICER.

## Results

### Overall and PrEP intervention costs

Seven screening visits, five enrolment visits, and eighteen follow-up visits were observed. The average total visit times including research components for the screening, enrolment and follow-up visits were 2.7, 3.8 and 1.3 hours, respectively. As studied, the cost of PrEP components, incremental to the cost of ART for serodiscordant couples, was $408 annually per couple. In the best-case scenario using Ministry of Health prices, the incremental cost of PrEP components decreased to $92 annually per couple.

### “As Studied” scenario

In the “As Studied” estimate of $408 annually per couple, we assumed that four nurse counsellors and two clinicians provided care in a clinic that could screen 1086 couples per year, enrol 793 (73%) of them and retain 769 (97%) by Month 12 (Table 1). This programme would cost $827,351 ($1058 per couple retained for 12 months), with the majority of the costs going towards medication and laboratory monitoring (54 and 26%, respectively). Of the total cost, 44% ($363,012) was attributable to the PrEP programme and 56% ($464,340) to standard of care. Considering only the additional costs of PrEP (Figure 1a), PrEP-related laboratory monitoring costs contribute 46% of additional PrEP intervention costs, whereas PrEP medication costs contribute 37% of additional PrEP intervention costs.

**Table 1:** Comparison of outcomes excluding research components

		Time per visit (hours)				
				
Scenario		Screening	Enrolment	Follow-up	No. of couples at Month 12	Cost per couple
As studied^a^	Total clinical^c^	1.5	2.5	1.1	769	$1058
	PrEP	0.6	1.3	0.6		$408
Ministry of Health^b^	Total clinical^c^	1.4	1.5	0.7	1111	$453
	PrEP	0.4	0.6	0.4		$92

The time per visit was estimated from time and motion observations at the clinic

a outcomes as observed in the Partners Demonstration Project

b assumes public-sector salaries, point-of-care laboratory tests, less expensive medication and task-shifting

c includes standard care and PrEP components.

**Figure 1:** Additional PrEP programme costs by resource type. The allocation of costs by resource type for the intervention “As Studied” (a) and in the “Ministry of Health” (b). “Ministry of Health” includes public-sector salaries, fewer laboratory tests, less expensive medication and task-shifting.

### Ministry of health scenario

The Ministry of Health scenario assumes that PrEP delivery by the government would cost less than as implemented in the study as salaries and medication costs would be lower than those in the research setting. In addition, less laboratory monitoring would be conducted, according to national guidelines (Table 2).

**Table 2:** Change in costs with additional assumptions

Programme change	Number of couples	Total cost per couple	ART only cost per couple	Additional PrEP cost per couple
Baseline (No PrEP)	769	$650	$650	$0
As Studied with PrEP	769	$1058	$650	$408
With public-sector staff salaries	769	$1005	$635	$370
With reduced medication cost	769	$720	$466	$254
With fewer laboratory tests^a^	769	$497	$396	$101
With task-shifting	1111	$453	$361	$92

The impact of programmatic changes on the capacity of a PrEP programme and the annual cost per couple retained for one year

a simplified testing with one point-of-care HBV test and one point-of-care viral load measurement.

With public-sector salaries, the additional cost per couple decreased from $408 to $370 annually. Using the annual per-person cost of PrEP (FTC/TDF) negotiated by the Clinton Health Access Initiative ($75) [31], the annual additional cost of intervention per couple decreased from $370 to $254, and reduced the medication portion of the total cost from 41 to 15%. Using one point-of-care viral load test conducted at 12 months to monitor clinical response to ART and one point-of-care HBV test to screen HIV-negative partners, the annual cost per couple decreased from $254 to $101. Finally, task-shifting of clinical activities resulted in screening, enrolment and follow-up visit times of 1.4, 1.5 and 0.7 hours, respectively (Table 1), and increased clinic capacity to 1111 couples retained at Month 12. As a result, the annual additional cost per couple decreases to $92. In the Ministry of Health scenario, the proportion of costs due to laboratory monitoring decreased from 46% in the study to 37%, whereas the proportion of costs due to medication increased from 37% in the study to 41% (Figure 1b).

### Cost-effectiveness analysis

In the cost-effectiveness analysis (Table 3), targeting PrEP and ART to high-risk serodiscordant couples averts 43% more HIV infections than baseline and is cost-effective with an ICER of $1340 per HIV infection averted over 10 years, whereas ART scale-up alone averts 37% more HIV infections than baseline, costing $1452 per incident HIV case averted relative to baseline.

**Table 3:** Incremental cost-effectiveness ratios (ICERs) of ART and PrEP strategies for southwest Uganda

Outcome	Scenario	Effectiveness	Cost (millions USD)	ICER
HIV infections averted	Baseline: Current ART uptake ART: Baseline (40%)^a^ PrEP: N/A	94,000	185	Baseline
	ART scale up only (no PrEP) ART: CD4≤500 cells/µL (55%) PrEP: N/A	104,000 (37%)	200	Dominated^b^
	MoH adds PrEP programme for all high-risk serodiscordant couples ART: Baseline (40%)^a^+high-risk couples^c^ without CD4/VL criteria (80%) PrEP: High-risk couples^c^ (80%)	120,000 (43%)	219	$1340
DALYs averted	Baseline: Current ART uptake ART: Baseline (40%)^a^ PrEP: N/A	203,000	185	Baseline
	ART scale up only (no PrEP) ART: CD4≤500 cells/µL (55%) PrEP: N/A	217,000 (60%)	200	$1075
	MoH adds PrEP programme for all high-risk serodiscordant couples ART: Baseline (40%)^a^+high-risk SDC^c^ without CD4/VL criteria (80%) PrEP: High-risk couples^c^ (80%)	221,000 (62%)	219	$5354

Results are shown for a 10-year time horizon relative to 2014

a under former guidelines

b extended dominance occurs when a strategy is less cost-effective than a combination of other strategies

c high-risk serodiscordant couples are those in which the HIV-negative partner is ≤25 years old and both partners are in the top 15th percentile in the number of casual sexual partners.

When considering the outcome of DALYs averted, the ICER for PrEP and ART together is higher than for ART scale-up alone. It is the most effective strategy, averting 62% more DALYs than baseline, but the ICER of $5354 per DALY averted is slightly higher than three times Uganda's GDP per capita ($5043), the threshold for cost-effectiveness. In this case, scaling up ART only was the most cost-effective strategy at $1075 per DALY averted while averting 60% more DALYs than baseline.

### Sensitivity analysis

Increasing the clinic capacity from 200 to 1500 couples annually in the primary cost estimates from Table 1 decreased the additional cost per couple in the PrEP programme at Month 12 from $254 to $82 (Figure 2), suggesting that the incremental cost can increase substantially if clinic capacity is very low.

**Figure 2:** Annual incremental cost per couple by annual number of couples enrolled. The costs are based on the Ministry of Health scenario with public-sector salaries, fewer laboratory tests, less expensive medication and task-shifting. The clinic capacity assumes a screen-to-enrol ratio of 1.37, and 97% retention of enrolled couples over 12 months.

In sensitivity analyses, a high clinic capacity (1500 couples annually, costing $82 per couple) reduced the ICER of the PrEP programme to $4648 per DALY averted, whereas low clinic capacity (200 couples annually, costing $254 per couple) increased the ICER to $18,151 per DALY averted. Similarly, the cost per HIV infection averted increases dramatically with decreased clinic capacity. With ART cost at $100 per person per year, no annual discounting and 10% drop-out from ART and PrEP, the PrEP programme becomes cost-effective for averting DALYs, although the programme never becomes very cost-effective for averting DALYs. For averting HIV infections, PrEP remains the most cost-effective strategy across all ranges of assumptions. It is consistently very cost-effective (i.e. less than Uganda's per capita GDP) except when assuming low per person annual ART cost ($100), in which the ICER per infection averted is $521 for ART scale-up and $1515 for the PrEP programme (Figure 3).

**Figure 3:** Sensitivity of the ICER per DALY (a) and HIV infection (b) averted for the high-risk serodiscordant couples PrEP programme. The base case ICER is $5354 per DALY averted and $1340 per HIV infection averted.

## Discussion

This comprehensive micro-costing of implementing PrEP as a “bridge” among high-risk serodiscordant couples until ART initiation and viral suppression by the HIV-positive partner was used to inform a dynamic simulation model of HIV transmission. Adding a PrEP bridging intervention until ART initiation to standard of care by WHO and Ugandan guidelines is very cost-effective for averting HIV infections in generalized HIV epidemic settings, such as Kampala, Uganda. The average private sector “as studied” clinical cost per couple retained after 12 months was $1058, with about half of the cost ($408) being due to the PrEP intervention. The majority (83%) of the PrEP costs were attributable to laboratory monitoring and PrEP medication (FTC/TDF). If this programme were implemented by the Ministry of Health, government salaries, reduced drug costs, fewer laboratory tests and task-shifting could reduce cost and increase efficiency, resulting in a PrEP intervention cost of less than $100 per couple per year. Comparing the ICERs for a PrEP and ART programme targeted to serodiscordant couples, and expanding ART coverage relative to current practice, could guide expansion of HIV prevention programmes. For averting HIV infections, implementing a PrEP and ART programme for high-risk serodiscordant couples is very cost-effective, and increasing ART coverage to 55% of HIV-positive persons with CD4≤500 cells/µL without PrEP is not cost-effective. When the outcome considered was DALYs averted, PrEP and ART together averted the most DALYs but slightly exceeded the cost-effectiveness threshold of three times Uganda's GDP per capita. Instead, increasing ART coverage is the most cost-effective strategy. These results are explained by PrEP being an HIV prevention intervention, and the majority of the intervention's impact on averting DALYs due to HIV not being captured within the 10-year time horizon of the analysis. ART treatment alone, in contrast, has an immediate effect on averting DALYs, particularly for those at lower CD4 counts.

Reaching efficient PrEP implementation will require clearly defined strategies for intervention delivery. The WHO recently published guidelines that recommended targeting PrEP to serodiscordant couples, and men and transgender women who have sex with men [42] and added that demonstration projects are necessary to develop reasonable frameworks for delivering PrEP. Initial studies of the impact of PrEP as a bridge to ART among serodiscordant couples estimate a 96% reduction in HIV incidence [11]. Further studies are needed to verify the assumptions made to achieve efficient scenarios that maximize demand and clinic capacity. Efficient scale-up will depend on increased patient and provider knowledge of PrEP, as well as increased accessibility of PrEP drugs with couples counselling. Finally, task-shifting has been piloted in several regions in sub-Saharan Africa and results show that shifting clinical responsibilities from physicians to other staff does not necessarily affect clinical outcomes [43] and, in some cases, may improve them [44]. Although we estimated that task-shifting reduced the amount of time per clinic visit compared to the demonstration project by more efficiently using staff skills, the overall impact on cost per couple is small.

This analysis has several limitations. We assume that there is sufficient demand for PrEP services such that the clinic is at full capacity. Staff likely have other health care tasks unrelated to PrEP delivery, but data to quantify this were not available. In addition, the act of observing counselling sessions may influence the counselling interaction. However, clinic staff were informed that observations were related to a costing analysis and not a staff evaluation, and multiple staff were observed over the three-week period to ensure robustness of the data. Moreover, study staff had more training and experience in couples counselling and PrEP provision than is typical at public health clinics. However, the sensitivity analysis suggests that even if fewer couples were retained at Month 12 and the screening-to-enrolment ratio were higher, the cost per couple would not change substantially, though the ICER would increase dramatically at very low clinic capacities. Our model also makes assumptions regarding ART uptake under new guidelines (CD4≤500 cells/µL), which we conservatively assume as 55% of all HIV-positive persons achieving viral suppression, assuming ART uptake among asymptomatic persons is the same as was seen among symptomatic individuals. While this assumption is realistic in the short term, it favourably impacts our assessment of PrEP cost-effectiveness. Increasing the coverage and adherence to ART would result in a lower cost-effectiveness per HIV infection averted for a combined PrEP and ART programme. However, data on ART adherence under the new guidelines are insufficient.

To our knowledge, these are the first primary cost estimates for PrEP counselling and provision in Africa. Previous studies have used lower cost estimates for HIV testing and counselling than found here in the Partners Demonstration Project, leading their estimates to be between our “as studied” and “Ministry of Health” scenarios [45–47]. Previous modelling studies of PrEP focusing on South Africa have found PrEP to cost less than two times the per capita GDP per HIV infection averted [21], similar to our estimate, but the estimates are not directly comparable due to differences in the HIV epidemics.

## Conclusions

ART coverage in sub-Saharan Africa has been rapid and successful, but only approximately one-third of HIV-positive persons are virally suppressed. Additional interventions are needed to give individuals at high risk of HIV acquisition a method for protecting themselves. PrEP can serve as a short-term primary prevention strategy during periods of high risk [48]. This analysis suggests that incorporating PrEP into existing HIV testing and counselling and ART programmes is a cost-effective method for HIV prevention.

## Supplementary Material

Cost-effectiveness of pre-exposure prophylaxis targeted to high-risk serodiscordant couples as a bridge to sustained ART use in Kampala, Uganda Click here for additional data file.

Cost-effectiveness of pre-exposure prophylaxis targeted to high-risk serodiscordant couples as a bridge to sustained ART use in Kampala, Uganda Click here for additional data file.

## References

[1] UNAIDS/WHO (2013). Global report: UNAIDS report on the global AIDS epidemic, 2013.

[2] UNAIDS (2014). Fast-track: ending the AIDS epidemic by 2030.

[3] Bor J, Herbst AJ, Newell ML, Bärnighausen T (2013). Increases in adult life expectancy in rural South Africa: valuing the scale-up of HIV treatment. Science.

[4] Tanser F, Barnighausen T, Grapsa E, Zaidi J, Newell ML (2013). High coverage of ART associated with decline in risk of HIV acquisition in rural KwaZulu-Natal, South Africa. Science.

[5] Katz IT, Essien T, Marinda ET, Gray GE, Bangsberg DR, Martinson NA (2011). Antiretroviral therapy refusal among newly diagnosed HIV-infected adults. AIDS.

[6] Mujugira A, Celum C, Thomas KK, Farquhar C, Mugo N, Katabira E (2014). Delay of antiretroviral therapy initiation is common in East African HIV-infected individuals in serodiscordant partnerships. J Acquir Immune Defic Syndr.

[7] Grant RM, Lama JR, Anderson PL, McMahan V, Liu AY, Vargas L (2010). Preexposure chemoprophylaxis for HIV prevention in men who have sex with men. N Engl J Med.

[8] Abdool Karim Q, Abdool Karim SS, Frohlich JA, Grobler AC, Baxter C, Mansoor LE (2010). Effectiveness and safety of tenofovir gel, an antiretroviral microbicide, for the prevention of HIV infection in women. Science.

[9] Choopanya K, Martin M, Suntharasamai P, Sangkum U, Mock PA, Leethochawalit M (2013). Antiretroviral prophylaxis for HIV infection in injecting drug users in Bangkok, Thailand (the Bangkok Tenofovir Study): a randomised, double-blind, placebo-controlled phase 3 trial. Lancet.

[10] Mugo NR (2014). Notes from the field: implementing PrEP in resource constrained settings.

[11] Baeten J (2015). Near elimination of HIV transmission in a demonstration project of PrEP and ART.

[12] Van Damme L, Corneli A, Ahmed K, Agot K, Lombaard J, Kapiga S (2012). Preexposure prophylaxis for HIV infection among African women. N Engl J Med.

[13] Marrazzo J, Ramjee G, Nair G, Palanee T, Mkhize B, Nakabiito C (2013). Pre-exposure prophylaxis for HIV in Women: daily oral tenofovir, oral tenofovir/emtricitabine, or vaginal tenofovir gel in the VOICE Study (MTN 003).

[14] Ying R, Celum C, Baeten J, Murnane P, Hong T, Krows M (2014). Pre-exposure prophylaxis for young women with concomitant partner testing can cost-effectively reduce HIV incidence in KwaZulu-Natal, South Africa.

[15] Anderson SJ, Cherutich P, Kilonzo N, Cremin I, Fecht D, Kimanga D (2014). Maximising the effect of combination HIV prevention through prioritisation of the people and places in greatest need: a modelling study. Lancet.

[16] Baeten JM, Donnell D, Ndase P, Mugo NR, Campbell JD, Wangisi J (2012). Antiretroviral prophylaxis for HIV prevention in heterosexual men and women. N Engl J Med.

[17] Celum C, Baeten JM, Hughes JP, Barnabas R, Liu A, Van Rooyen H (2013). Integrated strategies for combination HIV prevention: principles and examples for men who have sex with men in the Americas and heterosexual African populations. J Acquir Immune Defic Syndr.

[18] Hallett TB, Baeten JM, Heffron R, Barnabas R, de Bruyn G, Cremin Í (2011). Optimal uses of antiretrovirals for prevention in HIV-1 serodiscordant heterosexual couples in South Africa: a modelling study. PLoS Med.

[19] Verguet S, Stalcup M, Walsh JA (2013). Where to deploy pre-exposure prophylaxis (PrEP) in sub-Saharan Africa?. Sex Transm Infect.

[20] Walensky RP, Park JE, Wood R, Freedberg KA, Scott CA, Bekker LG (2012). The cost-effectiveness of pre-exposure prophylaxis for HIV infection in South African women. Clin Infect Dis.

[21] Gomez GB, Borquez A, Case KK, Wheelock A, Vassall A, Hankins C (2013). The cost and impact of scaling up pre-exposure prophylaxis for HIV prevention: a systematic review of cost-effectiveness modelling studies. PLoS Med.

[22] Heffron R, Celum C, Mugo N, Katabira E, Bukusi E, Asiimwe S (2014). High initiation and adherence among high risk African HIV discordant couples in a demonstration project of PrEP and ART for HIV prevention.

[23] Kahle EM, Hughes JP, Lingappa JR, John-Stewart G, Celum C, Nakku-Joloba E (2013). An empiric risk scoring tool for identifying high-risk heterosexual HIV-1-serodiscordant couples for targeted HIV-1 prevention. J Acquir Immune Defic Syndr.

[24] Heffron R, Ngure K, Semiyaga NB, Odoyo J, Tindimwebwa E, Morton J (2015). Sustained PrEP use among high-risk African HIV serodiscordant couples in a PrEP demonstration project.

[25] Clinton Health Access Initiative (2011). Facility-based unit costing for antiretroviral treatment in five sub-Saharan African countries.

[26] UNAIDS (2000). Costing guidelines for HIV prevention strategies.

[27] Gold MR (1996). Cost-effectiveness in health and medicine.

[28] Drummond MF (1997). Methods for the economic evaluation of health care programmes.

[29] The Employment Act (2006). Edited by Ministry of Gender L, and Social Development.

[30] Africa Health Workforce Observatory (2009). Human resources for health: country profile, Uganda.

[31] Clinton Health Access Initiative (2013). Antiretroviral (ARV) ceiling price list.

[32] Lee HH, Dineva MA, Chua YL, Ritchie AV, Ushiro-Lumb I, Wisniewski CA (2010). Simple amplification-based assay: a nucleic acid-based point-of-care platform for HIV-1 testing. J Infect Dis.

[33] Gish RG, Gutierrez JA, Navarro-Cazarez N, Giang K, Adler D, Tran B (2014). A simple and inexpensive point-of-care test for hepatitis B surface antigen detection: serological and molecular evaluation. J Viral Hepat.

[34] Bedelu M, Ford N, Hilderbrand K, Reuter H (2007). Implementing antiretroviral therapy in rural communities: the Lusikisiki model of decentralized HIV/AIDS care. J Infect Dis.

[35] Barnabas R, Ying R, van Rooyen H, Murnane P, Hughes J, Baeten J (2013). Use of HIV viral-load suppression to estimate the effect of community-wide home-based HIV counselling and testing and linkage to antiretroviral therapy on HIV incidence in South Africa: a mathematical modelling analysis. Lancet.

[36] Barnabas RV, Van Rooyen H, Baeten J, Tumwesigye E, Phakathi Z, Tumwebaze H (2012). High testing uptake and linkages to HIV treatment through home-based HIV counseling and testing and facilitated referral in Kabwohe, Uganda and KwaZulu-Natal (KZN), South Africa. Treatment as prevention.

[37] National AIDS and STI Control Programme (NASCOP), Kenya (2014). Kenya AIDS Indicator Survey 2012: Final Report.

[38] Meyer-Rath G, Brennan AT, Fox MP, Modisenyane T, Tshabangu N, Mohapi L (2013). Rates and cost of hospitalization before and after initiation of antiretroviral therapy in urban and rural settings in South Africa. J Acquir Immune Defic Syndr.

[39] International Monetary Fund (2012). World economic outlook database.

[40] Cohen MS, Chen YQ, McCauley M, Gamble T, Hosseinipour MC, Kumarasamy N (2011). Prevention of HIV-1 infection with early antiretroviral therapy. N Engl J Med.

[41] Baeten JM, Donnell D, Mugo NR, Ndase P, Thomas KK, Campbell JD (2014). Single-agent tenofovir versus combination emtricitabine plus tenofovir for pre-exposure prophylaxis for HIV-1 acquisition: an update of data from a randomised, double-blind, phase 3 trial. Lancet Infect Dis.

[42] World Health Organization (2012). Guidance on Pre-exposure oral prophylaxis (PrEP) for serodiscordant couples, men and transgender women who have sex with men at high risk of HIV: recommendations for use in the context of demonstration projects.

[43] Callaghan M, Ford N, Schneider H (2010). A systematic review of task- shifting for HIV treatment and care in Africa. Hum Resour Health.

[44] Campion EW (2015). Treating millions for HIV – the adherence clubs of Khayelitsha. N Engl J Med.

[45] Larson BA, Bii M, Henly-Thomas S, McCoy K, Sawe F, Shaffer D (2013). ART treatment costs and retention in care in Kenya: a cohort study in three rural outpatient clinics. J Int AIDS Soc.

[46] Menzies NA, Berruti AA, Berzon R, Filler S, Ferris R, Ellerbrock TV (2011). The cost of providing comprehensive HIV treatment in PEPFAR-supported programs. AIDS.

[47] Bill & Melinda Gates Foundation (2011). Oral PrEP in South Africa. Bottom-up cost model.

[48] Pines HA, Gorbach PM, Weiss RE, Shoptaw S, Landovitz RJ, Javanbakht M (2014). Sexual risk trajectories among MSM in the United States: implications for pre-exposure prophylaxis delivery. J Acquir Immune Defic Syndr.

# Sub article: Beyond “getting drugs into bodies”: social science perspectives on pre-exposure prophylaxis for HIV

**DOI:** 10.7448/IAS.18.4.19983

**Published:** 2015-07-20

**Authors:** Judith D Auerbach, Trevor A Hoppe

**Affiliations:** 1 Center for AIDS Prevention Studies, Department of Medicine, School of Medicine, University of California, San Francisco, CA, USA; 2 Department of Criminology, Law & Society, University of California, Irvine, Irvine, CA, USA

**Keywords:** PrEP, social science, HIV prevention, sexuality, risk compensation

## Abstract

Social scientists have much to contribute to the analysis of the real and potential contribution of pre-exposure prophylaxis (PrEP) to HIV prevention around the world. Beyond just a matter of clinical efficacy and getting pills into people's mouths, PrEP raises a number of important social-psychological questions that must be attended to in order to translate biomedical and clinical findings into uptake of PrEP among enough people at risk of HIV infection to produce population-level effectiveness. PrEP is a dynamic phenomenon with “dialectical” attributes that invite both optimism and cynicism as a desirable and effective HIV prevention strategy. PrEP disrupts traditional notions of “safe” and “unsafe” sex; it confers on its users a level of agency and control not generally achieved with condoms; and it affects sexual practices and sexual cultures in meaningful ways. As these dynamics play out in different contexts, and as new modes of PrEP administration emerge, it will be important for social scientists to be engaged in assessing their impact on PrEP implementation and effectiveness.

## Introduction

Among the HIV biomedical, clinical and advocacy communities, discussions of pre-exposure prophylaxis (PrEP) have largely focused on two questions: Is it clinically effective? and What are the structural and policy factors that impact its effectiveness when implemented? But, PrEP raises a number of other important social-psychological questions that also must be attended to in order to translate biomedical and clinical findings into uptake of PrEP among enough people at risk of HIV infection to produce population-level effectiveness. Social science has much to contribute in this regard.

Social scientists take as their starting point that PrEP is a dynamic phenomenon that is more than just a pharmacologic intervention – that is, getting PrEP to “work” is more complicated than simply “getting drugs into bodies.” Rather, PrEP embodies a range of interacting physiological, psychological and social realities that together affect not only an individual's risk or avoidance of HIV infection but also relationship dynamics, sexual cultures and social arrangements that have influence beyond HIV. We explore some of this dynamism and the issues it raises for further understanding of the role of PrEP in HIV prevention and to make the case that social science perspectives are essential as further implementation of PrEP ensues.

## The dynamic nature of PrEP

PrEP emerges in, and itself effects, a dynamic situation. In the context of combination HIV prevention, PrEP provides another method in the ever-evolving constellation (or “toolbox”) of evidence-based prevention strategies. It enhances the repertoire of choices individuals can make about how best to protect themselves from acquiring HIV, taking into account the realities of one's life, the nature of one's sexual and drug-using practices and relationships, and personal preferences about behavioural and technological “interventions.”

Perhaps most importantly, PrEP's demonstrated efficacy among gay and other men who have sex with men (MSM) and transgender women [1], heterosexual men and women [2, 3], and men and women who inject drugs [4] disrupt traditional notions of “protected” and “unprotected” sex, and of “risky” and “safe” sex and drug use [5–7] – notions that have been institutionalized in public health and community (especially gay community) discourse and practice for the past three decades. (As a case in point, PrEP's disruptive effect, alongside other advances in using antiretroviral (ARV) drugs for HIV prevention, recently helped to spur the U.S. Centers for Disease Control and Prevention (CDC) to stop using the term “unprotected sex” to refer to sexual intercourse without a condom [8].)

The promotion of ARV-based prevention approaches – whether “treatment as prevention,” “pre-exposure prophylaxis” or “post-exposure prophylaxis” – imbues the person taking ARV with a responsibility to care for his/her health as well as that of others [9]. Throughout the course of the HIV epidemic, the collective responsibility for preventing HIV has shifted from the promotion of condom use by HIV-negative persons to recommending that HIV-positive individuals begin taking ARV early in the course of their infection. Efforts to promote PrEP could again shift the responsibility for prevention back towards HIV-negative individuals [10], underscoring the dynamic ways in which individuals (and couples) interact with drugs and the drugs change their realities.

## The dialectics of PrEP

When PrEP first emerged on the HIV prevention landscape, much of the popular discourse surrounding it was framed in a binary and oppositional fashion, that is, either PrEP holds the promise to ending the HIV pandemic or PrEP is an insidious strategy that will exacerbate HIV epidemics and attendant social ills [11]. Many social scientists argue instead that PrEP as a technology is not inherently “good” or “bad” – it has both positive and negative potentialities simultaneously and produces something new entirely as a result of the dynamic tension between them [6]^.^ This dialectic can be seen in a number of areas explored below.

### Efficacy

Evidence from key clinical trials has shown that, if taken daily as prescribed, oral Truvada for PrEP is a highly efficacious HIV prevention strategy. It may reduce HIV acquisition by more than 90%, placing it right next to male latex condoms and access to sterile syringes as the most efficacious HIV prevention methods available today. But, data from the same clinical trials and others [12] indicate that most participants did not take oral PrEP as prescribed. Although adherence observed in clinical trials is likely to vary considerably from levels in the “real world,” trial results suggest that implementation programmes may need to greatly increase adherence levels in order to maximize the likelihood that PrEP will have a population-level impact.

Furthermore, although treatment-resistant mutations have not been witnessed in clinical trials as of yet, there is some concern that low adherence levels create the potential in individuals with partial adherence to develop resistance to certain classes of ARV, should they become HIV infected (and to transmit those strains onward). However, especially in light of recent data suggesting that even intermittent use of PrEP can be highly effective at preventing infections [13], evidence to date does not support this argument.

### Agency and control

PrEP also has the potential to confer agency and control on HIV-uninfected persons who heretofore have had to depend on willingness of partners to use condoms or ARV as their primary prevention strategies [14, 15]. The possibility of using PrEP without the knowledge of the other partner is a very important development for anyone who needs an HIV prevention method that can be used surreptitiously, as has been argued for microbicides, particularly for women [16, 17].

Relatedly, PrEP in its current form as a once-daily pill is not coitally dependent, so individuals can take it at any time during the day they wish and not have it interrupt or interfere with any particular sexual episode (i.e., before, during or after intercourse). This unobtrusiveness, in combination with the relief of knowing one is protected from HIV, confers some level of control on the PrEP user. It also imbues PrEP with the potential to enhance sexual pleasure and fulfilment [18]. This should continue to be the case when other methods of PrEP administration (such as injectables and vaginal rings) become available, as these are also not coitally dependent and, to a great extent, can be used without others necessarily knowing [19].

However, although PrEP has this potential to confer agency and control in the user, it is not that simple. The only currently available PrEP method – Truvada – is the same pill that is used to treat HIV-positive persons. In many settings where HIV-associated stigma is high, being seen with “the little blue pill” (Truvada) implies being HIV infected, regardless of how a person on PrEP attempts to explain its use for prevention [18] The associated stigma may be a big disincentive for HIV-uninfected persons to take up PrEP [18, 20, 21]. Moreover, broad cultural and institutional stigma associated with sexuality, substance use and HIV may militate against access to PrEP services and engagement in related care in many settings.

### PrEP and sexuality

One of the most controversial aspects of PrEP is that of “risk compensation” [5, 15]. The fear is that PrEP users will decrease condom use or substitute PrEP for it, thereby enhancing the potential for increased sexually transmitted infections (STI), if not HIV transmission. But, arguing against PrEP based on the fact that it does not protect against other STIs is problematic in at least two ways. First, PrEP taken correctly confers as much, if not more, protection from HIV than do male latex condoms. Thus, if HIV prevention is the primary goal of PrEP, aversion of new HIV infections ought to be the outcome of relevance, and PrEP should be acknowledged as highly successful in this regard. Second, many people most at risk of acquiring HIV are the very ones who simply are not using condoms and who are, therefore, at risk of acquiring both HIV and other STIs [22]. PrEP may be an important way for these individuals to at least prevent the more dangerous disease – HIV – and, therefore, it ought not to be rejected because of what else it does not avert. Moreover, evidence to date from long-term follow-up from PrEP clinical trials and from open-label studies indicates that “risk compensation” has not occurred among either gay and other men who have sex with men (MSM) or heterosexuals using PrEP [23–25]. Although there has been incidence of hepatitis C and other STIs among those who use PrEP, no evidence yet exists to suggest that PrEP users experience increased rates of STI as compared to their at-risk counterparts.

Among gay men, fear about abandoning condoms goes beyond public health concerns and touches core issues in sexual culture. On the one hand, PrEP is creating a new form of “safe sex” that does not rely on barrier prevention methods (such as latex condoms), allowing its users to experience barrier-free intimacy without fear of contracting HIV. On the other hand, the potential for PrEP to confer a new level of agency, control and pleasure in sexual relations, in combination with the fears of “risk compensation,” has fuelled a new sexual moralism, particularly within gay communities. Early public debates in the gay community were framed around a controversial online essay that labelled PrEP users as “Truvada whores” [26–28]. Intended as a stigmatizing label, activists reappropriated the term as a message of pride and launched a PrEP campaign with T-shirts starkly emblazoned with the phrase [29]. But, the cultural association of PrEP with a kind of “unbridled” sex may have contributed to its slow uptake [7].

Scholars have noted that the so-called “PrEP wars” resemble debates over birth control for women [30]. Many of the issues raised in argument against PrEP are identical to those invoked against female contraception, namely, cost, safety, the potential impact on sexual behaviour and the potential for unforeseen health risks associated with long-term use. These issues are not new or specific to HIV, and concern about them is largely driven by a version of sexual morality – that sex is taboo, that it is self-destructive and that sexual pleasure is sinful and disgraceful [7, 31].

Despite its sociocultural baggage, PrEP already has begun to reshape the sexual landscape in many communities. For example, online sexual hook-up websites for gay men now offer an expanding variety of options for characterizing one's HIV status, with at least five HIV status options apparently now in circulation: *HIV-negative; HIV-negative and on PrEP; HIV-positive and not on treatment; HIV-positive with an undetectable viral load* and *I don't know* [32]. These provide users of the website with information they use to guide sexual practice and to imagine and define their sexual communities. The sociocultural implications of this shift are significant, as there are signs that the HIV-positive/negative binary that has persisted since the advent of the HIV test in the early 1980s may be eroding [33–35]. This trend has implications for serodiscordant relationships, as PrEP offers a way to safeguard health while preserving the relationship and promoting intimacy [36]. For heterosexual couples, PrEP provides a relatively easy way (as compared to previously favoured techniques, such as sperm washing or intrauterine insemination) to facilitate pregnancy without risking HIV transmission [37]. In short, in the context of PrEP, the risk of seroconversion is no longer the significant obstacle it has been to serodiscordant intimacy and partnership.

### The “substitutive” nature of PrEP

Beyond “risk compensation,” there are other concerns about the ways in which PrEP may become a substitute for extant HIV prevention and treatment strategies, with resultant ill effects. At a policy level, there is some concern that governments and other payers will shift resources from behavioural counselling, HIV testing, condom promotion, social support and harm reduction services to PrEP programmes, with negative consequences for certain populations [38]. Some argue that PrEP likely will not benefit those most in need – including people who use drugs and/or have mental health problems, or who experience instability in their housing situations – because of cost and adherence issues, and, as such, PrEP use might enhance existing HIV-associated disparities [39]. Still others have argued that implementing PrEP in low-resource environments would be unethical because it would threaten to shift resources away from treatment [40, 41]. These critics raise important issues of equality and justice. Their arguments point to the current institutional (financial and policy) context for funding HIV prevention in general (which in many settings is dismal) that result in the counterpoising of ART use for primary prevention and ART use for treatment.

Taking all these considerations together, it is clearly the case, as Peter Aggleton has noted, that “PrEP is an HIV prevention strategy that may be useful to some people in some contexts some of the time” [42]. If and how it is used, and with what potential effect, will vary across individuals, social groups, populations and social, political and economic systems. It is important that social scientists investigate how all this occurs and plays out globally over time, and with what consequences for individuals and societies.

## Conclusions

The promise of PrEP is not yet being fully realized, in part because not enough is being done to understand the social dynamics of the prevention strategy. From a clinical standpoint, adherence may appear to be the problem that stands between PrEP and its potential impact. But from a sociological perspective, there is a much richer set of issues that shape PrEP and its social and clinical significance. PrEP's efficacy and effectiveness – alone and in combination with other HIV prevention methods – are not simply a function of “getting drugs into bodies.”

Beyond adherence, implementing PrEP will require understanding how individuals and communities comprehend it. Do they believe it is effective? Do they trust the agencies and individuals promoting it? Do they think that they have access to it and can afford it? And, perhaps the most significant question is whether potential PrEP users understand themselves to be at risk of acquiring HIV, and, if so, whether that risk is sufficient for them to proactively engage in HIV prevention.

Beyond simple use or non-use of PrEP, social research can help us understand what meanings people assign to it. Is PrEP another “little blue pill” that they associate with “recreational” sex? Is it a symbol of love or intimacy with their partner? Is it a marker of the rich or elite who have the “privilege” to use it? PrEP's symbolic life will become just as important as its clinical efficacy in shaping how communities engage with it. Social science methods can help evaluate what impact PrEP has on sexual (and drug using) practices and cultures – beyond merely “risk compensation.” For example, “neg+PrEP” is fast becoming a new identity for gay men using online hook-up applications. How does this self-proclaimed status shape one's interactions with other men? Does taking PrEP encourage some users to explore sexual practices (such as receptive anal intercourse) that they once avoided for fear of infection? These possibilities are not just merely a matter of “risk”; they shape sexual cultures and thus have important sociological implications.

Perhaps most importantly, social science can help us reveal what PrEP tells us about the state of our public health infrastructure and the organized AIDS response community. Nearly three years after PrEP's FDA approval, the drug remains relatively underutilized. What does the slow-paced embrace of PrEP by health departments, medical providers, HIV/AIDS advocates and AIDS service organizations tell us about institutions of public health and medicine and AIDS advocacy? These socio-structural questions provide important pathways for understanding PrEP as an object circulating in social space more generally.

As interest intensifies in implementing and scaling-up PrEP in both clinical and community settings, as it now appears to be doing, the potential for social science research on PrEP – and the need to incorporate its findings – has never been greater.

## References

[1] Grant RM, Lama JR, Anderson PL, McMahan V, Liu AY, Vargas L (2010). Preexposure chemoprophylaxis for HIV prevention in men who have sex with men. N Engl J Med.

[2] Baeten JM, Donnell D, Ndase P, Mugo NR, Campbell JD, Wangisi J (2012). Antiretroviral prophylaxis for HIV prevention in heterosexual men and women. N Engl J Med.

[3] Thigpen MC, Kebaabetswe PM, Paxton LA, Smith DK, Rose CE, Segolodi TM (2012). Antiretroviral preexposure prophylaxis for heterosexual HIV transmission in Botswana. N Engl J Med.

[4] Choopanya K, Martin M, Suntharasamai P, Sangkum U, Mock PA, Leethochawalit M (2013). Antiretroviral prophylaxis for HIV infection in injecting drug users in Bangkok, Thailand (the Bangkok Tenofovir Study): a randomised, double-blind, placebo-controlled phase 3 trial. Lancet.

[5] Mansergh G, Koblin BA, Sullivan PS (2012). Challenges for HIV pre-exposure prophylaxis among men who have sex with men in the United States. PLoS Med.

[6] Rosengarten M Introduction: novel modes of inquiry to achieve effective HIV prevention.

[7] Race K (Forthcoming). Reluctant objects: sexual pleasure as a problem of HIV biomedical prevention. GLQ.

[8] Colbert C (2014). CDC updates term for ‘unprotected sex. The Bay Area Reporter [Internet].

[9] Race K (2001). The undetectable crisis: changing technologies of risk. Sexualities.

[10] Hoppe T Responsibilizing HIV-positive people through prevention: what are the implications?.

[11] AIDS Healthcare Foundation No test? No pills: truvada for HIV pre-exposure prophylaxis (PrEP) is a safety risk [Internet].

[12] Van Damme L, Corneli A, Ahmed K, Agot K, Lombaard J, Kapiga S (2012). Preexposure prophylaxis for HIV infection among African women. N Engl J Med.

[13] Molina JM, Capitant C, Charreau I, Meyer L, Spire B, Pialoux G (2015). On demand PrEP with oral TDF-FTC in MSM: results of the ANRS Ipergay Trial-23LB.

[14] Baeten JM, Heffron R (2014). Pre-exposure prophylaxis to intensify the fight against HIV. Lancet Infect Dis.

[15] McMahon JM, Myers JE, Kurth AE, Cohen SE, Mannheimer SB, Simmons J (2014). Oral pre-exposure prophylaxis (PrEP) for prevention of HIV in serodiscordant heterosexual couples in the United States: opportunities and challenges. AIDS Patient Care STDs.

[16] Potts M (1994). The urgent need for a vaginal microbicide in the prevention of HIV transmission. Am J Public Health.

[17] Hilber AM, Chersich MF, Van de Wijgert JHHM, Rees H, Temmerman M (2007). Vaginal practices, microbicides, and HIV: what do we need to know?. Sex Transm Infect.

[18] Philbin MM, Parker R, Wilson P, Grisham K, Parker C, Garcia J How Black men who have sex with men in New York city understand, talk about, and experience pre-exposure prophylaxis (PrEP).

[19] AVAC 2013 research & reality. AVAC report [Internet].

[20] Auerbach JD, Kinsky S, Brown G, Charles V (2015). Knowledge, attitudes, and likelihood of pre-exposure prophylaxis (PrEP) use among US women at risk of acquiring HIV. AIDS Patient Care STDs.

[21] van der Straten A, Stadler J, Luecke E, Laborde N, Hartmann M, Montgomery ET (2014). Perspectives on use of oral and vaginal antiretrovirals for HIV prevention: the VOICE-C qualitative study in Johannesburg, South Africa. J Int AIDS Soc.

[22] Smith DK, Herbst JH, Zhang X, Rose CE (2015). Condom effectiveness for HIV prevention by consistency of use among men who have sex with men (MSM) in the U.S. J Acquir Immune Defic Syndr.

[23] Marcus JL, Glidden DV, Mayer KH, Liu AY, Buchbinder SP, Amico KR (2013). No evidence of sexual risk compensation in the iPrEx trial of daily oral HIV preexposure prophylaxis. PLoS One.

[24] Grant RM, Anderson PL, McMahan V, Liu A, Amico KR, Mehrotra M (2014). Uptake of pre-exposure prophylaxis, sexual practices, and HIV incidence in men and transgender women who have sex with men: a cohort study. Lancet Infect Dis.

[25] Mugwanya KK, Donnell D, Celum C, Thomas KK, Ndase P, Mugo N (2013). Sexual behaviour of heterosexual men and women receiving antiretroviral pre-exposure prophylaxis for HIV prevention: a longitudinal analysis. Lancet Infect Dis.

[26] Duran D (2012). Truvada whores?. Huffington Post [Internet].

[27] Sobo J (2012). Does taking PrEP make me a whore?. Positive Frontiers [Internet].

[28] An Z, McLaughlin M, Hou J, Nam Y, Hu C, Park M (2014). Social network representation and dissemination of pre-pxposure prophylaxis (PrEP): a semantic network analysis of HIV prevention drug on Twitter. Soc Comput Soc Media.

[29] Villarreal D HIV testing counselor sells ‘Truvada whore’ shirts for AIDS charity. Towleroad [Internet].

[30] Myers JE, Sepkowitz KA (2013). A pill for HIV prevention: déjà vu all over again?. Clin Infect Dis.

[31] Venter F, Allais L, Richter M (2014). Exposure ethics: does HIV pre-exposure prophylaxis raise ethical problems for the health care provider and policy maker?. Bioethics.

[32] Barucco R Beyond “poz” and “neg”: five HIV statuses, plus a new one. Huffington Post [Internet].

[33] Sullivan A Is the HIV divide now over?. The Dish [Internet].

[34] Sobo J Choosing to love poz guys. BETA Blog [Internet].

[35] Benjamin R (2014). PrEP and prejudice. POZ Magazine [Internet].

[36] Ware NC, Wyatt MA, Haberer JE, Baeten JM, Kintu A, Psaros C (2012). What's love got to do with it? Explaining adherence to oral antiretroviral pre-exposure prophylaxis (PrEP) for HIV serodiscordant couples. J Acquir Immune Defic Syndr.

[37] Lampe MA, Smith DK, Anderson GJE, Edwards AE, Nesheim SR (2011). Achieving safe conception in HIV-discordant couples: the potential role of oral preexposure prophylaxis (PrEP) in the United States. Am J Obstet Gynecol.

[38] Baggely R Presentation at NEMUS affiliated event.

[39] Curran JW, Crosby RA (2013). Pre-exposure prophylaxis for HIV: who will benefit and what are the challenges?. Am J Prev Med.

[40] Rennie S (2013). Ethical use of antiretroviral resources for HIV prevention in resource poor settings. Dev World Bioeth.

[41] Galindo GR, Walker JJ, Hazelton P, Lane T, Steward WT, Morin SF (2012). Community member perspectives from transgender women and men who have sex with men on pre-exposure prophylaxis as an HIV prevention strategy: implications for implementation. Implement Sci.

[42] Aggleton P Social research and the response to HIV: legacy and future.

# Sub article: Pre-exposure prophylaxis in Southern Africa: feasible or not?

**DOI:** 10.7448/IAS.18.4.19979

**Published:** 2015-07-20

**Authors:** Willem Daniel François Venter, Frances Cowan, Vivian Black, Kevin Rebe, Linda-Gail Bekker

**Affiliations:** 1 Wits Reproductive Health and HIV Institute (Wits RHI), University of Witwatersrand, Johannesburg, South Africa; 2 Research Department of Infection and Population Health, University College London, London, UK; 3 Health4Men, Anova Health Institute, Cape Town, South Africa; 4 IIDMM, UCT Health Science Faculty, Cape Town, South Africa

**Keywords:** PrEP, HIV, AIDS, South Africa

## Abstract

**Introduction:**

Southern and Eastern Africa bear the brunt of the AIDS epidemic, and current prevention interventions remain inadequate. Antiretroviral-based pre-exposure prophylaxis (PrEP) is gaining momentum as an effective prevention intervention.

**Discussion:**

Discussions have been started on how this strategy could be employed in Africa such that the populations most in need can be reached urgently for the greatest impact. This requires the selection of specific risk groups and service environments in which PrEP can be distributed safely and cost effectively while being mindful of any ethical issues.

**Conclusions:**

Given the need for an integrated public health approach to this, a number of potential populations and opportunities for PrEP distribution exist and are discussed in this commentary.

## Introduction

Southern and Eastern Africa bear the brunt of the AIDS epidemic. Three out of every four people in the world commenced on antiviral therapy live in this region. South Africa alone has a remarkable 2.5 million individuals on treatment in 2014 – the largest HIV-positive population by number in the world [1].

The treatment pool in the region is ever expanding. The epidemic is firmly established within the generalized heterosexual community, but local key populations with extraordinarily high incidence, including sex workers (SWs) and men who have sex with men (MSM), as well as adolescent girls and young women make focused HIV prevention strategies that include antiretroviral pre-exposure prophylaxis (PrEP) seriously worth considering [2]. Furthermore, it may well be possible that HIV transmission attributable to particular populations in Africa, for example, fishing populations in Uganda, has been under-estimated [3, 4].

### PrEP: the evidence for effectiveness

PrEP administered to HIV-negative individuals has been shown in a number of randomized controlled trials to be effective at preventing HIV acquisition. In discordant heterosexual couples, in which adherence to the daily combination antiretroviral therapy (ART) (tenofovir (TDF)/emtricitabine (FTC) or Truvada) was particularly good, PrEP reduced HIV acquisition by 75% [5]. While the reduction in HIV acquisition among MSM in the Global iPrEx study was less striking, effectiveness was shown overall and improved relative to the number of pills taken every week and was highest (92%) among those with detectable blood levels [6]. Benefit was greatest among those men who were most likely to have unprotected receptive anal intercourse [6]. This finding has been borne out in the open label study that followed, and mounting evidence to confirm these findings is being derived from further studies and demonstration projects in MSM around the world [7–9].

Unfortunately, effectiveness evidence in young heterosexual women, to date, is mixed. Relatively good efficacy was shown among young women in two African clinical trials, but two others conducted in the region: FEM-PrEP and VOICE failed to show any efficacy of PrEP in women, due to low product use [4, 10, 11]. Research to understand the reasons for poor adherence in the context of placebo controlled trials found that the unknown efficacy, challenges with taking a daily treatment given the social risks and lack of support from partners appeared to discourage adequate product use [12]. It is likely that adherence will be greater among individuals who choose to take open label PrEP having made their own risk benefit assessment. Recent data from Cape Town confirmed that when women were given PrEP in an open label fashion in three dosing strategies including daily, intermittent with a post sex act boost and pericoital dosing, the majority of women took PrEP, although those in the daily arm were more adherent and more sexual acts were “covered” by PrEP usage [13]. While we have some modelling evidence that less adherence (as few as 4 pills per week) in MSM still can be effective in reducing infections presumably because of high concentration of drug in rectal mucosae, we do not have similar evidence in heterosexual women [14].

Both tenofovir (TDF) and emtricitabine (FTC) and the combination agent are extensively used in Africa, are available in generic formulations, and form part of the WHO's recommended first-line HIV treatment [15]. Although the US Government's Food and Drug Administration (FDA) has approved both tenofovir and Truvada (the originator combination of tenofovir and emtricitabine) for a prevention indication in both men and high-risk women in 2012, these agents are not yet licensed for prevention anywhere in Africa. The Medicines Control Council of South Africa is currently reviewing licensure in South Africa for this indication.

Relevant to the heterosexual epidemic in Africa are the novel methods for delivering PrEP that are being explored alongside the research to determine effectiveness of oral and topical agents. For example, trials testing the effectiveness of using a vaginal ring to deliver antiretrovirals are ongoing: a dapivirine-impregnated ring is being evaluated, with results due in 2016. This monthly vaginal ring could overcome the need for daily or coitally dependent adherence and potentially, as a multi-purpose technology (combining contraceptive as well as antiviral activities) may be particularly suited to the needs of adolescent women [16]. Additionally long-acting injectable depot antiretroviral agents for prevention that also have the possibility of being combined with contraceptive agents are starting safety phase trials [17].

While topical PrEP in the form of coitally administered pre- and post-vaginal dosing of a 1% tenofovir gel microbicide has been shown to be partially effective in preventing both HIV and HSV infections in young heterosexual women in a single trial in South Africa [18], a second, similar but larger, multi-site trial did not show efficacy [19]. A formulation of 1% tenofovir gel suitable for rectal mucosa is being developed for MSM and anal sex use and is currently in early clinical trials.

While clinical and efficacy data are accruing on tenofovir and tenofovir/emtricitabine PrEP usage, there have also been a number of publications over the last few years exploring the impact and cost effectiveness of this strategy in the region and in South Africa in particular. A recent systematic review of PrEP models noted that the effect of promoting PrEP to high-risk groups is highly dependent on sexual mixing patterns in the population and levels of heterogeneity in HIV risk [20]. Most of the models reported show remarkable impact in terms of infections averted, lives saved and reductions in HIV incidence, and this has been shown in both high-incidence and lower-incidence settings [21–26]. Modelling studies underscore that population-level coverage and effectiveness (which is dependent on adherence) are the main determinants of the number of infections averted and that implementation of a combination of ART and PrEP prevents more infections in a population than a programme that delivers exclusively either ART or PrEP alone. Other modelling studies suggest that a high “background” level of ART coverage is likely to increase the cost per HIV infection prevented by PrEP [25, 26].

Recent costing models have also shown that depending on product cost and coverage, the intervention of either systemic or topical PrEP is cost effective in South Africa especially if deployed to populations with particularly high incidence [21–24]. Under optimistic conditions, Walensky showed that PrEP could be cost saving in the South African context [23]. Verquet and colleagues concluded from their study that PrEP would have maximum impact and be cost effective in Southern Africa especially if male circumcision rates were low [26]. They also concluded that PrEP was best utilized as a targeted intervention added to existing strategies for epidemic control. Pretorius and colleagues examined PrEP benefit next to treatment scale–up, and they described a window of opportunity where PrEP would be most cost effective before ART has been adequately scaled up to exert prevention impact [25].

Since oral PrEP with a tenofovir-based regimen is the only PrEP currently available, the rest of this commentary focuses on this intervention.

## Discussion

### PrEP in southern/eastern Africa: feasibility

PrEP as a public health intervention is not insignificant, especially if targeted at specific higher incidence or key populations. The individuals from such populations must be found, without added stigmatization or labelling, and then be voluntarily linked to prevention programmes in which PrEP is offered. In addition, current guidelines and PrEP medications require toxicity screening that adds complexity to large-scale primary care PrEP distribution. Finally, the counselling and frequent repeated HIV testing required by current guidelines pose further challenges to full-scale implementation [27, 28]. Clinics are busy, often impersonal, and rarely offer routine screening for any intervention unless located in verticalized services. That said, antiretroviral treatment and prevention of mother to child transmission (PMTCT) have been relatively well integrated in many primary health care settings, using simplified algorithms based on the WHO public health approach. Simplification of PrEP administration, reassurance around issues of safety and relaxation of monitoring requirements will facilitate PrEP distribution by nurses supported by community care workers at primary care level.

In some countries in the region antiretroviral provision for patients well-established on treatment is moving out of health facilities and into community-based distribution centres where community care workers are instrumental in their smooth running – it is conceivable that PrEP provision would need to follow a similar path given the overcrowding of health facilities [29]. Frequent (three monthly) HIV testing to detect recent seroconversions is a key component of current PrEP programmes and is likely to become a bottleneck at health facilities as well as a disincentive to continuous and safe use of PrEP. Innovative ways of HIV self-testing and alternative models of drug delivery may be necessary. HIV self-testing is a topical issue within the region, and commercial developers have expressed interest in making these available in the region. Integrating reliable self-testing into PrEP programmes would help simplify and potentially reduce cost. South Africa has a well-funded and extensive state sector, an innovative private health sector and an extensive laboratory monitoring system, which means that access to PrEP may indeed be feasible. In other southern and eastern African countries, where the resource constraints are greater, there is still the potential to implement PrEP either through the public health system (as with PMTCT programmes) or supported by non-governmental organizations (as for condom promotion and circumcision programmes). A number of demonstration projects or open label projects are underway or are planned which will help to elucidate some of the implementation issues (Table 1).

**Table 1:** Ongoing and planned demonstration PrEP (oral and topical) and open label extension projects involving women, men, MSM, sex worker and discordant couples

Study/project	Population	Description	Location	Status
Partners demonstration project	1000 HIV serodiscordant couples	Open label Daily truvada (TVD) oral as bridge to treatment in infected partner. F/up 24 months	Kenya, Uganda	Enrolling Results 2014/15
CAPRISA 008		Open label 1% TDF vaginal gel BAT 24	South Africa	Enrolling Results 2013
CHAMPS-SA PrEP	150 young men and women (15–19 yr)	Open label TVD oral	South Africa	Enrolling Results 2015/6
SAPPHIRE FSW RCT	2800 FSW	Open label Oral daily TVD	Zimbabwe	Enrolling
TAPs: Expanded use of ART for treatment and prevention for female sex workers in South Africa	400 FSW	Open label PrEP and immediate ART for FSWLWHIV	South Africa	Enrolling
Sibanye MSM Project	200 MSM	Open label (adult MSM)	South Africa	Enrolling Results 2015/6
PrEP for MSM	300 MSM	Open label (adult MSM)	South Africa	Under review
RCTS with planned open label extensions	Population	Description	Location	Status
ASPIRE	2629 hetero women	Placebo RCT Dapivirine vaginal ring	Zimbabwe, Malawi, Uganda, South Africa	Enrolling 2015
RING study	1959 hetero women	Placebo RCT Dapivirine vaginal ring	South Africa	Enrolling 2015

### PrEP in southern/eastern Africa: implementation

There are several opportunities to provide PrEP within the region in a way that is focused and allows for sufficient levels of accountability and safety. In most cases, the feasibility is linked to the availability of established service platforms into which PrEP distribution could be integrated. A number of options are considered as follows (Table 2):

1. Adolescent girls: Girls and young women between 15 and 30 years old have extraordinary high incidence in South Africa [30–32]. The most recent household survey confirms the extreme high risk of the epidemic nationally among adolescent girls, with 15–19 year olds four times and young women in their twenties eight fold more likely to be infected than their male counterparts [32]. While there is no doubt this population could benefit from novel prevention interventions, it is less clear which service platform would be most appropriate. While there has been opposition to schools-based expanded sexual education programmes, accessing young women after they have left school is challenging, and most girls attend school until at least 16 years of age. Providing reproductive health services, including education and PrEP, at schools prior to exiting, would allow young women to be proactive in accessing services on leaving school. That said, while an accessible, captive opportunity, this platform may not be attractive for reasons of labelling and stigmatization [33, 34]. Alternatively, reviving the concept of adolescent-friendly health services and exploiting other community-based venues where adolescents share spaces and already tend to congregate may be another entry point. This may include youth drop-in centres and other testing and screening opportunities. Although not yet well developed country wide, the South African government has voiced concern about the high rate of infections in adolescent girls and may be amenable to innovative out-of-health-facility options. Johnson *et al*. compared PrEP given to five age groups in South Africa: 15–19, 20–24, 25–34, 35–49 and 50 or older. For both males and females, the age group in which it was most efficient to promote PrEP was the 15–19 age group [35], with the efficiency being greater in females than in males in this age group. Individuals who acquire HIV at younger ages have greater future potential to transmit HIV than people at older ages, and so prevention here has the greatest potential public health and cost benefit [35]. While incidence of infection among the general population of young women elsewhere in the region is lower than in South Africa, it remains very high in certain key populations, notably female SWs and MSM [36, 37]. Incidence not withstanding uptake and adherence may differ by age group due to differences in access to health services, provider attitudes about sexuality, self-agency and influence of peers and other factors that may influence adherence to pill taking.
2. Women's contraceptive services: In Southern Africa, there is extensive contraception coverage within clinics, including more recently implants, injectables, condoms and oral medication. Clinics which provide contraception could identify a high-risk population (young, sexually active women) who, if found to be HIV-negative, could be offered PrEP, along with sexual health counselling although this will depend on the setting (in some countries in the region, contraception is most easily accessible to married women after the birth of their first child).
3. Services directed at MSM: In the last decade, there has been increasing surveillance and research in the population of MSM in Africa – as in every part of the world, HIV rates have been found to be higher than the background male population rates in both gay identified and non-identified MSM [38]. There is less information in transgendered populations, but where studied, transgendered women have the highest rates of HIV [39]. Same-sex sexual relations are legal in South Africa and have been since the early 90s. Elsewhere in the region, same-sex relationships are criminalized. Throughout, public sector health services remain very heterocentric and are well known not to encourage or even enable male attendance. In addition, there is increasing recognition that men are a population being missed by HIV testing services and other prevention services [40] in general, and this is compounded in the case of MSM [41]. To this end, there has been increasing efforts to enhance male involvement in health services, scaling up sensitization of health workers to gender diversity (and in some countries) MSM sexual health training. In South Africa, there are an increasing number of clinics that are accredited to have the skill set to look after MSM. In addition, there is a strong network of Southern African GPs who have provided MSM-friendly health care to MSM for many years and may be an established and knowledgeable network to exploit.
  Brookmeyer and colleagues looked at agent-based modelling of the effectiveness of HIV prevention packages for MSM in South Africa and considered packages consisting of four components: ART; PrEP for high-risk uninfected persons; behavioural interventions to reduce rates of unprotected anal intercourse (UAI); and campaigns to increase HIV testing. They found that a four component package consisting of a 15% reduction in the rate of UAI, 50% PrEP coverage, 50% reduction in persons who never test for HIV, and 50% increase in ART coverage could prevent 33.9% of infections over 5 years (95% confidence interval, 31.5–36.3). The package components with the largest incremental prevention effects were UAI reduction and PrEP coverage. The impact of increased HIV testing was magnified in the presence of PrEP [42].
4. Sex workers: South Africa has an expanding verticalized SW programme, partly government and partly donor funded, often combined with other programmes, such as trucker heath programmes. Likewise in Zimbabwe, there is a national network of clinical services specifically for SWs supported by a peer educator delivered community empowerment programme. Kenya has articulated the need for a specific SW prevention programme. The clinical care for SWs is essentially a reproductive health package with intensive HIV testing, and integrating PrEP would be relatively simple. While risk compensation and subsequent “condom migration” is a potential concern, in reality, many women struggle to use condoms consistently [43]. The potential implications for other sexually transmitted infections (STIs) and unintended pregnancy need to be added to the broader health implications of promoting antiretroviral-based prevention methods if this risk is likely [44].
  In South Africa, it has been estimated that between 6 and 11% of adult HIV transmission has been attributable to commercial sex, but in other regions in which the HIV epidemic is more concentrated, SW-specific interventions may be relatively more important [44]. For example, other models suggest that providing a topical gel to SWs would reduce HIV incidence in the general population by only 9% in the South African context, compared to 48% in Benin [45], where commercial sex is estimated to account for more than half of HIV infections in men [46]. Previous modelling has also shown that SW interventions are likely to have relatively less impact in mature epidemics than in early-stage epidemics [47]. Bekker *et al*. recently published a model simulation based on the South African heterosexual epidemic which indicated that condom promotion and distribution programmes in South Africa had already reduced HIV incidence in SWs and their clients by more than 70%. Under optimistic model assumptions, PrEP together with ‘test and treat’ programmes could further reduce HIV incidence in South African SWs and their clients by 40% or more in future. The authors suggested that the addition of these biomedical approaches to a prevention package that included behavioural and structural components in consultation with SW communities would go far in reducing HIV infection in sex work in many different settings worldwide [44].
5. Pregnant women: Most women in South Africa deliver within a health facility; HIV testing, PMTCT and initiation of ART during pregnancy has reached relatively high levels. Some studies have demonstrated high levels of seroconversion, and may account for some of the small percentage of PMTCT failures. Initiating PrEP during pregnancy has some theoretical toxicity concerns for the foetus, but the antenatal verticalized service would be ideal to initiate and maintain HIV-negative pregnant women [48].
6. Serodiscordant couples: Serodiscordant couples make up an appreciable proportion of all partnerships in the region, and UNAIDS estimates this is where the greatest number of transmissions occur [49, 50]. While national and international guidelines have identified serodiscordant couples as a priority for treatment as prevention (where the index partner takes ART regardless of CD4 count), using PrEP during the period before virological suppression of the index partner is achieved or in situations where the index partner declines to start or is poorly adherent to treatment may be indicated. The option of PrEP for serodiscordant couples could be discussed at HIV testing services or within treatment services. Additionally, there are limited options for safe conception for serodiscordant couples in the region, a problem when the desire and cultural expectation for couples to have children is strong [51, 52]. Peripartum PrEP to cover the period of conception and pregnancy has the potential to reduce HIV acquisition in this specific circumstance [53, 54].
  Extending access beyond this, especially to heterosexual men, may require a focus on workplaces and other venue-based contact. Drug users, including injecting drug users, have also been shown to derive benefit from PrEP [55]. The region has a growing population of injecting drug users, and it will be important to consider PrEP in any combination package for this risk group. Targeting also assumes nuanced understanding of where highest incidence is present. This idea of tracking “hot spots,” while apparently an efficient deployment of this prevention tool requires sophisticated, flexible and dynamic surveillance with huge flexibility in health systems to quickly and effectively respond. On the other hand, targeting high-burden areas and/or populations may offer a rational, phased approach to general prevention scale-up [56].

**Table 2:** Potential population types suitable for implementation of pre-exposure prophylaxis prevention strategies in Southern Africa

Population type	Potential venue	Demo. projects underway in the region	Potential barriers
Adolescent girls	School health services, community-based adolescent venues, adolescent-friendly health services	√	Consent, participation, adherence, regular testing
Contraceptive services	Contraception services	×	Participation, adherence, regular testing
MSM	Mens’ health services, MSM specific services	√	Regular testing, available services, homoprejudice and criminalization
Sex workers (SWs)	SW services, SW venues	√	Criminalization, regular testing, labelling and stigma
Pregnant women	Antenatal care services	×	Participation, regular testing, continued use
Discordant couples	Safer conception services, HIV care services	√	Participation, adherence, regular testing
Men	Work venues	×	Acceptance by work health programmes, regular testing

### PrEP in southern/eastern Africa: ethical issues

The ethics of PrEP have been extensively discussed in the HIV and ethics literature [57, 58]. In summary, concerns have largely centred on displacement of antiretroviral resources for treatment, prevention in HIV-negative people, issues of resistance, and sexual risk disinhibition. We believe that PrEP raises no new PrEP-specific ethical issues and that dilemmas may be tackled using conventional ethical frameworks, for all the ethical concerns raised within the literature.

Authors have argued on the displacement issue that this is a conventional debate around resource allocation and that balancing prevention and treatment is relatively straightforward; treatment takes precedence when it comes to allocation of antiretroviral products, although even this stance has been challenged [59]. In South Africa, there is currently adequate budget for treatment, and extensive resources allocated to prevention, although there are substantial funding gaps elsewhere in the region [60]. The tenofovir/emtricitabine combination is available at a fraction of the cost of what it is sold for in developed countries. Commercial cost of generic tenofovir is currently around $10 per month, and generic tenofovir/emtricitabine is approximately $30 per month. Government tenders drive these prices even lower. So, rationing due to cost may be less of an issue than it would be in poorer nations, and paradoxically due to low cost could be more available than in richer countries.

Resistance has not been a problem in the clinical trials to date; empiric research will certainly be tracking this, but this is more of a possible future public health threat than a current ethical dilemma. New classes of drugs, as well as drugs with higher resistance barriers within existing classes, have been licensed at extraordinary low cost within the country, meaning that alternatives are likely, even if worst case scenarios around drug resistance occur. However, modelling at least suggests that PrEP is unlikely to pose a problem for antiretroviral resistance [36]. Interestingly, a number of published model analyses have suggested that HIV drug resistance in a population would be largely driven by ART, not PrEP, likely as a result of insufficient ART adherence or lack of viral load monitoring in ART programmes, leading to selection of resistant variants during incomplete viral suppression [36].

The issue of disinhibition or greater risk taking in the face of perceived added protection, has been much debated for all types of HIV prevention, including medical male circumcision, condoms and microbicides, and follows even older debates around contraception and the ‘morning-after’ pill. While causing much theoretical concern, very little tangible evidence exists for this phenomenon. Yet this remains a source of uncertainty when considering the potential effect of oral and topical PrEP. Some modelling studies have also raised this concern [20, 61, 62].

### PrEP in Southern Africa: financing

South Africa is a middle-income country, with a well-resourced private sector and an extensive state funded programme for HIV/AIDS treatment and prevention. The state HIV treatment programme is almost totally financed from the national fiscus, with some key donor support [1]. Prevention programmes including PrEP provision within these programmes are possible but must be sourced. The South African national treasury has used similar modelling to plan for the budgeting of the broad antiretroviral programme and would probably look favourably to proposals addressing this provision. Other countries in the region are largely considered low-income countries, with a much higher proportion of HIV prevention and care funded through donors [60]. However, the case for investing in PrEP, particularly for those at highest risk, remains sound. Kenya in particular has written PrEP into its plan for HIV eradication by 2030 [63].

The price of currently available PrEP is a fraction of that in developed countries, with generic competition. There is accumulating evidence that tenofovir alone is adequate for protection and, if confirmed, may mean the price of PrEP falls further.

## Conclusions

In sub-Saharan Africa there were an estimated 1.6 million new HIV infections in 2012 alone [64]. With a regional health system that is buckling under the load of HIV and TB diagnosis, linkage, care and retention, finding effective and easily implementable ways to reduce new HIV infections is an important undertaking. Targeted PrEP and tailored combination prevention including PrEP may well provide a useful additional intervention in Africa's ongoing movement towards epidemic control.

## References

[1] Bekker LG, Venter F, Cohen K, Goemare E, Van Cutsem G, Boulle A (2014). Provision of antiretroviral therapy in South Africa: the nuts and bolts. Antivir Ther.

[2] What works for women and girls.

[3] Pruden H, Watts C, Vickerman P, Bobrova N, Heise L, Ogungbemi M (2013). Can the UNAIDS modes of transmission model be improved? A comparison of the original and revised model projections using data from a setting in west Africa. AIDS.

[4] Kiwanuka N, Ssetaala A, Nalutaaya A, Mpendo J, Wambuzi M, Nanvubya A (2014). High incidence of HIV-1 infection in a general population of fishing communities around Lake Victoria, Uganda. PLoS One.

[5] Baeten JM, Donnell D, Ndase P, Mugo NR, Campbell JD, Wangisi J (2012). Antiretroviral prophylaxis for HIV prevention in heterosexual men and women. N Engl J Med.

[6] Grant RM, Lama JR, Anderson PL, McMahan V, Liu AY, Vargas L (2010). Pre exposure chemoprophylaxis for HIV prevention in men who have sex with men. N Engl J Med.

[7] Grant RM, Anderson PL, McMahan V, Liu A, Amico KR, Mehrotra M (2014). Uptake of pre-exposure prophylaxis, sexual practices, and HIV incidence in men and transgender women who have sex with men: a cohort study. Lancet Infect Dis.

[8] AVAC PrEP in Europe leaps ahead with PROUD result.

[9] Buchbinder S, Glidden D, Liu A, McMahan V, Guanira J, Mayer K (2014). HIV pre-exposure prophylaxis in men who have sex with men and transgender women: a secondary analysis of a phase 3 randomised controlled efficacy trial. Lancet Infect Dis.

[10] Baeten J, Haberer J, Liu A, Sista N (2013). Preexposure prophylaxis for HIV prevention: where have we been and where are we going?. J Acquir Immune Defic Syndr.

[11] Murnane P, Celum C, Mugo N, Campbell J, Donnell D, Bukusi E (2013). Efficacy of preexposure prophylaxis for HIV-1 prevention among high-risk heterosexuals: subgroup analyses from a randomized trial. AIDS.

[12] van der Straten A, Stadler J, Montgomery E, Hartmann M, Magazi B, Mathebula F (2014). Women's experiences with oral and vaginal pre-exposure prophylaxis: the VOICE-C qualitative study in Johannesburg, South Africa. PLoS One.

[13] Bekker L-G, Hughes J, Amico R, Roux S, Hendrix C, Anderson PL (2015). HPTN 067/ADAPT Cape Town: a comparison of daily and nondaily PrEP dosing in African women.

[14] Anderson PL, Glidden DV, Liu A, Buchbinder S, Lama JR, Guanira JV (2012). Emtricitabine–tenofovir concentrations and pre-exposure prophylaxis efficacy in men who have sex with men. Sci Transl Med.

[15] South African treatment guidelines (2013). http://www.sahivsoc.org/upload/documents/2013%20ART%20Guidelines-Short%20Combined%20FINAL%20draft%20guidelines%2014%20March%202013.pdf.

[16] Friend DR, Clark JT, Kiser PF, Clark MR (2013). Multipurpose prevention technologies: products in development. Antiviral Res.

[17] Spreen WR, Margolis DA, Pottage JC (2013). Long-acting injectable antiretrovirals for HIV treatment and prevention. Curr Opin HIV AIDS.

[18] Abdool Karim Q, Abdool Karim SS, Frohlich JA, Grobler AC, Baxter C, Mansoor LE (2010). Effectiveness and safety of tenofovir gel, an antiretroviral microbicide, for the prevention of HIV infection in women. Science.

[19] Rees H, Delany-Moretlwe S, Baron D, Lombard C, Gray G, Myer L FACTS 001 Phase III trial of pericoital tenofovir 1% gel for HIV prevention in women.

[20] Gomez GB, Borquez A, Case KK, Wheelock A, Vassall A, Hankins C (2013). The cost and impact of scaling up pre-exposure prophylaxis for HIV prevention: a systematic review of cost-effectiveness modelling studies. PLoS Med.

[21] Terris-Prestholt F, Foss AM, Cox AP, Heise L, Meyer-Rath G, Delany-Moretlwe S (2014). Cost-effectiveness of tenofovir gel in urban South Africa: model projections of HIV impact and threshold product prices. BMC Infect Dis.

[22] Williams B, Abdool Karim S, Abdool Karim Q, Gouws E (2011). Epidemiological impact of tenofovir gel on the HIV epidemic in South Africa. J Acquir Immune Defic Syndr.

[23] Walensky RP, Park JE, Wood R, Freedberg KA, Scott CA, Bekker LG (2012). The cost-effectiveness of pre-exposure prophylaxis for HIV infection in South African women. Clin Infect Dis.

[24] Vissers DC, Voeten HA, Nagelkerke NJ, Habbema JD, de Vlas SJ (2008). The impact of pre-exposure prophylaxis (PrEP) on HIV epidemics in Africa and India: a simulation study. PLoS One.

[25] Pretorius C, Stover J, Bollinger L, Bacaër N, Williams B (2010). Evaluating the cost-effectiveness of pre-exposure prophylaxis (PrEP) and its impact on HIV-1 transmission in South Africa. PLoS One.

[26] Verquet S (2013). Where to deploy pre-exposure prophylaxis in Sub-Saharan Africa?. Sex Transm Infect.

[27] Guidelines: Southern African guidelines for the safe use of pre-exposure prophylaxis in men who have sex with men who are at risk for HIV infection.

[28] Preexposure prophylaxis for the prevention of HIV infection in the United States – 2014 clinical practice guideline. http://www.cdc.gov/hiv/pdf/prepguidelines2014.pdf.

[29] Grimsrud A, Sharp J, Kalombo C, Bekker L-G, Myer L (2015). Implementation of community-based adherence clubs for stable antiretroviral therapy patients in Cape Town, South Africa. J Int AIDS Soc.

[30] Middelkoop K, Myer L, Mark D, Mthimunye SP, Smit J, Wood R (2008). Adolescent and adult participation in an HIV vaccine trial preparedness cohort in South Africa. J Adolesc Health.

[31] Pettifor A, Bekker LG, Hosek S, DiClemente R, Rosenberg M, Bull SS (2013). Preventing HIV among young people: research priorities for the future. J Acquir Immune Defic Syndr.

[32] Shisana O, Rehle T, Simbayi LC, Zuma K, Jooste S, Zungu N (2014). South African national HIV prevalence, incidence and behaviour survey, 2012.

[33] Jackson CA, Henderson M, Frank JW, Haw SJ (2012). An overview of prevention of multiple risk behaviour in adolescence and young adulthood. J Public Health.

[34] Harrison A, Newell ML, Imrie J, Hoddinott G (2010). HIV prevention for South African youth: which interventions work? A systematic review of current evidence. BMC Public Health.

[35] Bruce F, Johnson L, Welte A Understanding the impact of an HIV intervention package for adolescents.

[36] van de Vijvera D, Nichols B, Abbas U, Boucher C, Cambiano V, Eaton JE (2013). Preexposure prophylaxis will have a limited impact on HIV-1 drug resistance in sub-Saharan Africa: a comparison of mathematical models. AIDS.

[37] Interagency-Working-Group-on-Key-Populations (2014). HIV and young people who sell sex: a technical brief (draft).

[38] Beyrer C, Baral SD, van Griensven F, Goodreau SM, Chariyalertsak S, Wirtz AL (2012). Global epidemiology of HIV infection in men who have sex with men. Lancet.

[39] Baral SD, Poteat T, Strömdahl S, Wirtz AL, Guadamuz TE, Beyrer C (2013). Worldwide burden of HIV in transgender women: a systematic review and meta-analysis. Lancet Infect Dis.

[40] Skovdal M, Campbell C, Madanhire C, Mupambireyi Z (2011). Masculinity as a barrier to men's use of HIV services in Zimbabwe. Global Health.

[41] Baral S, Scheibe A, Sullivan P, Trapence G, Lambert A, Bekker LG (2013). Assessing priorities for combination HIV prevention research for men who have sex with men (MSM) in Africa. AIDS Behav.

[42] Brookmeyer R, Boren D, Baral S, Bekker LG, Phaswana-Mafuya N, Beyrer C (2014). Combination HIV prevention among MSM in South Africa: results from agent based modeling. PLoS One.

[43] Cowan FM, Mtetwa S, Davey C, Fearon E, Dirawo J, Wong-Gruenwald R (2013). Engagement with HIV prevention treatment and care among female sex workers in Zimbabwe: a respondent driven sampling survey. PLoS One.

[44] Bekker LG, Johnson L, Cowan F, Overs C, Besada D, Hillier S (2015). Combination HIV prevention for female sex workers: what is the evidence?. Lancet.

[45] Vickerman P, Watts C, Delany S, Alary M, Rees H, Heise L (2006). The importance of context: model projections on how microbicide impact could be affected by the underlying epidemiologic and behavioral situation in 2 African settings. Sex Transm Dis.

[46] Lowndes CM, Alary M, Belleau M, Kofi Bosu W, Federic Kintin D, Asonye Nnorom J (2008). West Africa HIV/AIDS epidemiology and response synthesis.

[47] Boily M, Lowndes C, Alary M (2002). The impact of HIV epidemic phases on the effectiveness of core group interventions: insights from mathematical models. Sex Transm Infect.

[48] Whetham J, Taylor S, Charlwood L, Keith T, Howell R, McInnes C (2014). Pre-exposure prophylaxis for conception (PrEP-C) as a risk reduction strategy in HIV-positive men and HIV-negative women in the UK. AIDS Care.

[49] UNAIDS (2010). 2010 Report on the global AIDS epidemic.

[50] Dunkle KL, Stephenson R, Karita E, Chomba E, Kayitenkore K, Vwalika C (2008). New heterosexually transmitted HIV infections in married or cohabiting couples in urban Zambia and Rwanda: an analysis of survey and clinical data. Lancet.

[51] Cooper D, Moodley J, Zweigenthal V, Gail-Bekker L, Shah I, Myer L (2009). Fertility intentions and reproductive health care needs of people living with HIV in Cape Town, South Africa: implications for integrating reproductive health and HIV care services. AIDS Behav.

[52] Laher F, Todd C, Stibich M, Phofa R, Behane X, Mohapi L (2009). A qualitative assessment of decisions affecting contraceptive utilization anbd fertility intentions among HIV positive women in Soweto, South Africa. AIDS Behav.

[53] Matthews L, Baeten J, Celum C, Bangsberg D (2010). Periconception pre-exposure prophylaxis to prevent HIV transmission: benefits, risks, and challenges to implementation. AIDS.

[54] Matthews L, Mukherjee J (2009). Strategies for harm reduction among HIV-affected couples who want to conceive. AIDS Behav.

[55] Choopanya K, Martin M, Suntharasamai P, Sangkum U, Mock PA, Leethochawalit M (2013). Antiretroviral prophylaxis for HIV infection in injecting drug users in Bangkok, Thailand (the Bangkok Tenofovir Study): a randomised, double-blind, placebo-controlled phase 3 trial. Lancet.

[56] Jones A, Cremin I, Abdullah F, Idoko J, Cherutich P, Kilonzo N (2014). Transformation of HIV from pandemic to low-endemic levels: a public health approach to combination prevention. Lancet.

[57] Venter WDF, Allais L, Richter M (2014). Exposure ethics: does HIV pre-exposure prophylaxis raise ethical problems for the health care provider and policy maker?. Bioethics.

[58] Abdool Kariem Q, Bayer R (2013). Special issue: anti-retrovirals for treatment and prevention – new ethical challenges. Develop World Bioeth.

[59] Rennie S (2013). Ethical use of antiretroviral resources for HIV prevention in resource poor settings. Dev World Bioeth.

[60] UNAIDS (2013). Access to antiretroviral therapy in Africa status report on progress towards the 2015 targets.

[61] Foss AM, Vickerman PT, Heise L, Watts CH (2003). Shifts in condom use following microbicide introduction: should we be concerned?. AIDS.

[62] Karmon E, Potts M, Getz WM (2003). Microbicides and HIV: help or hindrance?. J Acquir Immune Defic Syndr.

[63] Kenya's Prevention Revolution Road Map Count down to 2030.

[64] HIV/AIDS TJUNPo (2013). UNAIDS global fact sheet 2013.

# Sub article: HIV pre-exposure prophylaxis and health and community systems in the Global South: Thailand case study

**DOI:** 10.7448/IAS.18.4.19953

**Published:** 2015-07-20

**Authors:** Donn Colby, Kriengkrai Srithanaviboonchai, Suphak Vanichseni, Sumet Ongwandee, Nittaya Phanuphak, Michael Martin, Kachit Choopanya, Suwat Chariyalertsak, Frits van Griensven

**Affiliations:** 1 SEARCH, Thai Red Cross AIDS Research Centre, Bangkok, Thailand; 2 Thai Red Cross AIDS Research Centre, Bangkok, Thailand; 3 Research Institute for Health Sciences, Chiang Mai University, Chiang Mai, Thailand; 4 Department of Community Medicine, Faculty of Medicine, Chiang Mai University, Chiang Mai, Thailand; 5 Bangkok Tenofovir Study Group, Bangkok, Thailand; 6 Bureau of AIDS, TB and STI, Department of Disease Control, Ministry of Public Health, Nonthaburi, Thailand; 7 Thailand Ministry of Public Health – U.S. Centers for Disease Control and Prevention Collaboration, Nonthaburi, Thailand; 8 U.S. Centers for Disease Control and Prevention, Division of HIV/AIDS Prevention, Atlanta, GA, USA; 9 Division of Preventive Medicine and Public Health, University of California – San Francisco San Francisco, CA, USA

**Keywords:** HIV, PrEP, Thailand, prevention, prophylaxis

## Abstract

**Introduction:**

Pre-exposure prophylaxis (PrEP) is recommended by the World Health Organization as an effective method of HIV prevention for individuals at risk for infection. In this paper, we describe the unique role that Thailand has played in the global effort to combat the HIV epidemic, including its role in proving the efficacy of PrEP, and discuss the opportunities and challenges of implementing PrEP in a middle-income country.

**Discussion:**

Thailand was one of the first countries in the world to successfully reverse a generalized HIV epidemic. Despite this early success, HIV prevalence has remained high among people who inject drugs and has surged among men who have sex with men (MSM) and transgender women (TGW). Two pivotal trials that showed that the use of oral antiretroviral medication as PrEP can reduce HIV transmission were conducted partially or entirely at Thai sites. Demonstration projects of PrEP, as well as clinical trials of alternative PrEP regimens, began or will begin in 2014–2015 in Thailand and will provide additional data and experience on how to best implement PrEP for high-risk individuals in the community. Financing of drug costs, the need for routine laboratory monitoring and lack of awareness about PrEP among at-risk groups all present challenges to the wider implementation of PrEP for HIV prevention in Thailand.

**Conclusions:**

Although significant challenges to wider use remain, PrEP holds promise as a safe and highly effective method to be used as part of a combined HIV prevention strategy for MSM and TGW in Thailand.

## Introduction

Thailand, located in Southeast Asia with a population of 65.5 million [1], has a unique position in the global response to the HIV epidemic. It has the highest adult HIV prevalence (1.2% in 2012) in Asia [2], while at the same time being hailed as the first nation to successfully control and reverse a generalized HIV epidemic [3].

In the early 1990s, Thailand faced a generalized HIV epidemic, with national HIV prevalence peaking at 4.0% among male army recruits in 1993 [4] and 2.6% among pregnant women attending antenatal clinics in 1995 [5]. Much credit for reversing the epidemic has been given to the National HIV/AIDS Control Program, formed in 1991, with strong national leadership, and centred on the “100% condom use during commercial sex” program [6–10]. Since 2005, national sentinel surveillance has shown HIV prevalence of 0.5% among male military recruits and 0.5–0.6% among pregnant women [11].

While Thailand has had great success in addressing HIV transmission among the heterosexual population, emerging epidemics of HIV infection were recognized among men who have sex with men (MSM) and among transgender women (TGW) in the past decade. Although the number of new HIV infections among people who inject drugs (PWID) and heterosexuals has declined dramatically, the number of new infections among MSM has slowly increased (Figure 1). In 2003, a cross-sectional venue-based survey in Bangkok found an HIV prevalence of 17.3% among MSM [13] and in 2005 an HIV prevalence of 13.5% was found among TGW enrolled from entertainment and other venues in Bangkok, Chiang Mai and Phuket [14, 15]. By 2007, the HIV prevalence among MSM in Bangkok had almost doubled to 30.8% [16]. Average HIV prevalence among TGW in the three cities of Bangkok, Chiang Mai and Phuket in 2010 was 10.1% [17]. HIV incidence rates, especially among young MSM aged 18–21 years, are alarmingly high: 8.4 per 100 person-years (PY) in Chiang Mai (2008–2009) [18], 9.7 per 100 PY in Pattaya (2009–2010) [19] and 12.2 per 100 PY in Bangkok (2005–2011) [20].

**Figure 1:** Annual new HIV infections in Thailand by risk category, 1988–2015. Adapted from Ref. [12].

From a generalized epidemic driven largely by heterosexual sex and the male clients of female sex workers in the 1990s, by 2015 the HIV epidemic in Thailand had evolved into a concentrated epidemic in which the MSM population is the most affected [11].

Pre-exposure prophylaxis (PrEP) with antiretroviral drugs has been proven to be effective in reducing the transmission of HIV infection among MSM and other key populations at risk. In this paper, we describe the unique role that Thailand has played in the global effort to combat the HIV epidemic, including its role in proving the efficacy of PrEP, and discuss the opportunities and challenges of implementing PrEP in the country.

## Discussion

### HIV prevention research in Thailand

Having participated in seven HIV preventive Phase III efficacy trials, Thailand ranks second only to South Africa worldwide in the number of biomedical HIV prevention trials conducted within the country [3]. It is also the only country in the world to host Phase III trials showing efficacy of a prime-boost HIV vaccine in the general population [21] and of oral PrEP in two distinct high-risk populations, MSM and PWID [22, 23].

Completed and ongoing PrEP clinical trials and demonstration projects in Thailand are listed in Table 1. The iPrEx study was the first clinical trial to show efficacy (44% reduction in HIV infection over all sites) of oral PrEP using a combination of tenofovir disoproxil fumarate (TDF) and emtricitabine (FTC) [22]. The study enrolled 2499 MSM and TG participants in six countries, of whom 114 enrolled in the Prevention of Infection in Man (PIMAN) Clinic in Chiang Mai, Thailand. The efficacy of PrEP was highly dependent on adherence; fewer than half of the subjects in the active treatment arm of the study had detectable serum levels of study drugs, but those that did had a 92% reduction in HIV infection risk.

**Table 1:** PrEP clinical trials and demonstration projects implemented and planned in Thailand

Project title	Time period	Project design	Locations	Thai agency	Intervention	N Thailand (total)	Ref.
iPrEX	2007–2009	Phase III Clinical trial	Chiang Mai	PIMAN, Chiang Mai University	TDF-FTC daily	114 (2499) MSM, TGW	[22]
BTS	2005–2012	Phase III Clinical trial	Bangkok	TUC, BMA	TDF daily	2413 (2413) PWID	[23]
HPTN 067	2011–2014	Phase II Clinical trial	Bangkok	TUC	TDF-FTC daily vs. intermittent	193 (622) MSM, TGW	[25]
MTN 017	2013–	Phase II Crossover trial	Bangkok, Chiang Mai	TUC, Chiang Mai University	TDF-FTC daily vs. daily/ intermittent rectal gel	54 (186) MSM, TGW	[26]
PrEP-30	2014–	Demonstration	Bangkok	Thai Red Cross AIDS Research Center	Self-paid TDF-FTC daily	Unlimited	N/A
Test, treat and prevent	2015–	Implementation science	Bangkok+4 cities	Thai Red Cross AIDS Research Center	TDF-FTC daily	2000 (2000) MSM, TGW	[36]

PIMAN, Prevention of Infection in Man; TUC, Thailand Ministry of Public Health – US Centers for Disease Control and Prevention Collaboration; BMA, Bangkok Metropolitan Administration.

The Bangkok Tenofovir Study (BTS) evaluated oral PrEP with tenofovir alone in 2413 PWID at 17 drug treatment clinics in Bangkok and showed a 49% reduction in HIV incidence [23]. Again, adherence to the study medicine was important; efficacy increased to 56% among participants who reported taking study medication ≥71% of the time, and to 74% among those with detectable plasma tenofovir.

Thai sites continue to participate in ongoing PrEP clinical trials. The HIV Prevention Trials Network (HPTN) 067 study is a Phase II, three-armed randomized trial of the pharmacokinetics and behavioural aspects of intermittent versus daily oral TDF-FTC for the prevention of HIV infection among MSM, TGW and high-risk heterosexual women [24]. The study enrolled 193 MSM and TGW in Thailand, 238 in the United States, and 191 high-risk heterosexual women in South Africa. However, the data reported thus far do not disaggregate MSM and TGW and it remains to be seen if the trial included a significant number of TGW participants. Two Thai sites, in Bangkok and Chiang Mai, are included in the Microbicide Trials Network (MTN) 017 study, a Phase II randomized crossover trial of the safety and acceptability of daily or sexual event–driven 1% tenofovir reduced glycerin rectal gel versus daily oral TDF-FTC [25].

A notable gap in the evidence base for oral PrEP is the very low number of TGW who have participated in PrEP trials to date [26]. Transgender individuals in Thailand and elsewhere, both male-to-female and female-to-male, experience exposure to genital reassignment surgery and hormone use with as yet unknown effects on HIV risk or on the efficacy or oral PrEP [26, 27]. More research is needed on the role of PrEP for HIV prevention in this unique population before it can be concluded that PrEP will be as efficacious as it is in other high-risk populations.

### Acceptability of PrEP among high-risk populations in Thailand

Knowledge about PrEP among Thai MSM has been reported to vary greatly, from 7% in an online survey [28] to 66% of MSM recruited in entertainment venues in a large urban area [29]. The latter survey was conducted in Chiang Mai City, which hosted one of the sites that implemented the iPrEx study and may therefore account for the higher awareness of PrEP in that location.

Three surveys have found that willingness to use PrEP was 36–41% among Thai MSM and TGW [28–30]. One survey reported that 85% of MSM said that they would “probably” or “definitely” use PrEP if it were available [30]. One-quarter (24%) of online respondents said that they would not take PrEP even if it were offered for free, but 65% stated that they would be willing to pay for PrEP medication [28]. Most (65%) of those were willing to pay a maximum of only 750 Thai Baht (THB), or about US$25, per month for PrEP. When given a choice between routes of PrEP administration, the majority chose a daily oral pill over event-driven oral administration or intermittent intramuscular injections [30].

Rectal microbicides have not yet been proven to decrease the risk of HIV transmission, but are in development, and Phase II clinical trials are being conducted in Thailand and other countries [25]. Among a cohort of MSM in Bangkok, 79% said that they would be willing to participate in an efficacy trial of a rectal microbicide and 97% would be willing to use a rectal gel if it were found to be effective [31].

There have been no published reports of willingness to use PrEP among PWID in Thailand. In addition, neither the World Health Organization nor the Thai advocacy groups such as the Thai AIDS Treatment Action Group have thus far publicly endorsed PrEP for PWID [32, 33].

### PrEP implementation projects in Thailand

Both the iPrEx trial and the BTS included open-label extensions of PrEP for study participants after the efficacy trials were completed, which provide initial experience in implementing PrEP in Thailand. In each trial, HIV-negative participants were informed of the study results and offered drug and follow-up free of charge for one to two years.

The iPrEx trial enrolled 114 participants in Chiang Mai, Thailand. Of these, 61 (54%) HIV-negative MSM continued in the extension phase after completion of the main study and 54 (89%) of these chose to take oral TDF-FTC as PrEP for up to an additional 18 months [34]. Follow-up visits took place every 4–12 weeks at a community-based clinic. Adherence in the extension phase was high: 43/54 (80%) of Thai MSM tested had detectable plasma tenofovir, higher than the combined 71% of subjects worldwide. Study staff noted that participants perceived a clear benefit to PrEP use and were more open to discussing and problem-solving difficulties with adherence than they were during the clinical trial phase of the study, when they did not know if they were receiving drug or placebo and adherence was monitored more stringently (S. Chariyalertsak, personal communication, 2014).

The BTS extension provided open-label PrEP for one year via directly observed treatment (DOT) to study participants in the 17 drug treatment clinics and in prisons. A total of 1327 BTS participants returned to receive study results and 785 (59%) chose to take TDF [35]. Among the 128 incarcerated participants, 94% missed fewer than eight doses of TDF during the previous 28 days at their most recent assessment. Among non-incarcerated subjects, however, only 15% met this adherence threshold. Daily PrEP was dispensed as DOT in drug treatment clinics and the need for daily travel may be responsible, in part, for the low adherence among BTS participants in the community. The study investigators concluded that additional adherence support and distribution mechanisms may be needed to make PrEP more acceptable and accessible to non-incarcerated PWID.

Two additional PrEP demonstration projects are scheduled to begin providing PrEP to MSM and TGW in Bangkok and several other cities in early 2015. The Test, Treat, and Prevent HIV program is supported by the Thailand Ministry of Public Health and PEPFAR and plans to enrol 8000 high-risk (defined as having anal intercourse without using a condom at least once in the previous six months) MSM and TGW, including sex workers [36, 37]. Participants testing HIV positive will be offered antiretroviral therapy (ART) regardless of CD4 count and 600 of those testing negative will be offered PrEP with one-year follow-up. The trial will be conducted at both facility- and community-based sites.

The Thai Red Cross AIDS Research Centre in Bangkok, which operates the largest HIV testing and counselling centre in Thailand, also plans to implement a demonstration project of fee-based PrEP for all high-risk individuals, including MSM, TGW, heterosexuals and PWID. The PrEP-30 project aims to evaluate the feasibility of self-pay PrEP as a sustainable model not reliant on government or external funding. A complete HIV prevention package, including locally produced TDF-FTC, counselling and laboratory testing will be provided for a fee of 30 THB per day, or about US$30 per month. Although Thailand is an upper middle-income country with a per capita income of US$445 per month [38], the willingness of at-risk individuals to pay out of pocket for PrEP remains untested.

The Thai government along with national and international non-governmental organizations held a meeting with Thai experts and stakeholders in August 2012 to discuss the use of ARV drugs for HIV prevention [39]. One conclusion of the meeting was that the goal of an AIDS-free Thailand could not be achieved through the expansion of the existing behavioural interventions alone, and that the use of ARV drugs through expanded treatment and PrEP would eventually become part of the national AIDS strategy. Concerns raised in regard to PrEP included stigma and discrimination reducing access to key populations, the cost of drugs, lack of spare capacity in the healthcare system, the risk of side effects when giving drugs to a healthy population and the potential for HIV resistance to develop. Among the recommendations that were made to the Ministry of Public Health were to implement operational research on the use of PrEP for key affected populations and to consider task-shifting for service delivery.

### Opportunities and challenges for PrEP implementation in Thailand

The HIV epidemic among MSM in Thailand continues unabated with high incidence and prevalence. Similar to experiences elsewhere, past and current HIV prevention programming has had limited efficiency or success in decreasing the transmission of HIV in this population [40]. The Test and Treat strategy has the potential to decrease HIV transmission in the community by the early recognition and treatment of HIV infection, but the numbers (or proportion) of MSM covered by Test and Treat programs at present will not be enough to affect the overall epidemic. New and expanded HIV prevention modalities are needed if the HIV epidemic among MSM is to be controlled. The Thai National Guidelines on HIV/AIDS Treatment and Prevention were revised in 2014 and for the first time recommended PrEP for key populations [41].

Significant challenges to the wider use of PrEP remain, the foremost being finding the resources, both human and financial, that would be required to make PrEP available to all potential users in Thailand. Even with the availability of low-cost generic TDF-FTC, drug costs will be substantial if tens of thousands of otherwise healthy MSM start taking daily PrEP for an indefinite period of time. Thailand recently revised its National Guidelines on HIV/AIDS Treatment and Prevention to recommend treatment for all people living with HIV (PLHIV), regardless of CD4 count. This change alone will increase the number of Thai PLHIV eligible for ART from 246,000 to 407,000 in 2015, an increase of over 65% [11]. The use of limited resources to provide ART to all currently eligible PLHIV may preclude significant expansion of publicly-funded antiretroviral-based HIV prevention strategies at any time in the near future.

Health care personnel and facilities need to prepare for providing PrEP to an at-risk population that could number in the tens or hundreds of thousands. Training needs for health care workers include knowledge about PrEP, risk-reduction counselling skills and importantly the promotion of non-discriminatory attitudes towards PrEP and towards individuals who engage in stigmatized, high-risk behaviour. Experience from PrEP clinical trials and demonstration projects in Thailand can provide examples of best practices to help guide expanded PrEP programming. ART clinics, many of which are already overburdened with high caseloads of PLHIV, may not be the optimal places to provide PrEP services. HIV testing and community-based centres may offer alternate locations to provide PrEP to at-risk populations.

Additional challenges to PrEP implementation include limited knowledge and lack of awareness about PrEP in at-risk communities, ensuring adherence, side effects of the medications, the need for laboratory monitoring and frequent HIV testing, and the risk for the emergence of HIV drug resistance. Demonstration projects using implementation science methodology will be useful to ascertain the most appropriate approaches to the expansion of PrEP services in Thailand and other developing countries [42].

### Recommendations for research agenda

With only limited experience in the implementation of PrEP in Thailand, numerous questions remain, including the appropriate role for PrEP within the national HIV strategy and the most practical and cost-effective ways to provide PrEP services. Which individuals within at-risk groups will benefit the most from the use of PrEP? Is PrEP as efficacious for TGW as it is for other at-risk groups? How to best support adherence, especially among PWID? Who will cover the cost of PrEP drugs in middle-income countries? Will PrEP users be willing to pay for it? What is the role of community-based services and task-shifting to non-physician providers?

These questions and others will need to be addressed as new and ongoing projects in Thailand provide practical experience implementing PrEP. Lessons learned can also be applied to other countries in Asia, which have far less experience with PrEP implementation but have similar challenges in scaling up biomedical HIV prevention methods and face similar HIV epidemics among PWID, MSM and TGW [43].

## Conclusions

Thailand needs new and innovative HIV prevention strategies to address the rapidly evolving HIV epidemic among MSM and TGW. Oral PrEP has been proven to be effective at reducing HIV transmission among MSM, as well as among heterosexuals and PWID. Evidence on the efficacy of PrEP for TGW is lacking and Thailand, with a large population of TGW and a number of facilities that provide sexual reassignment surgery, is well placed to lead the agenda in determining the proper role of PrEP, if any, in this population.

Demonstration projects planned for implementation in 2015 will provide further evidence on the feasibility, acceptability and financial viability of PrEP provision for high-risk MSM and TGW in Thailand. However, PrEP is just one part of a combination HIV prevention strategy. HIV testing and counselling will remain a key entry point to HIV prevention programming, to HIV treatment and care services for those who test HIV positive and to PrEP for those identified as at-risk and eligible. Increased efforts are needed to encourage and attract higher numbers of MSM and TGW to access HIV testing in Thailand.

The BTS demonstrated PrEP efficacy for PWID. Furthermore, the extension phase of the study showed that DOT PrEP can be successfully continued in correctional settings [35]. To demonstrate the continued delivery of this intervention to those who became incarcerated during follow-up is an important outcome of the BTS study with potential implications for addressing the high rate of HIV transmission that has been documented in prison populations [44–47].

Although PrEP has proven efficacy in the clinical trial setting in Thailand and elsewhere, challenges to implementation and questions about the optimal use of TDF-FTC for PrEP remain. It has yet to be scaled up to or evaluated at the level that would be necessary to slow down or halt the epidemic of HIV among MSM in either the developed or developing world. Nonetheless, oral PrEP holds promise as an important component of a combined HIV prevention strategy.

## References

[1] National Statistics Office (2011). The 2010 population and housing census.

[2] Joint United Nations Programme on HIV/AIDS (UNAIDS) (2013). HIV in Asia and the Pacific.

[3] van Griensven F, Phanuphak N, Srithanaviboonchai K (2014). Biomedical HIV prevention research and epidemic control in Thailand: two sides of the same coin. Sex Health.

[4] Royal Thai Army (2013). Results of biannual HIV surveillance among Thai military recruits, 1989–2012.

[5] Ministry of Public Health (2012). Results of sentinel HIV surveillance in Thailand. Round 1–30, 1988–2012.

[6] Rojanapithayakorn W (2006). The 100% condom use programme in Asia. Reprod Health Matters.

[7] Rojanapithayakorn W, Hanenberg R (1996). The 100% condom program in Thailand. AIDS.

[8] The World Bank (2000). Thailand's response to AIDS: building on success, confronting the future.

[9] Punpanich W, Ungchusak K, Detels R (2004). Thailand's response to the HIV epidemic. Yesterday, today and tomorrow. AIDS Educ Prev.

[10] World Health Organization (WHO) (2004). Experiences of the 100% condom use program in selected countries in Asia.

[11] Thai National AIDS Committee (2014). Thailand ending AIDS: 2014 Thailand AIDS response progress report.

[12] Thai Working Group for HIV/AIDS Projections (2010). Projection for HIV/AIDS in Thailand 2010–2030.

[13] van Griensven F, Thanprasertsuk W, Jommaroeng R, Mansergh G, Naorat S, Jenkins RA (2005). Evidence of a previously undocumented epidemic of HIV infection among men who have sex with men in Bangkok, Thailand. AIDS.

[14] van Griensven F, Varangrat A, Wimolsate W, Tappero JW, Sinthuwattanawibol C, McNicholl JM (2006). HIV prevalence among populations of men who have sex with men-Thailand, 2003 and 2005. Morb Mortal Wkly Rep.

[15] Guadamuz TE, Wimonsate W, Varangrat A, Phanuphak P, Jommaroeng R, McNicholl JM (2011). HIV prevalence, risk behavior, hormone use, surgical history and HIV infection among transgender persons in Thailand. AIDS Behav.

[16] van Griensven F, Varangrat A, Wimonsate W, Tanpradech S, Kladsawad K, Chemnasiri T (2010). Trends in HIV prevalence, estimated HIV incidence and risk behavior among men who have sex with men in Bangkok, Thailand, 2003–2007. J Acquir Immune Defic Syndr.

[17] Bureau of Epidemiology (2012). Results of venue-based HIV surveillance among men who have sex with men in Thailand, 2012.

[18] Chariyalertsak S, Kosachunhanan N, Saokhieo P, Songsupa R, Wongthanee A, Chariyalertsak C (2011). HIV incidence, risk factors, and motivation for biomedical intervention among gay, bisexual men, and transgender persons in Northern Thailand. PLoS One.

[19] Nitayaphan S, Benenson M, Sriplienchan S, Morgan P, Eamsila C, Chiu J (2010). ECHO Study (Early Capture HIV Cohort): efficient detection of acute HIV-1 infections in Pattaya, Thailand (RV 217).

[20] Ananworanich J, Chitwarakorn A, Wimonsate W, Varangrat A, Chaikummao S, Sriporn A (2013). HIV and syphilis infection among men who have sex with men – Bangkok, 2005–2011. Morb Mortal Wkly Rep.

[21] Rerks-Ngarm S, Pitisuttithum P, Nitayaphan S, Kaewkungwal J, Chiu J, Paris R (2009). Vaccination with ALVAC and AIDSVAX to prevent HIV-1 infection in Thailand. N Engl J Med.

[22] Grant RM, Lama JR, Anderson PL, McMahan V, Liu AY, Vargas L (2010). Preexposure chemoprophylaxis for HIV prevention in men who have sex with men. N Engl J Med.

[23] Choopanya K, Martin M, Suntharasamai P, Sangkum U, Mock PA, Leethochawalit M (2013). Antiretroviral prophylaxis for HIV infection in injecting drug users in Bangkok, Thailand (the Bangkok Tenofovir Study): a randomised, double-blind, placebo controlled phase 3 trial. Lancet.

[24] HIV Prevention Trials Network (HPTN) (2012). HPTN 067. The ADAPT study.

[25] Microbicide Trials Network (MTN) (2013). MTN-017.

[26] van Griensven F, Ayutthaya P, Wilson E (2013). HIV surveillance and prevention in transgender women. Lancet Infect Dis.

[27] Gooren LJ, Sungkaew T, Giltay EJ, Guadamuz TE (2015). Cross-sex hormone use, functional health and mental well-being among transgender men (Toms) and Transgender Women (Kathoeys) in Thailand. Cult Health Sex.

[28] Sineath RC, Finneran C, Sullivan P, Sanchez T, Smith DK, Griensven F (2013). Knowledge of and interest in using preexposure prophylaxis for HIV prevention among men who have sex with men in Thailand. J Int Assoc Provid AIDS Care.

[29] Yang D, Chariyalertsak C, Wongthanee A, Kawichai S, Yotruean K, Saokhieo P (2013). Acceptability of pre-exposure prophylaxis among men who have sex with men and transgender women in Northern Thailand. PLoS One.

[30] Wheelock A, Eisingerich AB, Ananworanich J, Gomez GB, Hallett TB, Dybul MR (2013). Are Thai MSM willing to take PrEP for HIV prevention? An analysis of attitudes, preferences and acceptance. PLoS One.

[31] Thienkrua W, Todd CS, Chaikummao S, Sukwicha W, Yafant S, Tippanont N (2014). Prevalence and correlates of willingness to participate in a rectal microbicide trial among men who have sex with men in Bangkok. AIDS Care.

[32] Treatment Action Group U.S. Centers for Disease Control and Prevention (CDC) Sponsored HIV Preexposure Prophylaxis (PrEP) Trial among Thai Injection Drug Users Marred by Lack of Response to Community Concerns: Statement of Thai Drug Users Network (TDN), Thai AIDS Treatment Action Group (TTAG), and Treatment Action Group (TAG) [Internet].

[33] WHO (2014). Consolidated guidelines on HIV prevention, diagnosis, treatment, and care for key populations.

[34] Grant RM, Anderson PL, McMahan V, Liu A, Amico KR, Mehrotra M (2014). Uptake of pre-exposure prophylaxis, sexual practices, and HIV incidence in men and transgender women who have sex with men: a cohort study. Lancet Infect Dis.

[35] Martin M, Vanichseni S, Suntharasamai P, Sangkum U, Mock PA, Leethochawalit M Enrollment and preliminary follow-up of injecting drug users receiving pre-exposure prophylaxis in Bangkok.

[36] Study to evaluate the feasibility of community-based test and treat strategies among men who have sex with men and transgender women to increase the uptake of HIV testing and treatment services in Thailand [Internet].

[37] Evaluation of a facility-based test, treat, and prevent HIV program among men who have sex with men and transgender women in Thailand [Internet]. http://www.clinicaltrials.gov/ct2/show/NCT02369887.

[38] The World Bank Thailand [Internet].

[39] National consultation on the strategic use of ARVs – Thailand. Meeting report (2012).

[40] Beyrer C, Baral SD, van Griensven F, Goodreau SM, Chariyalertsak S, Wirtz AL (2012). Global epidemiology of HIV infection in men who have sex with men. Lancet.

[41] Essentials of HIV/AIDS treatment and prevention 2014 [cited 2015 March 25] Thailand [Internet].

[42] World Health Organization (2012). Guidance on oral pre-exposure prophylaxis (PrEP) for serodiscordant couples, men and transgender women who have sex with men at high risk of HIV: recommendations for use in the context of demonstration projects.

[43] Lo YR, Kato M, Phanuphak N, Fujita M, Duc DB, Sopheap S (2014). Challenges and potential barriers to the uptake of antiretroviral-based prevention in Asia and the Pacific region. Sex Health.

[44] Jurgens R, Nowak M, Day M (2011). HIV and incarceration: prisons and detention. J Int AIDS Soc.

[45] Vanichseni S, Kitayaporn D, Mastro TD, Mock PA, Raktham S, Des Jarlais DC (2001). Continued high HIV-1 incidence in a vaccine trial preparatory cohort of injection drug users in Bangkok, Thailand. AIDS.

[46] Thaisri H, Lerwitworapong J, Vongsheree S, Sawanpanyalert P, Chadbanchachai C, Rojanawiwat A (2003). HIV infection and risk factors among Bangkok prisoners, Thailand: a prospective cohort study. BMC Infect Dis.

[47] Buavirat A, Page-Shafer K, van Griensven GJP, Mandel JS, Evans J, Chuaratanaphong J (2003). Risk of prevalent HIV infection associated with incarceration among injecting drug users in Bangkok, Thailand: case-control study. Br Med J.

# Sub article: Pre-exposure prophylaxis for men and transgender women who have sex with men in Brazil: opportunities and challenges

**DOI:** 10.7448/IAS.18.4.20010

**Published:** 2015-07-20

**Authors:** Valdilea G Veloso, Fabio Mesquita, Beatriz Grinsztejn

**Affiliations:** 1 Instituto Nacional de Infectologia Evandro Chagas, Fundação Oswaldo Cruz, Rio de Janeiro, Brasil; 2 Departamento de Doenças Sexualmente Transmissíveis, AIDS e Hepatites Virais do Ministério da Saúde, Brasília, Brasil

**Keywords:** pre-exposure prophylaxis, MSM, TGW, prevention, Brazil, resource-limited setting, health system

## Abstract

**Introduction:**

The World Health Organization recently released guidelines on the use of pre-exposure prophylaxis (PrEP) for prevention of HIV infection among men and transgender women (TGW) who have sex with men based on results of randomized clinical trials. The aim of this commentary is to discuss the opportunities and challenges of incorporating PrEP into the Brazilian continuum of HIV care and prevention for men who have sex with men (MSM) and TGW.

**Discussion:**

Key aspects of the AIDS epidemic among MSM and TGW in Brazil and the comprehensive Brazilian response to the epidemic are presented. The universal access to health care provided through the Brazilian Unified Health System (SUS) and the range of prevention and care services already available countrywide to HIV-positive individuals and at-risk MSM and TGW are identified as the main facilitators for the implementation of PrEP. Limited PrEP awareness among MSM, TGW and health care providers, low HIV testing frequency and low HIV risk perception among MSM and TGW represent the core challenges to be addressed. Data generated by demonstration projects in Brazil will provide an important contribution to PrEP rollout in Brazil.

**Conclusions:**

The implementation of PrEP in Brazil is feasible. A synergistic rollout of treatment as prevention and PrEP will maximize public health and individual benefits of the country's comprehensive response to the AIDS epidemic.

## Introduction

Over the past five years, remarkable progress has been made in the fight against the HIV/AIDS epidemic. Data from randomized clinical trials demonstrating the efficacy and safety of antiretroviral drugs for the prevention of HIV acquisition [1–5] have inspired a renewed sense of optimism that the end of the AIDS era is an attainable goal [6].

Antiretroviral pre-exposure prophylaxis (PrEP), with either daily oral tenofovir disoproxil fumarate (TDF) or daily TDF in combination with emtricitabine, has been shown to be efficacious for HIV-1 prevention for high-risk men who have sex with men (MSM) and transgender women (TGW), heterosexual men and women, discordant heterosexual couples and intravenous drug users [1–4]. Data from the Pre-Exposure Prophylaxis Initiative (iPrEx) study demonstrated that oral PrEP using daily emtricitabine/tenofovir (Truvada^®^) successfully reduced the risk of HIV acquisition among MSM and TGW [1]. Protection was estimated to be over 90% in those with detectable levels of the drug in their blood, with pharmacokinetic modelling suggesting that efficacy reaches 99 and 96% with dosing of seven and four days per week, respectively [7]. Further results from the iPrEX open label extension (iPrEX OLE) reassured that this strategy can be safe and effective, and is well accepted by this population [8].

More recently, two European MSM and TGW oral PrEP clinical trials (IPERGAY and PROUD) halted their randomization phase due to the superior effectiveness of PrEP. Both studies showed 86% effectiveness of Truvada [9, 10]. The IPERGAY's results were of particular interest because this study was designed to evaluate an intermittent PrEP regimen using Truvada (on-demand), with its usage triggered by sexual activity. Currently, the use of PrEP is endorsed by the World Health Organization (WHO), the Centres for Disease Control and Prevention (CDC) and IAS–USA Guidelines [11–13].

The aim of this commentary is to discuss the opportunities and challenges of incorporating PrEP into the continuum of HIV care and prevention for MSM and TGW in Brazil.

## Discussion

### MSM and TGW – the most affected populations in Brazil

Most countries in Latin America have been affected by concentrated HIV/AIDS epidemics, and HIV infection rates in this region have changed little in the past decade, with most of the new HIV cases occurring among MSM [14, 15].

The largest population of HIV-1-positive people in Latin America lives in Brazil. As of 2014, the Ministry of Health (MoH) had registered 757,042 cases of AIDS. The number of people living with HIV/AIDS in the country was estimated to reach 734,000 in 2014. However, it is the young MSM who account for nearly 40% of AIDS cases. Increases of 41.3% (aged 15–19 years) and 25.1% (aged 20–24 years) were observed from 2004 to 2013 [16] (Figure 1).

**Figure 1:** Proportion of all AIDS cases occurring in young men who have sex with men according to age in Brazil from 2004 to 2013. Source: Brazilian Ministry of Health. Department of STDs, AIDS and Viral Hepatitis [16].

Although Brazil has an overall HIV prevalence of roughly 0.6% in the general population (0.4% among women and 0.8% among men) [17], the prevalence among MSM is 14.2% [18], which is three times higher than estimates for female sex workers, double the 5.9% estimated prevalence for drug users and 13 times higher than that for heterosexual men [17]. The prevalence of HIV infection for very young MSM (aged 15–19 years) is 4% (95% CI 1–9%) [19]. Data from three voluntary counselling and testing sites in Rio de Janeiro showed 24.8% (95% CI 19.9–29.7) prevalence among MSM, and conservatively estimated incidence among MSM to be 8.55% per year (95% CI 4.36–12.74) [20].

Although TGW represent a smaller population than MSM, they have extremely elevated HIV infection rates. A meta-analysis across 15 countries (10 were low- and middle-income countries, 5 of which were in Latin America and the Caribbean) estimated an HIV prevalence of 17.7% (95% CI 15.6–19.8) in this population, with an odds ratio of 50.0 (95% CI 26.5–94.3) for HIV infection among TGW versus all adults of reproductive age in low- and middle-income countries [21]. In Brazil, as in other Latin American countries, risks associated with HIV infection among TGW are mainly linked to high rates of sex work, limited formal education, social exclusion and violence. These factors jointly contribute to increased vulnerability and impaired access to care and prevention [22].

Studies in Brazil have shown that unprotected anal intercourse is a frequently reported sexual practice among Brazilian MSM [18, 23]. Nevertheless, a high proportion of MSM classified their risk of acquiring HIV infection as low or did not know how to rate their risk [24]. The HIV epidemic among MSM and TGW in our setting is unabated in these populations with many individuals remaining unaware either of their HIV status and the beneficial services available to them or of effective prevention strategies [15].

### Where does PrEP fit in the Brazilian continuum of HIV prevention and care?

Since the early 1990s, Brazil has implemented a comprehensive HIV prevention and care programme. Built within the Unified Health System (SUS) that provides universal health care to the entire population at no cost at the point of delivery [25], the programme includes voluntary counselling and testing services, combination antiretroviral therapy (cART), viral load and CD4 monitoring and HIV genotyping [19, 26–28]. Condom and lubricant, non-occupational post-exposure prophylaxis (nPEP), treatment for sexually transmitted infections using the syndromic approach, and hepatitis B diagnosis and treatment are also available as part of integral care. This makes it one of the most comprehensive HIV treatment initiatives implemented in a middle-income country [16, 19, 26].

Since December 2013, the Brazilian MoH has adopted the Test & Treat strategy that allows cART to be initiated promptly after HIV diagnosis, regardless of CD4 count, if the patient is willing to be treated [27]. As of December 2014, approximately 400,000 patients were receiving cART in 724 specialized care services established in the country [19].

A high coverage of cART, especially within MSM networks and in the community, is crucial for reducing the spread of the epidemic among MSM. However, the cascade of care in Brazil shows that in 2013, at each level, important percentages of those living with HIV fall out of the care continuum [16]. Of the estimated 734,000 HIV- positive individuals, only 255,000 (33%) achieved an undetectable viral load [16] (Figure 2). Similar results were found when a MSM care cascade was evaluated in Rio de Janeiro, one of the epicentres of the HIV epidemic in Brazil [29].

**Figure 2:** The cascade of HIV care in Brazil in 2013. Source: Brazilian Ministry of Health. Department of STDs, AIDS and Viral Hepatitis [16].

In this context, other prevention strategies, such as nPEP and PrEP targeting HIV-negative high-risk MSM and TGW, could play a critical role in preventing new infections. Since 2010, nPEP has been made available through the SUS [30] and its uptake has been steadily increasing (Figure 3). The addition of PrEP can further contribute to avoiding new infections among these populations and contribute to controlling the HIV epidemic in Brazil. We foresee that PrEP rollout in Brazil will take advantage of the countrywide infrastructure already established. However, critical challenges will have to be addressed.

**Figure 3:** Number of individuals receiving non-occupational post-exposure prophylaxis (nPEP) from 2012 to 2014. Source: Brazilian Ministry of Health. Department of STDs, AIDS and Viral Hepatitis [16].

Significant efforts are needed to increase HIV serostatus awareness and testing frequency among MSM and TGW. In 2010, 54% of MSM participating in the Brazilian National HIV Behavioral Surveillance Study reported having been HIV tested at least once in their life. However, only 19% reported an HIV test in the previous 12 months [19]. Other studies confirmed this finding, showing that less than half of the MSM enrolled had ever been tested for HIV [18, 23], and that of all the men who tested HIV positive, only half were aware of their serostatus [18, 23, 24].

Testing modalities offered in settings outside the traditional health services are critical for increasing access to HIV diagnosis among MSM and TGW. In this regard, building on a long history of partnership with civil society [31, 32], the Brazilian MoH in 2014 launched the initiative, “Viva Melhor Sabendo” (“Living better knowing”), which expanded HIV testing using oral fluid to non-governmental organizations [19]. In addition, mobile HIV testing units were provided to each one of the 27 brazilian federative units to outreach populations that may face barriers to accessing HIV testing in the context of traditional health services. In 2011, among 629 MSM surveyed though the Internet, 47% indicated a preference for home-based testing among several testing options, and up to 90% reported that they would use self-test kits to make choices about unprotected sex with regular and new partners [33]. A menu-based approach that offers different testing modalities, including novel testing strategies, such as self-testing coupled with already available testing options, might support the development of a tailored testing plan for MSM and TGW engaging in PrEP programmes in Brazil.

In the context of PrEP programmes, it is critical to make sure that individuals starting PrEP are HIV negative and remain negative while using it. In case a breakthrough HIV infection occurs, the two drug regimen used in PrEP will not fully suppress HIV replication and may select for resistance. Thus, if acute infection is suspected, PrEP initiation should be delayed until the serostatus is defined to avoid the risk of drug resistance development.

PrEP programmes will have to be coupled with HIV risk management counselling and HIV testing services, where individuals at risk are linked and can have access to PrEP and other prevention options in a stigma-free setting. Within these settings, tools to support risk assessments and adherence are crucial, as is the availability of adequate support for PrEP discontinuation.

### PrEP awareness and willingness in the MSM and TGW community

Understanding awareness and willingness to use PrEP is essential for informing public policy formulation. Evidence suggests that the concept of PrEP is well accepted by MSM; however, it is likely that there are various factors affecting PrEP uptake and adherence that may differ across countries. Surveys on PrEP in the United States, India, South Africa, Thailand, China and Peru showed that 44–92% of MSM were receptive to taking PrEP [34–37].

Awareness and willingness to use PrEP is increasing in Brazil. In 2011, only 22% of 552 MSM who participated in a self-administered web survey using Facebook had heard about the iPrEX study results. However, after a brief explanation about iPrEx and its results, 67.5% said that they were extremely likely or very likely to use daily PrEP [38]. Preliminary results from a study that is being conducted in Rio de Janeiro and São Paulo assessing awareness and willingness among MSM and TGW showed that among 734 men who reported having sex with men within 12 months and were seeking HIV testing, 60% were aware of PrEP and nearly 95% (*n*=695) demonstrated willingness to use PrEP to prevent HIV. Older age, having a steady partner and prior history of HIV testing increased the odds of PrEP awareness [39].

There is very limited data on PrEP awareness and willingness among TGW communities. In a study conducted in Thailand, acceptability of PrEP, defined as individuals who reported being “very likely” to use PrEP, was similar in MSM and transgender groups (around 40%). Correlates of PrEP acceptability among TGW were prior PrEP awareness and having private insurance, suggesting that efforts to increase awareness and accuracy of PrEP understanding and minimizing confusion of PrEP with nPEP and other biomedical HIV prevention and treatment modalities may improve uptake for TGW populations. Also, fear of drug interaction between PrEP and other medicines, particularly female hormones, appeared to be an issue and must be clearly addressed in educational campaigns [40].

Results from a qualitative study to assess health care providers and MSM perspectives on acceptance and feasibility of implementing novel HIV prevention interventions in Brazil showed that although most health care providers were reluctant to engage in new prevention strategies, MSM were very interested in exploring new prevention tools [41]. Increasing PrEP knowledge among potential users and health care providers, especially among physicians, is a key step to facilitating PrEP implementation in our setting.

### Demonstration projects in Brazil

The PrEP Brasil study is a demonstration project (clinical trials.gov NCT 01989611, www.prepbrasil.com.br) designed to evaluate the delivery of PrEP for 450 MSM and TGW for one year. It will generate data to facilitate the decision-making process of incorporating PrEP into the SUS. The project, coordinated by Fiocruz, is ongoing at three sites in Rio de Janeiro (Evandro Chagas National Institute of Infectious Diseases-INI/Fiocruz) and São Paulo (University of São Paulo–USP and São Paulo Referral and Training Center). As of May 2015, the study is fully accrued. Final results are expected by April 2016. As part of the PrEP Brazil project, innovative interventions are being tested to assess their ability to support PrEP users with maintaining treatment adherence and continuing with PrEP usage. PrEP adherence is being supported through the use of text message reminders. In addition, drug concentrations will be measured via plasma and dried blood spot specimens. In addition to the Brazilian National AIDS Program, PrEP Brasil has developed key partnerships with the state AIDS programmes of Rio de Janeiro and São Paulo and two non-governmental organizations, Arco-Iris and Pela VIDDA. The project is jointly funded by the MoH, Fiocruz, and federal and state research funding agencies; Gilead Inc. has donated the study drug (Truvada).

A second demonstration project is scheduled to start by mid-2015 and will enrol 800 MSM, commercial sex workers and drug users across four cities: São Paulo, Porto Alegre, Ribeirão Preto and Fortaleza.

Of note, Truvada for prevention use is not yet approved in Brazil but an application has been filed and is under evaluation by the Brazilian Drug Regulatory Authority.

### Will PrEP be cost-effective in Brazil?

Modelling studies suggest that PrEP can be a cost-effective HIV prevention intervention in developed and developing countries if targeted at individuals at highest risk [37–40]. In Peru, Gomez *et al*. found that cost per DALY averted, assuming the iPrEx profile of adherence (a uniform strategy at a 20% coverage level), ranged from US$1,036 to US$4,254 when considering uncertainty due to PrEP conditional efficacy, which is below the WHO Choosing Interventions That Are Cost-Effective (WHO-CHOICE) threshold for a cost-effective intervention for Peru (2010 per capita GDP of US$5401/DALY) [42]. The WHO-CHOICE considers an intervention to be very cost-effective if its cost is less than the GDP per capita per DALY averted and cost-effective if it costs between one and three times the GDP per capita [41].

Although a PrEP cost-effectiveness model has not yet been developed for Brazil, it is very likely that PrEP would be cost-effective if we consider that the Brazilian and the Peruvian epidemics resemble each other (both have concentrated epidemics with MSM and TGW being most affected), that the model developed by Gomez *et al*. reflects the transmission dynamics between these groups, that the costs and effectiveness of PrEP are similar in Brazil as those estimated for Peru and that Brazil's 2010 per capita GDP was twice that of Peru (US$10,978).

We argue that assuming similar costs and effectiveness in Peru and Brazil is plausible for two reasons. First, regarding PrEP's cost, as the sole procurement agent for ARVs within Brazil, the Brazilian MoH has vast experience in negotiating reasonable pricing strategies from pharmaceutical companies [43] and will likely be able to obtain Truvada at a fair cost that would fit well within the ranges assumed in the Peruvian study. Second, regarding PrEP's effectiveness, the better adherence to Truvada in iPrEX and iPrEX OLE studies observed in the Brazilian sites in comparison with the Lima sites suggests that improved or at least similar levels of effectiveness may be reached in Brazil; this supports cost-effectiveness of PrEP with Truvada in Brazil [43, 44].

The ongoing PrEP demonstration projects and national respondent-driven sampling studies among MSM and TGW will contribute with data to develop a model for PrEP cost-effectiveness analysis that reflects the scenarios in Brazil.

## Conclusions

Since the 1990s, the Brazilian MoH has pushed the envelope with its innovative strategies for HIV prevention, care, treatment and respect for human rights. The success of the Brazilian approach helped demonstrate that universal access to cART is not only an effective treatment strategy but also an efficacious prevention tool [28, 31].

The implementation of PrEP in Brazil is feasible. A synergistic rollout of treatment as prevention and PrEP will maximize public health and individual benefits of the country's comprehensive response to the AIDS epidemic. Intensification of combination prevention strategies at critical points in the HIV transmission cycle is key to achieving the 90–90–90 UNAIDS/WHO targets by 2020 and successfully ending the HIV epidemic in Brazil [45].

## References

[1] Grant RM, Lama JR, Anderson PL, McMahan V, Liu AY, Vargas L (2010). Preexposure chemoprophylaxis for HIV prevention in men who have sex with men. N Engl J Med.

[2] Baeten JM, Donnell D, Ndase P, Mugo NR, Campbell JD, Wangisi J (2012). Antiretroviral prophylaxis for HIV prevention in heterosexual men and women. N Engl J Med.

[3] Thigpen MC, Kebaabetswe PM, Paxton LA, Smith DK, Rose CE, Segolodi TM (2012). Antiretroviral preexposure prophylaxis for heterosexual HIV transmission in Botswana. N Engl J Med.

[4] Choopanya K, Martin M, Suntharasamai P, Sangkum U, Mock PA, Leethochawalit M (2013). Antiretroviral prophylaxis for HIV infection in injecting drug users in Bangkok, Thailand (the Bangkok Tenofovir Study): a randomised, double-blind, placebo-controlled phase 3 trial. Lancet.

[5] Cohen MS, Chen YQ, McCauley M, Gamble T, Hosseinipour MC, Kumarasamy N (2011). Prevention of HIV-1 infection with early antiretroviral therapy. N Engl J Med.

[6] UNAIDS The gap report.

[7] Anderson PL, Glidden DV, Liu A, Buchbinder S, Lama JR, Guanira JV (2012). Emtricitabine-tenofovir concentrations and pre-exposure prophylaxis efficacy in men who have sex with men. Sci Transl Med.

[8] Grant RM, Anderson PL, McMahan V, Liu A, Amico KR, Mehrotra M (2014). Uptake of pre-exposure prophylaxis, sexual practices, and HIV incidence in men and transgender women who have sex with men: a cohort study. Lancet Infect Dis.

[9] Molina J, Capitant C, Spire B, Pialoux G, Chidiac C, Charreau I On demand PrEP with oral TDF-FTC in MSM: results of the ANRS ipergay trial.

[10] McCormack S, Dunn D, Group ObotPS Pragmatic open-label randomised trial of preexposure prophylaxis: the PROUD study.

[11] WHO (2014). Consolidated guidelines on HIV prevention, diagnosis, treatment and care for key populations.

[12] CDC (2011). Pre-exposure prophylaxis (PrEP) [Internet].

[13] Marrazzo JM, del Rio C, Holtgrave DR, Cohen MS, Kalichman SC, Mayer KH (2014). HIV prevention in clinical care settings: 2014 recommendations of the International Antiviral Society-USA Panel. JAMA.

[14] Gouws E, Cuchi P (2012). Focusing the HIV response through estimating the major modes of HIV transmission: a multi-country analysis. Sex Transm Infect.

[15] De Boni R, Veloso VG, Grinsztejn B (2014). Epidemiology of HIV in Latin America and the Caribbean. Curr Opin HIV AIDS.

[16] Departamento de DST/AIDS e Hepatites Virais (2014). Boletim Epidemiologico de AIDS/DST. Ministério da Saúde. Secretaria de Vigilância à Saúde.

[17] Departamento de DST/AIDS e Hepatites Virais (2010). Boletim Epidemiológico de DST/AIDS: Ministério da Saúde. Secretaria de Vigilância à Saúde.

[18] Kerr LR, Mota RS, Kendall C, Pinho Ade A, Mello MB, Guimaraes MD (2013). HIV among MSM in a large middle-income country. AIDS.

[19] UNAIDS (2014). Global AIDS response. Progress peporting. Narrative Report. Brazil [Internet].

[20] de Castro CA, Grinsztejn B, Veloso VG, Bastos FI, Pilotto JH, Morgado MG (2010). Prevalence, estimated HIV-1 incidence and viral diversity among people seeking voluntary counseling and testing services in Rio de Janeiro, Brazil. BMC Infect Dis.

[21] Baral SD, Poteat T, Stromdahl S, Wirtz AL, Guadamuz TE, Beyrer C (2013). Worldwide burden of HIV in transgender women: a systematic review and meta-analysis. Lancet Infect Dis.

[22] Martins TA, Kerr LR, Macena RH, Mota RS, Carneiro KL, Gondim RC (2013). Travestis, an unexplored population at risk of HIV in a large metropolis of northeast Brazil: a respondent-driven sampling survey. AIDS Care.

[23] de Sousa Mascena Veras MA, Calazans GJ, de Almeida Ribeiro MC, de Freitas Oliveira CA, Giovanetti MR, Facchini R (2014). High HIV Prevalence among Men who have sex with men in a time-location sampling survey, São Paulo, Brazil. AIDS Behav.

[24] Rocha GM, Kerr LR, de Brito AM, Dourado I, Guimaraes MD (2013). Unprotected receptive anal intercourse among men who have sex with men in Brazil. AIDS Behav.

[25] Paim J, Travassos C, Almeida C, Bahia L, Macinko J (2011). The Brazilian health system: history, advances, and challenges. Lancet.

[26] Ministério da Saúde (1999). Terapia anti-retroviral e Saúde Pública: Um balanço da experiência brasileira.

[27] Departamento de DST/AIDS e Hepatites Virais (2013). Protocolo Clínico e Diretrizes Terapêuticas para Manejo da Infecção pelo HIV em Adultos. Ministério da Saúde. Secretaria de Vigilância em Saúde.

[28] Nunn AS, da Fonseca EM, Bastos FI, Gruskin S (2009). AIDS treatment in Brazil: impacts and challenges. Health Aff (Millwood).

[29] Castro R, Ribeiro-Alves M, Derrico M, Lemos K, Grangeiro J, Jesus B The MSM HIV care cascade in Rio de Janeiro, Brazil.

[30] Departamento de DST/AIDS e Hepatites Virais (2010). Recomendaç[otilde]es para terapia antirretroviral em adultos infectados pelo HIV-2008; Suplemento III – Tratamento e Prevenção. Ministério da Saúde. Secretaria de Vigilância em Saúde.

[31] Galvao J (2005). Brazil and access to HIV/AIDS drugs: a question of human rights and public health. Am J Public Health.

[32] Nunn A, Dickman S, Nattrass N, Cornwall A, Gruskin S (2012). The impacts of AIDS movements on the policy responses to HIV/AIDS in Brazil and South Africa: a comparative analysis. Glob Public Health.

[33] Lippman SA, Perisse AR, Veloso VG, Sullivan PS, Buchbinder S, Sineath RC (2014). Acceptability of self-conducted home-based HIV testing among men who have sex with men in Brazil: data from an on-line survey. Cad Saude Publica.

[34] Mimiaga MJ, Case P, Johnson CV, Safren SA, Mayer KH (2009). Preexposure antiretroviral prophylaxis attitudes in high-risk Boston area men who report having sex with men: limited knowledge and experience but potential for increased utilization after education. J Acquir Immune Defic Syndr.

[35] Barash EA, Golden M (2010). Awareness and use of HIV pre-exposure prophylaxis among attendees of a seattle gay pride event and sexually transmitted disease clinic. AIDS Patient Care STDS.

[36] Eisingerich AB, Wheelock A, Gomez GB, Garnett GP, Dybul MR, Piot PK (2012). Attitudes and acceptance of oral and parenteral HIV preexposure prophylaxis among potential user groups: a multinational study. PLoS One.

[37] Wheelock A, Eisingerich AB, Ananworanich J, Gomez GB, Hallett TB, Dybul MR (2013). Are Thai MSM willing to take PrEP for HIV prevention? An analysis of attitudes, preferences and acceptance. PLoS One.

[38] Périssé ARS (2013). Knowledge about HIV preventive measures among MSM using internet-based social networks in Brazil.

[39] Hoagland B, Veloso VG, De Boni RB, Madruga JV, Kallas EG, Fernandes NM (2015). Awareness and willingness to take pre-exposure prophylaxis (PrEP) among men who have sex with men and transgender women: preliminary findings from the PrEP Brasil study.

[40] Yang D, Chariyalertsak C, Wongthanee A, Kawichai S, Yotruean K, Saokhieo P (2013). Acceptability of pre-exposure prophylaxis among men who have sex with men and transgender women in Northern Thailand. PLoS One.

[41] Lippman SA, Koester KA, Amico KR, Lama JR, Martinez Fernandes N, Gonzales P (2015). Client and provider perspectives on new HIV prevention tools for MSM in the Americas. PLoS One.

[42] Gomez GB, Borquez A, Case KK, Wheelock A, Vassall A, Hankins C (2013). The cost and impact of scaling up pre-exposure prophylaxis for HIV prevention: a systematic review of cost-effectiveness modelling studies. PLoS Med.

[43] Liu A, Glidden DV, Anderson PL, Amico KR, McMahan V, Mehrotra M (2014). Patterns and correlates of PrEP drug detection among MSM and transgender women in the Global iPrEx Study. J Acquir Immune Defic Syndr.

[44] Glidden DV, Buchbinder SP, Anderson PL, McMahan V, Amico KR, Liu AY (2015). PrEP engagement for HIV prevention: results from the iPrEx open label extension.

[45] UNAIDS 90-90-90 An ambitious treatment target to help end the AIDS epidemic 2014 [Internet].

# Sub article: Antiretroviral pre-exposure prophylaxis implementation in the United States: a work in progress

**DOI:** 10.7448/IAS.18.4.19980

**Published:** 2015-07-20

**Authors:** Kenneth H Mayer, Sybil Hosek, Stephanie Cohen, Albert Liu, Jim Pickett, Mitchell Warren, Douglas Krakower, Robert Grant

**Affiliations:** 1 The Fenway Institute, Fenway Health, Boston, MA, USA; 2 Beth Israel Deaconess Medical Center, Boston, MA, USA; 3 Harvard Medical School, Boston, MA, USA; 4 Stroger Hospital of Cook County/CORE Center, Chicago, IL, USA; 5 San Francisco Department of Health, San Francisco, CA, USA; 6 AIDS Foundation of Chicago, Chicago, IL, USA; 7 AIDS Vaccine Advocacy Coalition, New York, NY, USA; 8 Gladstone Institute, University of California, San Francisco, CA, USA

**Keywords:** PrEP, pre-exposure prophylaxis, tenofovir-emtricitabine

## Abstract

**Introduction:**

After the initial approval of the use of tenofovir disoproxil fumarate-emtricitabine (TDF/FTC) by the US Food and Drug Administration in 2012 for anti-HIV pre-exposure prophylaxis (PrEP), uptake was initially limited, but more recent community surveys and expert opinion suggest wider acceptance in some key populations.

**Discussion:**

Demonstration projects are underway to determine the best practices in the United States to identify at-risk individuals in primary care and sexually transmitted disease clinics who could benefit from PrEP. Studies of PrEP in combination with behavioural interventions are being evaluated. Studies to evaluate the use of PrEP by HIV-uninfected women in HIV-discordant couples interested in safe conception are also getting underway. The optimal deployment of PrEP as part of a comprehensive national HIV/AIDS strategy in the United States has been limited by lack of knowledge among some at-risk people and by some medical providers indicating that they do not feel sufficiently knowledgeable and comfortable in prescribing PrEP. Studies are underway to determine how to assist busy clinicians to determine which of their patients could benefit from PrEP. Although most federal health insurance programmes will cover most of the costs associated with PrEP, underinsured patients in states that have not enacted health reform face additional challenges in paying for PrEP medication and appropriate clinical monitoring.

**Conclusions:**

PrEP implementation in the United States is a work in progress, with increasing awareness and uptake among some individuals in key populations.

## Introduction

### From clinical trials to PrEP approval

In 2010 to 2011, the first data from pre-exposure prophylaxis (PrEP) efficacy trials were reported, demonstrating that oral tenofovir disoproxil fumarate-emtricitabine (TDF/FTC) could protect against HIV acquisition. The first demonstration of PrEP efficacy was in the iPrEx trial, which enrolled men who have sex with men (MSM) in Latin America, the United States, South Africa and Thailand [1]. This study, coupled with the demonstration of efficacy in African heterosexuals [2, 3], led to the approval of the use of TDF/FTC for chemoprophylaxis by the Food and Drug Administration in the summer of 2012 and to formal PrEP guidelines issued by the US Centers of Disease Control and Prevention (CDC) [4].

Despite the initial reports of PrEP efficacy, concerns were raised because of the less-than-optimal adherence in iPrEx (approximately 51% had detectable drug levels in their blood) and two PrEP studies in African women that did not demonstrate protection [5, 6]. It was thought that PrEP might not produce a meaningful public health benefit because of “real-world” problems achieving optimal adherence. Fewer than 200 of the 2499 participants in iPrEx were American, so PrEP efficacy data in the United States were limited. However, subsequent American demonstration projects have suggested that, when individuals use open-label PrEP on a voluntary basis, adherence may be better, because users self-select to use PrEP to protect themselves against HIV (Table 1).

**Table 1:** Ongoing and planned PrEP trials and demonstration projects, as of November 2014

Trial/project	Sponsor/funder	Type/Category	Location	Population	Design/key questions	Status
**The Demo Project**	National Institute of Allergy and Infectious Diseases of the NIH	Demonstration Project	US (Miami, FL; San Francisco, CA; and Washington, DC)	MSM and transgender women	Assesses uptake, acceptability, safety and feasibility of once-daily TDF/FTC as PrEP in 600 MSM (300 in San Francisco; 200 in Miami; 100 in Washington)	Ongoing; expected completion date January 2015
**East Bay Consortium/CRUSH (Connecting Resources for Urban Sexual Health)**	California HIV/AIDS Research Program of the University of California	Demonstration Project	US (East Bay, CA)	Young MSM of colour	Testing and linking young MSM of colour to sexual health services; enhance engagement and retention for HIV-positive young MSM of colour; and retain HIV-negative young MSM of colour in sexual health services, including PrEP	Ongoing; started in December 2012
**LAC PATH PrEP Demo Project**	California HIV/AIDS Research Program of the University of California; LA County HIV & STD Program; Los Angeles Gay and Lesbian Center; OASIS Clinic; AIDS Project LA; UCLA	Demonstration Project	US (Los Angeles, CA)	MSM	Evaluates a customized prevention package that may include PrEP Enrolling 375 high-risk MSM and transgender women	Ongoing; expected completion date of May 2017
**California Collaborative Treatment Group Consortium/ALERT (Active Linkage, Engagement and Retention to Reduce HIV)**	California HIV/AIDS Research Program of the University of California, San Diego County HIV, STD, and Hepatitis Branch and the Long Beach Health and Human Services Agency	Demonstration Project	US (Long Beach, Los Angeles and San Diego, CA)	MSM	Evaluates whether a text messaging-based adherence intervention can improve adherence to the PrEP medication. Enrolling 400 high-risk MSM randomized to receive daily TDF/FTC as PrEP	Ongoing; expected completion date October 2015
**SPARK Project NYC**	HART and Callen-Lorde Community Health Center, funded by the National Institute on Alcohol Abuse and Alcoholism	Demonstration Project	US (New York)	MSM and transgender women	Evaluates a comprehensive prevention package that includes PrEP and examines social and behavioural factors associated with disparities in access to prevention and care services among gay, bisexual and other men who have sex with men that might impact PrEP implementation programs	Ongoing; started October 2013. Expected completion of July 2017
**Project PrEPare (Adolescents 18–22)** **Project PrEPare (Adolescents 15–17)**	Adolescent Medicine Trials Network for HIV/AIDS Interventions (ATN); funded by NICHD, NIDA, NIMH	Open-Label Demonstration Project and Phase II Safety Study	US (Baltimore; Boston; Bronx, NY; Chicago; Washington, DC; Denver; Detroit; Houston; Los Angeles; Memphis; Miami; New Orleans; Philadelphia; Tampa)	MSM MSM	Explores the safety, acceptability and feasibility of PrEP among young men who have sex with men (YMSM) who are at risk for HIV infection. Enrolling 300 HIV-uninfected YMSM	Ongoing, started November 2012; expected completion November 2015 Ongoing; expected completion March 2016
**HPTN 073**	HPTN; funded by NIAID/NIH	Open-Label Demonstration Project	US (Los Angeles, CA; Washington, DC; Chapel Hill, NC]	MSM	Assesses the initiation, acceptability, safety and feasibility of PrEP for Black MSM (BMSM); subset of participants will be recruited for qualitative interviews about PrEP facilitators and barriers. Enrolling a total of 225 participants	Ongoing; started July 2013
**CDC Foundation Demonstration Project**	Funding pending	Demonstration Project	US	MSM and heterosexual women	Proposed to evaluate real-world PrEP use in MSM and heterosexual women at risk of HIV infection in health clinic settings, potentially in 1200 participants	Start date pending funding
**iPrEx OLE**	Sponsored/funded by DAIDS/NIH, through a grant to the Gladstone Institutes.	Open-label extension	Brazil, Peru, Ecuador, South Africa, Thailand, US	MSM	Continuation of the iPrEx study designed to provide additional information about the safety of PrEP and the behaviour of people taking PrEP over a longer term	Completed. Results announced July 2014

*Source*: Donaldson E, Grant D and Warren M. Ongoing and Planned US PrEP Trials and Demonstration Projects. Available from: www.avac.org/prevention-option/prep/usdemoprojects [cited 2014 Dec 1].

## Demonstration projects

In order to assess the impact of PrEP after participants learned that it was effective, iPrEx participants were offered access to medication through an open-label extension (iPrEx OLE) protocol [7]. Approximately 65% of the original participants in iPrEx and 68% of participants in the Adolescent Trials Network (ATN) protocol ATN 082 [8] and an earlier CDC safety study [9] who were eligible participated in the iPrEx OLE study. Participants were asked to provide written informed consent and were offered PrEP or ongoing observation without medication at the start of the iPrEx OLE. All participants came in for HIV testing and counselling at quarterly intervals. Most of those (72%) who entered the iPrEx OLE study elected to start PrEP right away and 6% more started using PrEP sometime after enrolment. People were more likely to enrol in iPrEx OLE if they had a history of condomless anal intercourse and/or sexually transmitted disease (STD), suggesting that the Open Label Extension was attractive to those who might benefit most. The majority of participants who elected to use PrEP had detectable drug in the blood when periodically screened. Among participants whose drug levels were consistent with taking TDF/FTC four or more times a week, no seroconversions occurred, compared to an incidence rate of 2.6% for those iPrEx OLE participants who elected not to take PrEP.

The first post-iPrEx US PrEP demonstration project was conducted at STD clinics in San Francisco and Miami and a community health centre in Washington, DC [10, 11]. Six hundred individuals who were PrEP-naïve were recruited via local media and community venues. These individuals were asked to provide written informed consent prior to the initiation of PrEP, so their experiences could be carefully monitored over the course of the subsequent year. The majority of the participants were white, but 7.2% were black and 1.3% were transgender. The study found that there was a good deal of community interest in PrEP at the sites. Among 90 participants whose blood was sampled at week 24, 90% had tenofovir levels consistent with taking at least four doses per week (97% in Washington, DC, 93% in San Francisco and 81% in Miami). Pharmacological modelling studies suggest that these drug levels correlate with a high level of protection [12]. Other demonstration studies have gotten underway in Southern California, enrolling participants in STD clinics and HIV specialty care centres. One of the California studies has included behavioural counselling and drug-level assessment to enhance adherence [13]. Individuals whose drug levels were found to be low received additional counselling. However, there was little need for enhanced counselling, because the majority of participants were highly adherent.

Studies elsewhere are underway to develop other approaches to facilitate PrEP adherence. A team working at Boston's Fenway Health has tested PrEP support tools based on Lifesteps, an evidence-based protocol developed to improve adherence for HIV-infected individuals [14]. In a pilot study funded by the US National Institutes of Health (NIH), participants found the PrEP intervention, which includes four weekly counselling sessions delivered by a nurse, to be highly acceptable and 84% had drug levels consistent with daily PrEP use at six months [15]. Another study is evaluating the use of a mobile health (mHealth) strategy to support PrEP adherence [16] by adapting an SMS-based intervention previously shown to increase ARV adherence and virologic suppression rates in HIV-infected individuals [17]. Gilead Sciences, the developer of Truvada™, has supported several demonstration projects in the United States and elsewhere. PrEP demonstration studies are underway in several southern cities with high rates of new HIV infections, such as Houston, TX, and Jackson, MI, and smaller cities on the East Coast, such as Providence, RI.

## Focused population studies

Although internationally the iPrEx Study included a substantial number of younger MSM, there was limited enrolment of the most vulnerable youth in the United States, young black and Latino MSM. The ATN conducted a PrEP feasibility study in Chicago, which found that youth were interested in taking PrEP, but overall adherence was about 50% [8]. The ATN is now studying whether PrEP adherence in two parallel studies can be improved with either of two evidence-based HIV-prevention interventions, one individualized and the other a group intervention (ATN 110 for participants aged 18 to 22 years old and ATN 113 for those aged 15 to 17 years old). Almost half (49%) of the participants in ATN 110 are black, 27% are Latino and 5% are transgender women; 33% of the participants in ATN 113 are black and 49% Latino.

Although black MSM in the United States are disproportionately affected by HIV [18–21], they were not the primary focus of prior studies. Black MSM do not engage in condomless anal sex more often than other MSM, but, because of assortative mixing, poverty and other adverse social consequences, black MSM remain at great risk for HIV acquisition, and many would be appropriate candidates for PrEP [18–21]. The HPTN 073 study is a demonstration project that has recruited 225 black MSM in Washington, DC, North Carolina and Los Angeles. The study has enrolled 225 black MSM, who have been offered clinical monitoring and care coordination with or without PrEP. The coordinated clinical care is designed to address unmet social and structural needs (e.g. health insurance, stable housing) as well as behavioural health concerns (e.g. depression or substance use), any of which might impede PrEP adherence. The educational materials and counselling protocols have been culturally tailored by a team of black MSM researchers. The study is still underway but, encouragingly, the majority of enrolees chose to initiate PrEP.

Although more than a quarter of new US HIV infections occur in heterosexual women, identifying specific women who might most benefit from PrEP has been challenging [22]. Many women who become HIV-infected may be unaware of their partner's serostatus; because of power dynamics in their primary relationship, they may not be able to negotiate safer sex with their partners. Because black MSM are a minority among MSM, strategies designed to recruit those at greatest risk for HIV are straightforward, for example, focusing on social venues and media used by black MSM. Identifying at-risk women is more challenging, because there are millions of American women who live in high prevalence communities, but most are not likely to become HIV-infected by their primary partners. A recent study designed to assess the predictors of HIV incidence among at-risk women (HPTN 064, the “ISIS” study) found an annualized HIV incidence of 0.25% [23]. This low level of HIV incidence would make it very difficult to conduct an efficacy trial to evaluate the benefit of PrEP for high-risk US women, because thousands would need to be enrolled in order to demonstrate efficacy. Given the low HIV incidence in US women, concerns have been raised about the chronic use of PrEP (given costs and toxicities) to prevent the rare likelihood that individual women would become HIV-infected. However, women who are in an HIV-discordant relationship could clearly benefit from regular use of PrEP. Some have argued that if the HIV-infected partner initiates antiretroviral therapy and is stably virologically suppressed for at least six months, the female partner does not need to use PrEP. However, this strategy would rely on the woman having perfect knowledge that her partner was adherent and virologically undetectable. Particularly for couples that are contemplating having children, for whom an HIV transmission to the foetus or infant would be unacceptable, “PrEPception” may offer some major advantages. A multicentre group of women's health investigators are offering HIV-discordant couples a menu of options, including virologic suppression of the infected partner, PrEP for the HIV-negative female partner, as well as assisted reproduction. This study will not be powered to assess the efficacy of any one strategy but will provide invaluable insights about the acceptability of the different approaches to protecting child-bearing women who have HIV-infected partners.

Transgender people are highly affected by HIV [24]. Because a relatively low percent of iPrEx participants were transgender women, there are insufficient *data* regarding PrEP safety, acceptability and efficacy for them. The iPrEx OLE study found that TDF/FTC concentrations were, on average, lower among transgender women compared with MSM [7]. Although suboptimal medication adherence is thought to explain some of the differences, the possibility that drug-drug interactions of exogenous feminizing sex hormones could alter intercellular FTC or TDF concentrations is under study. Further studies of PrEP for transgender women are needed.

## Community responses

In a manner very analogous to the rollout of hormonal contraception a half century ago, the responses to the proof of efficacy of PrEP have been quite mixed [25]. Many gay and reproductive rights activists have applauded the advent of this new prevention option; however, other individuals have seen PrEP as a preventive intervention that is fraught with danger. Some American gay community leaders have issued ads and pronouncements expressing concerns about PrEP, unsubstantiated by available data. The concerns have included questions about whether PrEP will increase behavioural disinhibition, resulting in new HIV transmissions, whether it will promote the development of resistant strains of HIV, and/or increase rates of STDs. Concerns have also been raised regarding rates of toxicity of the medications, because side effects may be less acceptable for individuals who are otherwise healthy than for people at risk of developing AIDS without medication. In response to some of these criticisms, several organizations that focus on MSM health, sexual and reproductive health rights and community education have developed public information campaigns, often conducted on the internet, to correct misinformation (Table 2). These organizations include the AIDS Foundation of Chicago, the San Francisco AIDS Foundation, Fenway Health and Project Inform. These groups have also attempted to educate at-risk populations and providers through opinion pieces in local and national media. Most recently, the largest and longest running national coalition of community-based HIV/AIDS organizations – the AIDS United Public Policy Committee – issued a call to end the debate about PrEP and shift the national focus to scale-up of this intervention [26].

**Table 2:** Useful US-focused PrEP resources

Organization	Webpage
AIDS Foundation of Chicago	www.myprepexperience.blogspot.com/
Project Inform	www.projectinform.org/prep/
San Francisco AIDS Foundation	www.prepfacts.org
The Fenway Institute	www.thefenwayinstitute.org/prepinfo/
The US Centers for Disease Control and Prevention	www.cdc.gov/hiv/prevention/research/prep
The AIDS Vaccine Advocacy Coalition	www.avac.org

Several individuals who believe that wider access to PrEP will provide more options for individuals to make safer choices have noted that some of the controversy in the gay and mainstream media has actually increased the knowledge base of individuals who might benefit from PrEP. The dissemination of correct information remains extremely important: recent surveys in one of the larger social networking sites, Manhunt, found in early 2014 that only 3.1% of almost 9000 MSM respondents had used PrEP and that substantial numbers had not heard of it [27]. Additionally, a study by Gilead Sciences with one of the largest national retail pharmacy chains found that there were only around 2,500 individuals who had received PrEP, and about half of them were women [28]. More recent surveys and discussions with key opinion leaders suggest that PrEP utilization may be growing, but the scale of PrEP utilization among those who could benefit the most remains unclear.

## Provider issues

Several studies have found that one of the biggest barriers to the provision of PrEP are the reticence of health-care providers, some of whom have expressed concerns about behavioural disinhibition, risk compensation, costs and potential toxicities with PrEP [29–32], particularly in communities where the number of persons needing to be placed on PrEP may be high relative to the number of HIV infections averted. Given that many providers in general practice do not routinely ask their patients about their sexual orientation or gender identity [33, 34], conversations about the appropriateness of PrEP may not be easily undertaken. Several organizations, such as Fenway Health, have developed provider education campaigns, including monograph and webinars (www.lgbthealtheducation.org), that supply key PrEP information for providers and potential consumers.

Concerns have been raised that insurers would not pay for PrEP. Because the United States does not have an integrated health-care system and health is primarily regulated by the states, there have been a variety of responses by regulatory authorities to PrEP. For the most part, private insurance companies will cover the cost of PrEP, but, depending on the type of insurance an individual has, co-payments as high as $100 per month may be expected, thereby eliminating PrEP access for individuals who have modest incomes and inadequate insurance. Gilead Sciences maintains a patient assistance program, which has been beneficial to individuals with very limited economic means, but it has left gaps for others who have high co-payments, but whose salaries are above the threshold for these programs [35]. In states that have accepted the expansion of Medicaid, as part of the implementation of the Affordable Care Act, few residual barriers exist for support for PrEP implementation. In addition to the cost of the actual medication, which can be close to $15,000 per year if paid out of pocket, there are other attendant medical costs, since best practices mandate that PrEP users should be routinely counselled and tested for HIV and bacterial STDs on at least a quarterly basis, as well as having their renal function monitored.

## Conclusions about PrEP in the United States

Several studies are underway in the United States, as well as internationally, that may have an impact on how PrEP is delivered over the next few years [36]. In October 2014, the British PROUD open-label oral TDF/FTC PrEP demonstration project (www.proud.mrc.ac.uk/) determined that MSM assigned to receive PrEP had an 86% decrease in their risk of becoming HIV-infected compared to participants assigned to the waiting-list condition [37]. The importance of this study is that it is the first demonstration project to clearly show that real-world access to PrEP can significantly decrease HIV incidence in MSM.

Because maintenance of high levels of adherence has been a challenge for many earlier trial participants – as well as to address concerns that have been raised about resistance, cost and drug toxicity – studies are underway to assess whether more parsimonious dosing schedules may be protective. In the iPrEx Study, a retrospective analysis of drug levels found that individuals who took the medication at a frequency of approximately four times per week had a comparable level of protection to those who took the medication on a daily basis [13]. A study conducted in France and Quebec, iPERGAY, is a placebo-controlled trial evaluating pericoital oral TDF/FTC prophylaxis in MSM. This trial has found that MSM assigned to receive active medication were 86% less likely to become HIV-infected than those assigned to the placebo condition [38]. The US CDC and other public health authorities have not endorsed less-than-daily PrEP dosing at this point, because there are data from multiple trials supporting this approach, but as additional data regarding event-driven, pericoital oral PrEP become available, recommendations could change. Other studies that may influence how PrEP is prescribed include studies of different oral medications (e.g. maraviroc) and different delivery systems (e.g. injections and vaginal rings).

Although some of the key research showing the efficacy of PrEP was conducted in the United States, and US regulatory authorities have approved its use for at-risk individuals engaging in condomless sex, uptake has been slower than expected by some, given that about 50,000 Americans become HIV-infected annually. On the other hand, some innovations may take more than a decade to become more widely used. Some critics have raised concerns about the unintended consequences of PrEP use, including risk compensation, selection for drug resistance, unappreciated drug toxicity and cost. Despite these anxieties, none of the studies to date have shown that these concerns are substantially warranted, though ongoing surveillance and monitoring is essential. Although previous studies have suggested slow uptake of PrEP [39], more recent data suggest that there is increasing interest in PrEP in some urban centres where there is access to informed providers [40]. Clearly, optimal implementation of PrEP will require further refinements in both community and provider education. The challenges are daunting, but PrEP has the opportunity to be part of a response that can help arrest the continued spread of HIV in the United States and around the world.

## References

[1] Grant RM, Lama JR, Anderson PL, McMahan V, Liu AY, Vargas L (2010). Preexposure chemoprophylaxis for HIV prevention in men who have sex with men. N Engl J Med.

[2] Baeten JM, Donnell D, Ndase P, Mugo NR, Campbell JD, Wangisi J (2012). Antiretroviral prophylaxis for HIV prevention in heterosexual men and women. N Engl J Med.

[3] Thigpen MC, Kebaabetswe PM, Paxton LA, Smith DK, Rose CE, Segolodi TM (2012). Antiretroviral preexposure prophylaxis for heterosexual HIV transmission in Botswana. N Engl J Med.

[4] US Public Health Service Preexposure prophylaxis for the prevention of HIV infection in the United States – 2014 clinical practice guideline [cited 2014 Oct 5].

[5] Marrazzo JM, Ramjee G, Nair GB, Palanee T, Mkhiza B, Nakabiito C Pre-exposure prophylaxis for HIV in women: daily oral tenofovir, oral tenofovir/emtricitabine, or vaginal tenofovir gel in the VOICE study (MTN 003).

[6] Van Damme L, Corneli A, Ahmed K, Agot K, Lombaard J, Kapiga S (2012). Preexposure prophylaxis for HIV infection among African women. N Engl J Med.

[7] Grant RM, Anderson PL, McMahan V, Liu A, Amico KR, Mehrotra M (2014). Uptake of pre-exposure prophylaxis, sexual practices, and HIV incidence in men and transgender women who have sex with men: a cohort study. Lancet Infect Dis.

[8] Hosek SG, Siberry G, Bell M, Lally M, Kapogiannis B, Green K (2013). The acceptability and feasibility of an HIV preexposure prophylaxis (PrEP) trial with young men who have sex with men. J Acquir Immune Defic Syndr.

[9] Grohskopf LA, Chillag KL, Gvetadze R, Liu AY, Thompson M, Mayer KH (2013). Randomized trial of clinical safety of daily oral tenofovir disoproxil fumarate among HIV-uninfected men who have sex with men in the United States. J Acquir Immune Defic Syndr.

[10] Liu A, Cohen S, Follansbee S, Cohan D, Weber S, Sachdev D (2014). Early experiences implementing pre-exposure prophylaxis (PrEP) for HIV prevention in San Francisco. PLoS Med.

[11] Cohen SE, Vittinghoff E, Anderson PL, Doblecki-Lewis S, Bacon O, Chege W (2014). Implementation of PrEP in STD clinics: high uptake and drug detection among MSM in the demonstration project.

[12] Anderson PL, Glidden DV, Liu A, Buchbinder S, Lama JR, Guanira JV (2012). Emtricitabine-tenofovir concentrations and pre-exposure prophylaxis efficacy in men who have sex with men. Sci Transl Med.

[13] Amico KR, Psaros C, Safren S, Kofron R, Flynn R, Bolan R (2014). Real time plasma TFV levels to support adherence in a pre-exposure prophylaxis demonstration project.

[14] Safren SA, Otto MW, Worth JL, Salomon E, Johnson W, Mayer K (2001). Two strategies to increase adherence to HIV antiretroviral medication: life-steps and medication monitoring. Behav Res Ther.

[15] Mayer K, Safren S, Haberer J, Elsesser S, Clarke W, Hendrix C Project PrEPare: high levels of medication adherence with continued condomless sex in U.S. men who have sex with men in an oral PrEP adherence trial. HIV Research for Prevention 2014.

[16] Liu A, Stojanovski K, Lester R, Amico KR, McMahan V (2013). Developing and implementing a mobile health (mHealth) adherence support system for HIV-uninfected men who have sex with men (MSM) taking pre-exposure prophylaxis (PrEP): the iText Study.

[17] Lester RT, Ritvo P, Mills EJ, Kariri A, Karanja S, Chung MH (2010). Effects of a mobile phone short message service on antiretroviral treatment adherence in Kenya (WelTel Kenya1): a randomised trial. Lancet.

[18] Koblin BA, Mayer KH, Eshleman SH, Wang L, Mannheimer S, del Rio C (2013). Correlates of HIV acquisition in a cohort of Black men who have sex with men in the United States: HIV prevention trials network (HPTN) 061. PLoS One.

[19] Millett GA, Peterson JL, Flores SA, Hart TA, Jeffries WL, Wilson PA (2012). Comparisons of disparities and risks of HIV infection in black and other men who have sex with men in Canada, UK, and USA: a meta-analysis. Lancet.

[20] Raymond HF, McFarland W (2009). Racial mixing and HIV risk among men who have sex with men. AIDS Behav.

[21] Prejean J, Song R, Hernandez A, Ziebell R, Green T, Walker F (2011). Estimated HIV incidence in the United States, 2006–2009. PLoS One.

[22] Hodder SL, Justman J, Hughes JP, Wang J, Haley DF, Adimora AA (2013). HIV acquisition among women from selected areas of the United States: a cohort study. Ann Intern Med.

[23] Eshleman SH, Hughes JP, Laeyendecker O, Wang J, Brookmeyer R, Johnson-Lewis L (2013). Use of a multifaceted approach to analyze HIV incidence in a cohort study of women in the United States: HIV Prevention Trials Network 064 Study. J Infect Dis.

[24] Baral SD, Poteat T, Stromdahl S, Wirtz AL, Guadamuz TE, Beyrer C (2013). Worldwide burden of HIV in transgender women: a systematic review and meta-analysis. Lancet Infect Dis.

[25] Myers JE, Sepkowitz KA (2013). A pill for HIV prevention: deja vu all over again?. Clin Infect Dis.

[26] (2014). Leading HIV/AIDS groups endorse CDC HIV PrEP guidelines; reiterate that PrEP is a powerful, additional tool in the AIDS response.

[27] Mayer KH, Oldenburg CE, Novak DS, Krakower DS, Mimiaga MJ (2014). Differences in PrEP knowledge and use in U.S. MSM users of a popular sexual networking site surveyed in August 2013 and January 2014.

[28] Giler RM, Magnuson D, Ng L, Bush S, Rawlings KM (2014). Changes in the characteristics of PrEP users in the US before and after Truvada for PrEP approval.

[29] Krakower D, Oldenburg CE, Mitty JA, Wilson I, Kurth A, Maloney K (2014). New England healthcare providers’ perceptions, knowledge and practices regarding the use of antiretrovirals for prevention.

[30] Karris MY, Beekmann SE, Mehta SR, Anderson CM, Polgreen PM (2014). Are we prepped for preexposure prophylaxis (PrEP)? Provider opinions on the real-world use of PrEP in the United States and Canada. Clin Infect Dis.

[31] Tellalian D, Maznavi K, Bredeek UF, Hardy WD (2013). Pre-exposure prophylaxis (PrEP) for HIV infection: results of a survey of HIV healthcare providers evaluating their knowledge, attitudes, and prescribing practices. AIDS Patient Care STDs.

[32] Krakower D, Ware N, Mitty JA, Maloney K, Mayer KH (2014). HIV providers; perceived barriers and facilitators to implementing pre-exposure prophylaxis in care settings: a qualitative study. AIDS Behav.

[33] Sherman MD, Kauth MR, Shipherd JC, Street RL (2014). Provider beliefs and practices about assessing sexual orientation in two veterans health affairs hospitals. LGBT Health.

[34] Petroll AE, Mosack KE (2011). Physician awareness of sexual orientation and preventive health recommendations to men who have sex with men. Sex Transm Dis.

[35] Gilead Sciences Paying for Truvada.

[36] AIDS Vaccine Advocacy Coalition.

[37] McCormack S, Dunn D (2015). Pragmatic open-label randomised trial of preexposure prophylaxis: the PROUD STUDY.

[38] Molina J-M, Capitant C, Spire B (2015). On demand PrEP with oral TDF-FTC in MSM: results of the ANRS Ipergay trial.

[39] Brooks RA, Kaplan RL, Lieber E, Landovitz RJ, Lee SJ, Leibowitz AA (2011). Motivators, concerns, and barriers to adoption of preexposure prophylaxis for HIV prevention among gay and bisexual men in HIV-serodiscordant male relationships. AIDS Care.

[40] Mayer KH, Levine K, Grasso C, Krakower DS, Mimiaga M (2015). Recent increases in PreP utilization among men who have sex with men in a Boston community health center 2011–2014: transition from research to clinical practice.

# Sub article: Rethinking HIV prevention to prepare for oral PrEP implementation for young African women

**DOI:** 10.7448/IAS.18.4.20227

**Published:** 2015-07-20

**Authors:** Connie L Celum, Sinead Delany-Moretlwe, Margaret McConnell, Heidi van Rooyen, Linda-Gail Bekker, Ann Kurth, Elizabeth Bukusi, Chris Desmond, Jennifer Morton, Jared M Baeten

**Affiliations:** 1 Department of Global Health, University of Washington Seattle, WA, USA; 2 Department of Medicine, University of Washington Seattle, WA, USA; 3 Department of Epidemiology, University of Washington Seattle, WA, USA; 4 Wits Reproductive Health and HIV Institute, University of the Witwatersrand, Johannesburg, South Africa; 5 Department of Global Health and Population, Harvard T.H. Chan School of Public Health Boston, MA, USA; 6 Human Sciences Research Council, Durban, South Africa; 7 The Desmond Tutu HIV Centre, University of Cape Town, Cape Town, South Africa; 8 College of Nursing, New York University New York, NY, USA; 9 Kenya Medical Research Institute, Nairobi, Kenya

**Keywords:** HIV, prevention, pre-exposure prophylaxis, Africa, women

## Abstract

**Introduction:**

HIV incidence remains high among young women in sub-Saharan Africa in spite of scale-up of HIV testing, behavioural interventions, antiretroviral treatment and medical male circumcision. There is a critical need to critique past approaches and learn about the most effective implementation of evidence-based HIV prevention strategies, particularly emerging interventions such as pre-exposure prophylaxis (PrEP).

**Discussion:**

Women in sub-Saharan Africa are at increased risk of HIV during adolescence and into their 20s, in part due to contextual factors including gender norms and relationship dynamics, and limited access to reproductive and sexual health services. We reviewed behavioural, behavioural economic and biomedical approaches to HIV prevention for young African women, with a particular focus on the barriers, opportunities and implications for implementing PrEP in this group. Behavioural interventions have had limited impact in part due to not effectively addressing the context, broader sexual norms and expectations, and structural factors that increase risk and vulnerability. Of biomedical HIV prevention strategies that have been tested, daily oral PrEP has the greatest evidence for protection, although adherence was low in two placebo-controlled trials in young African women. Given high efficacy and effectiveness in other populations, demonstration projects of open-label PrEP in young African women are needed to determine the most effective delivery models and whether women at substantial risk are motivated and able to use oral PrEP with sufficient adherence to achieve HIV prevention benefits.

**Conclusions:**

Social marketing, adherence support and behavioural economic interventions should be evaluated as part of PrEP demonstration projects among young African women in terms of their effectiveness in increasing demand and optimizing uptake and effective use of PrEP. Lessons learned through evaluations of implementation strategies for delivering oral PrEP, a first-generation biomedical HIV prevention product, will inform development of new and less user-dependent PrEP formulations and delivery of an expanding choice of prevention options in HIV prevention programmes for young African women.

## Introduction: HIV epidemiology and prevention in young women in Africa

Young women aged below 25 account for three of the almost four million young people in sub-Saharan Africa who are living with HIV and have one of the highest HIV incidence rates globally [1]. New prevention tools including community-wide HIV testing, antiretroviral treatment (ART) as prevention, and voluntary medical male circumcision have shown tremendous promise and implementation is progressing. However, women, particularly young women, have not maximally benefited from these interventions because they are not directly under women's control. Women are also are at increased risk because their male sexual partners are less likely to be HIV tested and know their HIV serostatus [2], and their HIV infected partners are less likely to have viral suppression due to late ART initiation and higher drop-off in the HIV care continuum [3].

Women are particularly vulnerable to HIV during adolescence and their early 20s due to biological, behavioural and structural factors. Biological factors that increase HIV susceptibility among women include co-infection with sexually transmitted infections (STIs) [4–7], genital inflammation [8], and their male partners’ circumcision status [9, 10] and viral load [11, 12]. Hormonal influences such as pregnancy [13] and injectable contraception [14] have also been shown in observational studies to increase HIV risk, although the data are not conclusive. Individual-level behavioural factors that contribute to the increased vulnerability of young African women to HIV infection include younger age at sexual debut [15], older sexual partners [16, 17], concurrent sexual partners [18, 19], lack of consistent condom use [20], interpersonal physical and sexual violence [21, 22], and alcohol abuse [23]. Contextual factors such as poverty and the low social power of young people in many societies also frame and drive sexual decision-making and risk [24]. Gender inequities, through norms and unequal gender power relations, also play an important role in determining young women's risk for intimate partner violence (IPV) and vulnerability in contracting HIV [25].

HIV incidence remains high among young African women, and few strategies have demonstrated effectiveness in reducing new HIV infections in this population. Until recently, the mainstay of HIV prevention for young men and women was behaviour change focused on delay of sexual debut, decreasing the number of sexual partners and increasing condom use through interventions delivered to individuals, couples, families, peer groups or networks, institutions or entire communities [26, 27]. Several major behavioural interventions focused on African youth had a school-based component [28–33], which provided easy access to young people, but were challenged by conservative teachers and parents uncomfortable with teaching sexual health topics to students [27]. Many youth-based interventions were focused on providing information about safe sex and teaching skills about condom negotiation but did not attempt to influence social factors or broader structural factors that shape sexual behaviour [34]; these social and structural factors are difficult to address, especially in the context of any single research study. For many women, sexual relationships involve tacit understandings where they receive consumer items, monetary gifts or other benefits and necessities from their sexual partner, and may not be able to negotiate condom use [35–40]. Encouraging results have been observed with interventions that focused on gender norms and equity in South Africa, which have shown reductions in IPV, improvements in women's well-being in the IMAGE study [41] and modest reductions in sexual risk and HSV-2 incidence in the Stepping Stones project [31]. A community-level intervention to change attitudes, social norms and behaviours related to IPV, HIV disclosure and risk reduction in women seeking HIV testing in Uganda demonstrated a reduction in some forms of IPV against women and a reduction in HIV incidence [42].

There is an urgent need for effective prevention for young African women given annual HIV incidence rates of 5–6% and higher in some settings. Oral antiretroviral pre-exposure prophylaxis (PrEP) is currently the prevention intervention for which the evidence for efficacy is greatest, if uptake and adherence are high, as described elsewhere in this issue and briefly summarized below. PrEP also is a strategy that can be independent of the partner's knowledge or “buy-in,” which could be relevant for young women who have a history of IPV or fear their partner's reaction to their efforts to reduce their risk of HIV. We focus on the barriers, opportunities and implications for implementing PrEP in young African women by reviewing behavioural and behavioural economic approaches to HIV prevention for this group. Although efforts to identify more effective and scalable behavioural and structural interventions and longer-acting, less user-dependent PrEP formulations for women must continue, it is important to evaluate the potential for PrEP as a core component of combination prevention for this key population that also includes behavioural and structural interventions, as recommended by the US President's Emergency Plan for AIDS Relief initiative for adolescent girls and young women: Determined, Resilient, Empowered, AIDS-free, Mentored and Safe (DREAMS) [43]. Defining the proportion and characteristics of women who are likely to use oral PrEP and evaluating scalable adherence strategies and delivery models will be informative in understanding delivery of long-acting PrEP formulations, multiprevention technologies and other prevention modalities to this key population.

## Discussion: evidence for PrEP as a primary HIV prevention strategy

PrEP with oral tenofovir (TDF) or TDF co-formulated with emtricitabine (TDF/FTC or Truvada^®^) demonstrated substantial HIV prevention benefits (up to 75% reduction in HIV incidence) in four trials conducted among men who have sex with men (MSM) in a multicountry trial, injection drug users in Asia, and African HIV serodiscordant couples and young men and women [44–48]. Subgroup analyses estimated efficacy to be very high (range from 80 to 92%) among those who had tenofovir in their blood samples [44–47]. PrEP efficacy among women was high (approximately 70% compared to placebo and approximately 90% when detected in blood) in the Partners PrEP Study in all women and in key subgroups, including younger women [49, 50]. In contrast, in the VOICE and FEM-PrEP trials, adherence to PrEP was very low (<30% based on a random subset with drug levels) and no efficacy was observed [51, 52]. Pharmacokinetic studies have indicated significantly lower concentrations of tenofovir in vaginal than rectal tissues [53, 54], suggesting PrEP may be less forgiving to adherence for women compared with MSM. Pharmacometric modelling is being conducted to better understand the relationship of PrEP drug exposure to efficacy by gender [55]. In addition to drug exposure, other variables, such as concomitant STIs and genital inflammation that increase HIV susceptibility [8], could modulate the efficacy of PrEP. However, the overwhelming evidence is that PrEP is biologically effective in both men and women, when taken.

The results of oral PrEP in the VOICE and FEM-PrEP trials in young African women have prompted a range of questions about next steps in HIV prevention: Did low PrEP adherence reflect low HIV risk perception; lack of motivation in the clinical trial context where they could receive placebo or an unproven product; lack of self-efficacy, external factors that limited prevention uptake, stigma, an inability to take a daily pill; a lack of motivation and interest in HIV prevention, in general, or competing daily priorities where trade-offs between current benefits and future prevention are made? Qualitative data from VOICE indicate that factors that contributed to low uptake and adherence included uncertainty and ambivalence about using antiretrovirals for prevention; concerns about drug side effects; HIV stigma associated with pill-taking; negative reactions and lack of peer, family and/or partner support and ambivalence about taking a product of uncertain efficacy in a placebo-controlled clinical trial [56, 57]. In addition, data from FEM-PrEP study participants who acquired HIV infection indicate that women underestimated their risk and rationalized their risk behaviour, that perceived risk of HIV was associated with PrEP adherence [58], and that women perceived negative consequences of reporting non-adherence [59].

Notably, the qualitative research through the VOICE and FEM-PrEP trials did not indicate that women did not want biomedical HIV prevention, but that many encountered barriers to use and desired more directive feedback. Randomized trials are very different from real-world settings, as women are motivated to participate in trials for a variety of reasons (e.g. access to quality health services and monetary reimbursement for monthly visits). Study participants are counselled monthly that they may not be receiving active product, that the active product has uncertain efficacy, and they are provided with frequent reminders and encouragement to use product, which could have discouraged participants’ willingness to accurately report their adherence and study counsellors’ ability to address their concerns and barriers to adherence. Furthermore, study participants may feel less at risk over time in the context of monthly HIV testing, potentially coupled with less imminent concern about HIV in the trial communities, given expanded access to ART and declines in HIV mortality.

A part of the rationale for learning from implementation of oral PrEP for young African women is that it has shown efficacy in multiple populations, is already available globally in branded and lower cost generic formulations, and that other PrEP formulations (e.g. topical microbicides) have not yet consistently demonstrated efficacy. Specifically, 1% tenofovir gel dosed peri-coitally (two doses within 24 hours before and after sex) demonstrated moderate efficacy (39%) against HIV infection in the initial CAPRISA 004 trial in South Africa [60]. Disappointingly, no efficacy of tenofovir gel was observed in the confirmatory FACTS 001 trial of peri-coital dosing [61] or with daily dosing in the VOICE trial [52]; in both of those trials adherence to product was low. The results of FACTS 001 and VOICE may signal that vaginal gel may not be a practical or acceptable strategy for a large enough subset of women, as a result of privacy issues for storing and carrying applicators, vaginal wetness, leakage, dislike by male partners and the need to strategize about finding the right time for gel insertion; ongoing analyses from those trials will inform next steps, if any, for that delivery approach. Other microbicide approaches, for example, slow-release vaginal rings, are being evaluated in clinical trials.

### Prioritizing oral PrEP for young women

Uptake and adherence among participants in clinical trials who are randomized to placebo or active product and counselled about *unknown* efficacy may not predict uptake and adherence when patients in real-world settings are subsequently offered open-label product and counselled about *known* efficacy and the importance of adherence to achieve protection. Evidence of this has been demonstrated by the recent PROUD study, which offered immediate or deferred open-label daily PrEP among MSM in the UK and found high effectiveness (86% HIV protection) [62]. A demonstration project of PrEP, as a bridging strategy until the HIV infected partner was on ART for six months, was conducted among high-risk HIV serodiscordant couples in Kenya and Uganda, among whom HIV transmission was almost eliminated in this time-limited use of PrEP delivered with brief adherence counselling [63]. Notably, the majority of a cohort of single, young women from Cape Town, South Africa, took open-label oral PrEP when in the ADAPT/HPTN 067 study, in which women were randomized to one of three dosing schedules (daily, intermittent weekly with a “boost” at the time of sex, or coitally-dependent dosing). Daily dosing resulted in better coverage of sex acts and adherence, and higher drug levels [64]; daily dosing may foster better habit formation and provide the most forgiveness for missed doses.

These open-label PrEP studies demonstrate the feasibility of reaching at-risk populations and achieving higher effectiveness than was observed in placebo-controlled trials, in part due to populations who recognize their risk and are motivated to access a product with known high efficacy. These demonstration projects also indicate feasibility of public health delivery with quarterly visits, brief adherence counselling, and that adherence does not have to be perfect to achieve very substantial prevention benefits. The encouraging results from these initial PrEP implementation projects call for evaluation of open-label PrEP in young African women.

A key gap is whether when counselled about the high safety, tolerability and effectiveness of PrEP in other populations, young African women who have substantial risk of HIV will effectively use PrEP until more prevention options are available. Young women may have less agency and face different circumstances and barriers than men and motivated couples in using PrEP. Specific issues for young African women include stigma about being sexually active, and limited access to youth-friendly services and reproductive health services. Thus, implementation science research is critically needed on PrEP for young women to assess their motivations for HIV prevention, PrEP delivery models including through family planning clinics and with community partners, and adherence strategies to address barriers to young women's adherence to daily pill-taking with PrEP. Innovative strategies to mitigate these barriers may include those that draw from behavioural economics and treatment adherence research with adolescents and young people, as summarized below.

### Adopting new approaches for HIV prevention: behavioural economic and economic approaches

Behavioural economics, building on insights from psychology about predictable biases and mistakes that can make it hard for individuals to make healthy choices, provides a framework for understanding why young women find it hard to consistently engage in prevention even when they are informed about the benefits. “Present-bias” leads people to focus on immediate rewards or costs at the expense of their own long-term goals and objectives [65–67]. Health prevention decisions involve immediate costs (e.g. the annoyances and cost of engaging in prevention now) and delayed and uncertain rewards (e.g. avoiding HIV infection in the future). If young women are loss averse [68], or more concerned about losses than equivalent gains, they may respond strongly to the perception that their peers or sexual partners may stigmatize certain prevention decisions. In addition, many prevention strategies reduce but cannot eliminate risk, and messaging about partial efficacy can be confusing and ambiguous. Research indicates that adversity to ambiguity leads to inaction [69] and low levels of investment in risk reduction strategies [70].

During adolescence and their early 20s, many young women experience a period of rapid social, neurodevelopmental and physical growth. Studies indicate that brain development is not complete until the early 20s, with a lag between emotional and cognitive control maturation [71]. Neurocognitive development may affect integration of risk perception and behavioural decision-making by adolescents, and an evolving ability to weigh short-term intimacy and pleasure rewards versus longer term health promotion benefits [71, 72].

Even for young women fully intending to do everything they can to prevent HIV infection, prevention decisions are made on a continual basis. If adolescents have limited attention and are juggling chaotic lives with many priorities, threats and vulnerabilities such as poverty which reduces cognitive processing, prevention behaviours may suffer [73–75]. Additionally, if young women are not fully aware of their present-bias, tend to overly discount longer term benefits and are over-confident about their ability to adhere to prevention strategies, they may not spend enough time developing prevention strategies that can be consistently implemented [67]. Finally, “optimism bias” may be an important factor, where young women may tend to overestimate the risk of a behaviour (e.g. smoking or unprotected sex) for the health of others, but underestimate their own risks [76].

One strategy for overcoming present-bias against prevention would be to increase the rewards for prevention and bring them closer to the present. Randomized trials have shown success in interventions that financially incentivize individuals to engage in prevention behaviours such as medication adherence, weight loss and smoking cessation [77, 78], although the effects often wane after incentives are discontinued [78, 79]. Incentives in conjunction with peer mentoring have been shown to be effective for achieving glucose control in diabetics [80]. Because youth are particularly sensitive to peer influence, incentives with peer mentoring or modest group level incentives may be an effective approach in young women to support habit formation of regular adherence behaviours, such as with PrEP, but have not yet been evaluated.

The evidence on cash transfers and HIV prevention suggests that they may be useful tools for addressing structural barriers that increase young women's vulnerability to HIV, especially when provided at critical times. Given findings that women who have more education generally have lower HIV risk, interventions that keep adolescents in school would increase education levels in girls and potentially reduce their engagement in transactional sex [81]. A successful example of this strategy is an evaluation of conditional cash transfers for girls’ secondary school attendance in Malawi and unconditional cash transfers, both of which showed lower HIV and HSV-2 prevalence and positive changes in the age of young women's sex partners and frequency of sex acts [82]. Other trials of conditional cash transfers to incentivize school attendance have been conducted with HIV incidence endpoints, including a trial in South Africa, which will have results in the next year [83]. Unconditional cash transfers have been associated with delayed onset of sexual activity among orphans and vulnerable children in Kenya [84], and reduced prevalence of transactional sex and age-disparate sex for adolescent girls in South Africa [85]. Cash transfers may enable women to select different partners to avoid HIV risk or they may provide bargaining power for women in relationships that are unequal. Evidence for incentives that are conditioned specifically on the outcome of staying STI and HIV negative is mixed; women have less risky sex but men receiving incentives engaged in more risky behaviour [86]. Other studies have found evidence of a reduction in bacterial STIs after one year of incentive payments [87], and a reduction in in HIV incidence through a lottery incentive scheme [88].

More work is needed to understand the mechanism driving divergent results on incentives for HIV avoidance, and for attaining sustained responses after incentives end. Although the psychology literature does not suggest that incentives “crowd out” intrinsic motivation when the baseline motivation for behaviours is low [89], more understanding is needed about whether incentives alter individuals’ intrinsic motivation to do things that are good for their health as well as the feasibility, sustainability and cost-effectiveness of incentive programs.

### Supporting adherence to oral PrEP for young women

PrEP is not suitable for all persons at risk, but may be feasible for a substantial portion of persons during “seasons” of high risk [90], which may differ by population and setting. Analysis of drug levels in the oral PrEP efficacy trials demonstrate that the majority of participants self-sorted within the first three months into users versus non-users [50]. One goal of PrEP implementation projects is to understand the motivations of persons at risk of HIV to use PrEP and to help support formation and continuation of early adherence behaviours. For young women who have had limited healthcare utilization and minimal experience with long-term medications, PrEP adherence support should focus on practical issues, including counselling about possible early and transient gastrointestinal symptoms, dosing, product storage, “pocket doses” when away from home, and incorporation of a regular behaviour like pill-taking with daily routines. Peer support and modest, short-term incentives might provide additional support for adherence to PrEP, but need to be evaluated. Given the importance of peers and social norms among young women, incentives for PrEP adherence may be more powerful if delivered as part of peer adherence mentoring and support.

Young women may have greater difficulty with adherence than older women and other populations, in part due to cognitive development [91]. Psychological and social barriers are significant predictors of non-adherence among studies of adolescents with a range of chronic illnesses with medication adherence rates ranging from 20 to 60% [92–95]. Poor adherence to ART has been linked to depression, gender-based violence and lack of social support, all of which are common in young people in sub-Saharan Africa [96–99]. Lower rates of adherence to preventive health interventions have been observed among youth, as demonstrated by lower adherence to oral contraceptives among adolescent and young adult women at high risk of unintended pregnancy [100–102].

With respect to adherence support for young African women, recent meta-analyses highlight that cognitive-behavioural problem-solving [103, 104] and peer support [105] consistently improve medication adherence among adults in the United States and developing countries. Cognitive-behavioural approaches have been used to treat youth depression [106] and trauma [107], improve adult and youth ART adherence [104, 105, 108] and decrease alcohol use in HIV-infected sub-Saharan African adults [109]. Cognitive-behavioural therapy (CBT) is a brief, cost-effective intervention strategy that can be implemented by a wide range of providers, including non-specialists and those with little counselling experience, in both clinical and community-based settings in low resource settings [110–112]. Most research on adherence counselling based on CBT is from the treatment field, and evaluation is needed on CBT to support prevention behaviours. The Life Steps programme is an example of CBT, incorporating informational, problem-solving and cognitive-behavioural strategies to address barriers to ART adherence, which was successfully used to support PrEP adherence among HIV serodiscordant couples in the Partners PrEP Study [113], and could be adapted to counsel young women about PrEP adherence challenges.

The revolution in mobile phone technology creates new opportunities to engage youth and build new social relationships and networks with adolescents. These opportunities can be leveraged for health. SMS reminders provide cognitive reminders for adherence and can strengthen communications between patients and providers [114]. A recent meta-analysis found that text messaging can be an effective support for ART adherence, particularly with messaging provided on a less than daily basis, content and timing that is individually tailored and platforms designed to evoke a reply from the recipient [115]. SMS have also increased oral contraceptive continuation rates [116] and could be incorporated to provide cognitive reminders for PrEP among women as they are establishing adherence habits.

## Conclusions: priorities for implementation of oral PrEP for young African women

With strong evidence for the efficacy and effectiveness of oral PrEP across multiple studies, it is essential to understand whether young women will be interested in using PrEP and able to sustain adherence in the context of clear positive messaging and integrated with delivery of other services that meet young women's needs (e.g. family planning, emergency contraception, post-exposure prophylaxis, IPV and skills training). PrEP demonstration projects for young African women provide an opportunity for evaluation of delivery models, innovative communications, adherence and behavioural economics interventions to support uptake of effective, novel HIV prevention interventions. These opportunities include evaluating strategies to make risk salient to young women, understand and maximize women's motivations for prevention, and use of social support and social media to support PrEP uptake adherence, and delivery models, as summarized in Table 1.

**Table 1:** Considerations for future research with oral PrEP for young African women

Topic	Strategy
1. Understand the end-users for HIV prevention during product development and delivery	• Conduct formative research to understand young African women's needs and preferences about products and delivery strategies for HIV prevention. Methods that could be useful include ethnographic research as part of behaviour-centred and user-centred design, and mental models approaches • Evaluate young women's decision-making about HIV prevention • Assess end-users’ preferences in designing new products
2. Test communication messages and demand creation strategies	• Rigorously test different communication messages to determine which are most salient and young women respond to most strongly, including: ○ Emotion and desire for status are important drivers of decisions ○ Deemphasize messages about risk, which is ambiguous, dynamic and can stigmatize sexuality, potentially increasing acceptability for using HIV prevention methods ○ Frame prevention messaging positively in terms of benefits in intimacy, self-esteem and desire to achieve one's aspirations ○ Identify ‘positive deviants’ and disseminate compelling narratives from women who have successfully utilized PrEP • Identify effective strategies for demand creation, including ones from implementation of contraceptive services. • Use technology to achieve “wide reach” in disseminating messages and promoting HIV prevention, with interactive platforms, such as social media
3. Develop innovative interventions to motivate HIV prevention behaviours, including PrEP	• Use formative research informed by behaviour-centred design to identify evolutionary motivators and pilot interventions addressing these motivators • Identify ‘levers’ for initiation of PrEP and continued use, pilot and evaluate interventions to increase these behaviours • Identify ways to make pill-taking behaviours automatic and part of young women's routine practices • Evaluate whether a validated empiric risk score increases the uptake of oral PrEP among young women with a significant risk of HIV acquisition • Address medication maintenance factors such as transport and financial barriers for refills, and stigma
4. Evaluate PrEP effectiveness among young African women	• Conduct a demonstration project of open-label oral PrEP among young African women with HIV incidence as the primary outcome, either using a counterfactual with HIV rates in recent trials among women of similar risk or in an immediate-deferred design (such as the PROUD study among MSM in the UK)
5. Test delivery models of PrEP, including integration with family planning and other services	• Evaluate whether uptake of HIV prevention is higher if offered with contraceptive counselling and services (e.g. cervical cancer and STI screening, gender-based violence counselling) as a separate service, and through clinics or community programmes • Test different HIV prevention delivery models, including offering PrEP when women seek contraception (e.g. injectables, implants) or emergency contraception and post-exposure prophylaxis. • As additional PrEP formulations become available (e.g. vaginal rings, injectables), evaluate the family planning delivery model of providing women with choice of methods • Assess cost-effectiveness of delivery of PrEP in demonstration projects
6. Assess behavioural economic approaches to HIV prevention	• Assess whether group or individual incentives are effective for young women's initiation of and/or adherence to PrEP. • Evaluate HIV prevention technologies with measurement of the barriers between inaction and action (e.g. present bias, limited attention, cognitive capacity, bounded rationality including partial information, rumour, inaccuracies about sex and HIV) • Deliver HIV prevention with a focus on convenience, bundling with other services, simple and clear messages about benefits/costs and reminders

The effectiveness of positive communication messages designed using insights from ethnographic research, mental models [117] and other behavioural models should be evaluated to determine whether effectively framed messages about how PrEP helps young women meet their aspirations, and achieve greater intimacy, self-esteem, love and confidence resonate with young women more than messages that are framed with disease prevention as the only benefit [118]. Behavioural economics could be useful in smarter design of communication campaigns about PrEP, by incorporating and addressing biases in decision-making. Furthermore, insights from behavioural economics could be useful in focusing attention on beliefs and emotions in making decisions [119]. Evaluation is needed of social marketing of HIV prevention including PrEP, using the internet for promotion of effective HIV interventions, as well as digital games, phone apps and narratives to provide more salient and effective methods for engaging youth and correcting incorrect beliefs and perceptions about HIV risk [120]. Demonstration projects should assess the use of risk scores that stratify women according to their risk of HIV; one example is the risk score developed from analyses of HIV seroconverters in the VOICE trial [121].

PrEP delivery models need to be piloted for young women who have limited access to reproductive and sexual health services in many parts of Africa, in part due to provider attitudes towards sexually active unmarried women. Family planning clinics could be an efficient, existing delivery site for PrEP, by reaching women with overlapping risks for pregnancy and HIV and creating synergies in integrated rather than parallel delivery programmes. Women who are seeking services at youth-friendly clinics or family planning services are potential early adopters of PrEP because they are already engaging with the healthcare system and are already motivated by prevention (i.e. to receive reproductive health services and to reduce their risk of pregnancy). In addition, in some African countries legislation is needed to permit adolescents to access sexual and reproductive health services to remove this structural barrier to therapeutic and prevention services in general, including PrEP.

PrEP implementation studies for young African women need to evaluate sociocultural contextual issues, caregiver support, peer norms and relations, and cognitive and environmental factors that influence uptake and adherence to biomedical HIV preventions. Ethnographic research is needed to better understand sexual partnerships, social influencers, and household factors (e.g. composition, stability, privacy and communication about sexuality) that shape young women's agency, priorities and ability to seek and adopt HIV prevention, including PrEP. Given the importance of context, sexual norms and expectations, and structural factors on increasing risk and vulnerability for young African women, PrEP may have greater uptake and adherence if it is provided in combination with counselling, peer support and referrals for women who have experienced sexual violence, have alcohol dependence or other needs^122^. PrEP demonstration projects are being launched for sex worker populations in Africa to understand delivery, uptake and adherence to PrEP, with the assumption that sex workers may have different motivations, barriers and delivery strategies for PrEP than young African women in the general population.

PrEP demand should be quantified in terms of uptake and continuation rates among women of different ages and partnership characteristics in different settings as part of market segmentation research, which could also help forecast programme costs and supply chain needs. Scalable adherence strategies, such as brief counselling based on CBT, two-way interactive SMS messages as cognitive reminders, peer support groups and short-term incentives to support adherence behaviours need to be evaluated among young African women in open-label PrEP demonstration projects. Demonstration projects will be most policy informative if they are sufficiently large and designed to assess HIV incidence, either through a counterfactual or with immediate versus delayed PrEP use.

Oral PrEP is a first-generation product, which has many similarities to the oral contraceptive pill which also has adherence challenges but made a significant impact on pregnancy prevention and paved the way for a multitude of contraceptive methods [123]. Adherence to daily pill-taking may limit the market segment of women who are able to benefit from PrEP, but this needs to be assessed and quantified. However, important lessons can be learned from demonstration projects of PrEP, as the demonstration projects among MSM in Europe and couples in Africa have demonstrated, which showed that men and couples at high risk for HIV were able to take PrEP with sufficient adherence to achieve a very substantial HIV prevention benefit. Although there is great enthusiasm for the potential of longer-acting products to address adherence challenges of daily pill-taking with PrEP, these may also be associated with issues about uptake, if use of any formulation of PrEP requires risk perception and engagement with HIV prevention to generate sufficient interest. Lessons learned through implementation of oral PrEP, a first-generation biomedical HIV prevention product, will inform development of new PrEP formulations, including intravaginal rings and injectable PrEP, with the goal to eventually offer an expanding choice of prevention options in integrated, combination programmes for young African women.

## References

[1] UNAIDS (2014). The Gap Report.

[2] Ng'ang'a A, Waruiru W, Ngare C, Ssempijja V, Gachuki T, Njoroge I (2014). The status of HIV testing and counseling in Kenya: results from a nationally representative population-based survey. J Acquir Immune Defic Syndr.

[3] Lahuerta M, Wu Y, Hoffman S, Elul B, Kulkarni SG, Remien RH (2014). Advanced HIV disease at entry into HIV care and initiation of antiretroviral therapy during 2006–2011: findings from four sub-saharan African countries. Clin Infect Dis.

[4] Wasserheit JN (1992). Epidemiological synergy. Interrelationships between human immunodeficiency virus infection and other sexually transmitted diseases. Sex Transm Dis.

[5] Rottingen JA, Cameron DW, Garnett GP (2001). A systematic review of the epidemiologic interactions between classic sexually transmitted diseases and HIV: how much really is known?. Sex Transm Dis.

[6] Hayes R, Watson-Jones D, Celum C, van de Wijgert J, Wasserheit J (2010). Treatment of sexually transmitted infections for HIV prevention: end of the road or new beginning?. AIDS.

[7] Celum CL (2010). Sexually transmitted infections and HIV: epidemiology and interventions. Top HIV Med.

[8] Naranbhai V, Abdool Karim SS, Altfeld M, Samsunder N, Durgiah R, Sibeko S (2012). Innate immune activation enhances HIV acquisition in women, diminishing the effectiveness of tenofovir microbicide gel. J Infect Dis.

[9] Gray RH, Kiwanuka N, Quinn TC, Sewankambo NK, Serwadda D, Mangen FW (2000). Male circumcision and HIV acquisition and transmission: cohort studies in Rakai, Uganda. Rakai Project Team. AIDS.

[10] Baeten JM, Donnell D, Kapiga SH, Ronald A, John-Stewart G, Inambao M (2010). Male circumcision and risk of male-to-female HIV-1 transmission: a multinational prospective study in African HIV-1-serodiscordant couples. AIDS.

[11] Gray RH, Wawer MJ, Brookmeyer R, Sewankambo NK, Serwadda D, Wabwire-Mangen F (2001). Probability of HIV-1 transmission per coital act in monogamous, heterosexual, HIV-1-discordant couples in Rakai, Uganda. Lancet.

[12] Hughes JP, Baeten JM, Lingappa JR, Magaret AS, Wald A, de Bruyn G (2012). Determinants of per-coital-act HIV-1 infectivity among African HIV-1-serodiscordant couples. J Infect Dis.

[13] Gray RH, Li X, Kigozi G, Serwadda D, Brahmbhatt H, Wabwire-Mangen F (2005). Increased risk of incident HIV during pregnancy in Rakai, Uganda: a prospective study. Lancet.

[14] Polis CB, Curtis KM (2013). Use of hormonal contraceptives and HIV acquisition in women: a systematic review of the epidemiological evidence. Lancet Infect Dis.

[15] Pettifor AE, van der Straten A, Dunbar MS, Shiboski SC, Padian NS (2004). Early age of first sex: a risk factor for HIV infection among women in Zimbabwe. AIDS.

[16] Pettifor A, O'Brien K, Macphail C, Miller WC, Rees H (2009). Early coital debut and associated HIV risk factors among young women and men in South Africa. Int Perspect Sex Reprod Health.

[17] Hallett TB, Gregson S, Lewis JJ, Lopman BA, Garnett GP (2007). Behaviour change in generalised HIV epidemics: impact of reducing cross-generational sex and delaying age at sexual debut. Sex Transm Infect.

[18] Halperin DT, Epstein H (2004). Concurrent sexual partnerships help to explain Africa's high HIV prevalence: implications for prevention. Lancet.

[19] Mah TL, Halperin DT (2010). Concurrent sexual partnerships and the HIV epidemics in Africa: evidence to move forward. AIDS Behav.

[20] Reddy SP JS, Sewpaul R, Koopman F, Funani NI, Sifunda S, Josie J (2010). Umthente Uhlaba Usamila – The South African Youth Risk Behaviour Survey 2008.

[21] Jewkes RK, Dunkle K, Nduna M, Shai N (2010). Intimate partner violence, relationship power inequity, and incidence of HIV infection in young women in South Africa: a cohort study. Lancet.

[22] Jewkes RK, Levin JB, Penn-Kekana LA (2003). Gender inequalities, intimate partner violence and HIV preventive practices: findings of a South African cross-sectional study. Soc Sci Med.

[23] Fisher JC, Bang H, Kapiga SH (2007). The association between HIV infection and alcohol use: a systematic review and meta-analysis of African studies. Sex Transm Dis.

[24] Gupta GR, Parkhurst JO, Ogden JA, Aggleton P, Mahal A (2008). Structural approaches to HIV prevention. Lancet.

[25] Garcia-Moreno C, Jansen HA, Ellsberg M, Heise L, Watts CH (2006). Health WHOM-cSoWs, Domestic violence against women study T. Prevalence of intimate partner violence: findings from the WHO multi-country study on women's health and domestic violence. Lancet.

[26] Coates TJ, Richter L, Caceres C (2008). Behavioural strategies to reduce HIV transmission: how to make them work better. Lancet.

[27] Mavedzenge S, Luecke E, Ross D (2014). Effective approaches for programming to reduce adolescent vulnerability to HIV infection, risk, and HIV-related morbidity and mortality. J Acquir Immune Defic Syndr.

[28] Cowan FM, Pascoe SJ, Langhaug LF, Mavhu W, Chidiya S, Jaffar S (2010). The Regai Dzive Shiri project: results of a randomized trial of an HIV prevention intervention for youth. AIDS.

[29] Doyle AM, Ross DA, Maganja K, Baisley K, Masesa C, Andreasen A (2010). Long-term biological and behavioural impact of an adolescent sexual health intervention in Tanzania: follow-up survey of the community-based MEMA kwa Vijana Trial. PLoS Med.

[30] Pettifor AE, Kleinschmidt I, Levin J, Rees HV, MacPhail C, Madikizela-Hlongwa L (2005). A community-based study to examine the effect of a youth HIV prevention intervention on young people aged 15–24 in South Africa: results of the baseline survey. Trop Med Int Health.

[31] Jewkes R, Nduna M, Levin J, Jama N, Dunkle K, Puren A (2008). Impact of stepping stones on incidence of HIV and HSV-2 and sexual behaviour in rural South Africa: cluster randomised controlled trial. BMJ.

[32] NIMH Collaborative HIV/STD Prevention Trial Group (2010). Results of the NIMH collaborative HIV/sexually transmitted disease prevention trial of a community popular opinion leader intervention. J Acquir Immune Defic Syndr.

[33] Wingood GM, DiClemente RJ (2000). Application of the theory of gender and power to examine HIV-related exposures, risk factors, and effective interventions for women. Health Educ Behav.

[34] Wight D, Plummer M, Ross D (2012). The need to promote behaviour change at the cultural level: one factor explaining the limited impact of the MEMA kwa Vijana adolescent sexual health intervention in rural Tanzania. A process evaluation. BMC Public Health.

[35] Underwood C, Skinner J, Osman N, Schwandt H (2011). Structural determinants of adolescent girls’ vulnerability to HIV: views from community members in Botswana, Malawi, and Mozambique. Soc Sci Med.

[36] Hawkins K, Price N, Mussa F (2009). Milking the cow: young women's construction of identity and risk in age-disparate transactional sexual relationships in Maputo, Mozambique. Glob Public Health.

[37] Silberschmidt M, Rasch V (2001). Adolescent girls, illegal abortions and “sugar-daddies” in Dar es Salaam: vulnerable victims and active social agents. Soc Sci Med.

[38] Wamoyi J, Wight D, Plummer M, Mshana GH, Ross D (2010). Transactional sex amongst young people in rural northern Tanzania: an ethnography of young women's motivations and negotiation. Reprod Health.

[39] Hunter M (2002). The materiality of everyday sex: thinking beyond ‘prostitution’. Afr Stud.

[40] Wamoyi J, Fenwick A, Urassa M, Zaba B, Stones W (2011). “Women's bodies are shops”: beliefs about transactional sex and implications for understanding gender power and HIV prevention in Tanzania. Arch Sex Behav.

[41] Pronyk PM, Kim JC, Abramsky T, Phetla G, Hargreaves JR, Morison LA (2008). A combined microfinance and training intervention can reduce HIV risk behaviour in young female participants. AIDS.

[42] Wagman JA, Gray RH, Campbell JC, Thoma M, Ndyanabo A, Ssekasanvu J (2015). Effectiveness of an integrated intimate partner violence and HIV prevention intervention in Rakai, Uganda: analysis of an intervention in an existing cluster randomised cohort. Lancet Glob Health.

[43] U.S. President's Emergency Plan for AIDS Relief (PEPFAR) The U.S. President's Emergency Plan for AIDS Relief, The Bill & Melinda Gates Foundation, and The Nike Foundation Partner on $210 million initiative to reduce new HIV infections in adolescent girls and young women.

[44] Grant RM, Lama JR, Anderson PL, McMahan V, Liu AY, Vargas L (2010). Preexposure chemoprophylaxis for HIV prevention in men who have sex with men. N Engl J Med.

[45] Baeten JM, Donnell D, Ndase P, Mugo NR, Campbell JD, Wangisi J (2012). Antiretroviral prophylaxis for HIV prevention in heterosexual men and women. N Engl J Med.

[46] Choopanya K, Martin M, Suntharasamai P, Sangkum U, Mock PA, Leethochawalit M (2013). Antiretroviral prophylaxis for HIV infection in injecting drug users in Bangkok, Thailand (the Bangkok Tenofovir Study): a randomised, double-blind, placebo-controlled phase 3 trial. Lancet.

[47] Thigpen MC, Kebaabetswe PM, Paxton LA, Smith DK, Rose CE, Segolodi TM (2012). Antiretroviral preexposure prophylaxis for heterosexual HIV transmission in Botswana. N Engl J Med.

[48] Baeten JM, Haberer JE, Liu AY, Sista N (2013). Preexposure prophylaxis for HIV prevention: where have we been and where are we going?. J Acquir Immune Defic Syndr.

[49] Murnane PM, Celum C, Mugo N, Campbell JD, Donnell D, Bukusi E (2013). Efficacy of preexposure prophylaxis for HIV-1 prevention among high-risk heterosexuals: subgroup analyses from a randomized trial. AIDS.

[50] Donnell D, Baeten JM, Bumpus NN, Brantley J, Bangsberg DR, Haberer JE (2014). HIV protective efficacy and correlates of tenofovir blood concentrations in a clinical trial of PrEP for HIV prevention. J Acquir Immune Defic Syndr.

[51] Van Damme L, Corneli A, Ahmed K, Agot K, Lombaard J, Kapiga S (2012). Preexposure prophylaxis for HIV infection among African women. N Engl J Med.

[52] Marrazzo JM, Ramjee G, Richardson BA, Gomez K, Mgodi N, Nair G (2015). Tenofovir-based preexposure prophylaxis for HIV infection among African women. N Engl J Med.

[53] Patterson KB, Prince HA, Kraft E, Jenkins AJ, Shaheen NJ, Rooney JF (2011). Penetration of tenofovir and emtricitabine in mucosal tissues: implications for prevention of HIV-1 transmission. Sci Transl Med.

[54] Louissaint NA, Cao YJ, Skipper PL, Liberman RG, Tannenbaum SR, Nimmagadda S (2013). Single dose pharmacokinetics of oral tenofovir in plasma,peripheral blood mononuclear cells, colonic tissue, and vaginal tissue. AIDS Res Hum Retroviruses.

[55] Hendrix CW (2013). Exploring concentration response in HIV pre-exposure prophylaxis to optimize clinical care and trial design. Cell.

[56] van der Straten A, Stadler J, Montgomery E, Hartmann M, Magazi B, Mathebula F (2014). Women's experiences with oral and vaginal pre-exposure prophylaxis: the VOICE-C qualitative study in Johannesburg, South Africa. PLoS One.

[57] van der Straten A, Stadler J, Luecke E, Laborde N, Hartmann M, Montgomery ET (2014). Perspectives on use of oral and vaginal antiretrovirals for HIV prevention: the VOICE-C qualitative study in Johannesburg, South Africa. J Int AIDS Soc.

[58] Corneli A, Wang M, Agot K, Ahmed K, Lombaard J, Van Damme L (2014). Perception of HIV risk and adherence to a daily, investigational pill for HIV prevention in FEM-PrEP. J Acquir Immune Defic Syndr.

[59] Corneli AL, McKenna K, Perry B, Ahmed K, Agot K, Malamatsho F (2015). The science of being a study participant: FEM-PrEP participants’ explanations for overreporting adherence to the study pills and for the whereabouts of unused pills. J Acquir Immune Defic Syndr.

[60] Abdool Karim Q, Abdool Karim SS, Frohlich JA, Grobler AC, Baxter C, Mansoor LE (2010). Effectiveness and safety of tenofovir gel, an antiretroviral microbicide, for the prevention of HIV infection in women. Science.

[61] Rees H, Delaney-Moretlwe S, Baron D, Lombard C, Gray G, Myer L Facts 001 phase iii trial of pericoital tenofovir 1% gel for HIV prevention in women.

[62] McCormack S, Dunn D Pragmatic open-label randomised trial of preexposure prophylaxis: the PROUD study.

[63] Baeten J, Heffron R, Kidoguchi L, Mugo N, Bukusi E, Katabira E Near elimination of HIV transmission in a demonstration project of PrEP and ART.

[64] Bekker L-G, Grant R, Hughes J, Roux S, Amico R, Hendrix C HPTN 067/ADAPT Cape Town: a comparison of daily and nondaily PrEP dosing in African women.

[65] Laibson D (1997). Golden eggs and hyperbolic discounting. Q J Econ.

[66] O'Donoghue T, Rabin M (1999). Doing it now or later. Am Econ Rev.

[67] Linnemayr S (2015). HIV prevention through the lens of behavioral economics. J Acquir Immune Defic Syndr.

[68] Kahneman D, Tversky A (1979). Prospect theory – analysis of decision under risk. Econometrica.

[69] Fox CR, Tversky A (1995). Ambiguity aversion and comparative ignorance. Q J Econ.

[70] Bryan G (2013). Ambiguity Aversion Decreases Demand for Partial Insurance: Evidence from African Farmers. London School of Economics and Political Science.

[71] Johnson SB, Blum RW, Giedd JN (2009). Adolescent maturity and the brain: the promise and pitfalls of neuroscience research in adolescent health policy. J Adolesc Health.

[72] Giedd JN, Blumenthal J, Jeffries NO, Castellanos FX, Liu H, Zijdenbos A (1999). Brain development during childhood and adolescence: a longitudinal MRI study. Nat Neurosci.

[73] Baicker K, Mullainathan S, Schwartzstein J (2012). Behavioral Hazard in Health Insurance. National Bureau of Economic Research.

[74] Mani A, Mullainathan S, Shafir E, Zhao J (2013). Poverty impedes cognitive function. Science.

[75] Shah AK, Mullainathan S, Shafir E (2012). Some consequences of having too little. Science.

[76] Weinstein ND (1989). Optimistic biases about personal risks. Science.

[77] Volpp KG, Troxel AB, Pauly MV, Glick HA, Puig A, Asch DA (2009). A randomized, controlled trial of financial incentives for smoking cessation. N Engl J Med.

[78] Volpp KG, John LK, Troxel AB, Norton L, Fassbender J, Loewenstein G (2008). Financial incentive-based approaches for weight loss: a randomized trial. JAMA.

[79] Royer H, Stehr M, Sydnor J (2013). Incentives, commitments and habit formation in exercise: evidence from a field experiment with workers at a Fortune – 500 company. National Bureau of Economic Research.

[80] Long JA, Jahnle EC, Richardson DM, Loewenstein G, Volpp KG (2012). Peer mentoring and financial incentives to improve glucose control in African American veterans: a randomized trial. Ann Intern Med.

[81] Heise L, Lutz B, Ranganathan M, Watts C (2013). Cash transfers for HIV prevention: considering their potential. J Int AIDS Soc.

[82] Baird SJ, Garfein RS, McIntosh CT, Ozler B (2012). Effect of a cash transfer programme for schooling on prevalence of HIV and herpes simplex type 2 in Malawi: a cluster randomised trial. Lancet.

[83] Pettifor A, MacPhail C, Nguyen N, Rosenberg M (2012). Can money prevent the spread of HIV? A review of cash payments for HIV prevention. AIDS Behav.

[84] Handa S, Halpern CT, Pettifor A, Thirumurthy H (2014). The Government of Kenya's cash transfer program reduces the risk of sexual debut among young people age 15–25. PLoS One.

[85] Cluver L, Boyes M, Orkin M, Pantelic M, Molwena T, Sherr L (2013). Child-focused state cash transfers and adolescent risk of HIV infection in South Africa: a propensity-score-matched case-control study. Lancet Glob Health.

[86] Kohler HP, Thornton R (2012). Conditional cash transfers and HIV/AIDS prevention: unconditionally promising?. World Bank Econ Rev.

[87] de Walque D, Dow WH, Nathan R, Abdul R, Abilahi F, Gong E (2012). Incentivising safe sex: a randomised trial of conditional cash transfers for HIV and sexually transmitted infection prevention in rural Tanzania. BMJ Open.

[88] De Walque D, Bjorkman-Nyqvist M, Corno L, Svensson J Evaluating the impact of short term financial incentives on HIV and STI incidence among youth in Lesotho: a randomized trial.

[89] Promberger M, Marteau TM (2013). When do financial incentives reduce intrinsic motivation? comparing behaviors studied in psychological and economic literatures. Health Psychol.

[90] Mugo N No silver bullet: HIV & AIDS challenges and solutions.

[91] Roalf DR, Gur RE, Ruparel K, Calkins ME, Satterthwaite TD, Bilker WB (2014). Within-individual variability in neurocognitive performance: age- and sex-related differences in children and youths from ages 8 to 21. Neuropsychology.

[92] Rapoff M (1999). Adherence to pediatric medical regimens.

[93] Tebbi CK (1993). Treatment compliance in childhood and adolescence. Cancer.

[94] Pidgeon V (1989). Compliance with chronic illness regimens: school-aged children and adolescents. J Pediatr Nurs.

[95] Litt IF, Cuskey WR (1980). Compliance with medical regimens during adolescence. Pediatr Clin North Am.

[96] Nakimuli-Mpungu E, Bass JK, Alexandre P, Mills EJ, Musisi S, Ram M (2012). Depression, alcohol use and adherence to antiretroviral therapy in sub-Saharan Africa: a systematic review. AIDS Behav.

[97] Adewuya AO, Afolabi MO, Ola BA, Ogundele OA, Ajibare AO, Oladipo BF (2010). The effect of psychological distress on medication adherence in persons with HIV infection in Nigeria. Psychosomatics.

[98] Etienne M, Hossain M, Redfield R, Stafford K, Amoroso A (2010). Indicators of adherence to antiretroviral therapy treatment among HIV/AIDS patients in 5 African countries. J Int Assoc Physicians AIDS Care (Chic).

[99] Murray LK, Semrau K, McCurley E, Thea DM, Scott N, Mwiya M (2009). Barriers to acceptance and adherence of antiretroviral therapy in urban Zambian women: a qualitative study. AIDS Care.

[100] Berenson AB, Rahman M (2012). A randomized controlled study of two educational interventions on adherence with oral contraceptives and condoms. Contraception.

[101] Rosenberg MJ, Waugh MS (1998). Oral contraceptive discontinuation: a prospective evaluation of frequency and reasons. Am J Obstet Gynecol.

[102] Molloy GJ, Graham H, McGuinness H (2012). Adherence to the oral contraceptive pill: a cross-sectional survey of modifiable behavioural determinants. BMC Public Health.

[103] Safren SA, Otto MW, Worth JL, Salomon E, Johnson W, Mayer K (2001). Two strategies to increase adherence to HIV antiretroviral medication: life-steps and medication monitoring. Behavior Res Ther.

[104] Safren SA, O'Cleirigh C, Tan JY, Raminani SR, Reilly LC, Otto MW (2009). A randomized controlled trial of cognitive behavioral therapy for adherence and depression (CBT-AD) in HIV-infected individuals. Health Psychol.

[105] Simoni JM, Huh D, Frick PA, Pearson CR, Andrasik MP, Dunbar PJ (2009). Peer support and pager messaging to promote antiretroviral modifying therapy in Seattle: a randomized controlled trial. J Acquir Immune Defic Syndr.

[106] Wethington HR, Hahn RA, Fuqua-Whitley DS, Sipe TA, Crosby AE, Johnson RL (2008). The effectiveness of interventions to reduce psychological harm from traumatic events among children and adolescents: a systematic review. Am J Prev Med.

[107] Cohen JA, Mannarino AP, Murray LK (2011). Trauma-focused CBT for youth who experience ongoing traumas. Child Abuse Negl.

[108] Kennard B, Brown L, Hawkins L, Risi A, Radcliffe J, Emslie G (2014). Development and implementation of health and wellness CBT for individuals with depression and HIV. Cogn Behav Pract.

[109] Papas RK, Sidle JE, Gakinya BN, Baliddawa JB, Martino S, Mwaniki MM (2011). Treatment outcomes of a stage 1 cognitive-behavioral trial to reduce alcohol use among human immunodeficiency virus-infected out-patients in western Kenya. Addiction.

[110] Bolton P, Bass J, Betancourt T, Speelman L, Onyango G, Clougherty KF (2007). Interventions for depression symptoms among adolescent survivors of war and displacement in northern Uganda: a randomized controlled trial. JAMA.

[111] Bolton P, Bass J, Neugebauer R, Verdeli H, Clougherty KF, Wickramaratne P (2003). Group interpersonal psychotherapy for depression in rural Uganda: a randomized controlled trial. JAMA.

[112] Rahman A, Malik A, Sikander S, Roberts C, Creed F (2008). Cognitive behaviour therapy-based intervention by community health workers for mothers with depression and their infants in rural Pakistan: a cluster-randomised controlled trial. Lancet.

[113] Psaros C, Haberer JE, Katabira E, Ronald A, Tumwesigye E, Campbell JD (2014). An intervention to support HIV preexposure prophylaxis adherence in HIV-serodiscordant couples in Uganda. J Acquir Immune Defic Syndr.

[114] Osterberg L, Blaschke T (2005). Adherence to medication. N Engl J Med.

[115] Finitsis DJ, Pellowski JA, Johnson BT (2014). Text message intervention designs to promote adherence to antiretroviral therapy (ART): a meta-analysis of randomized controlled trials. PLoS One.

[116] Castano PM, Bynum JY, Andres R, Lara M, Westhoff C (2012). Effect of daily text messages on oral contraceptive continuation: a randomized controlled trial. Obstet Gynecol.

[117] Morgan M, Fischhoff B, Bostom A, Atman C (2001). Risk communication: the mental models approach.

[118] O'Keefe DJ, Jensen JD (2007). The relative persuasiveness of gain-framed and loss-framed messages for encouraging disease prevention behaviors: a meta-analytic review. J Health Commun.

[119] Ariely D, Loewenstein G (2006). The heat of the moment: the effect of sexual arousal on sexual decision making. J Behav Decis Making.

[120] Datta S, Burns J, Maughan-Brown B, Darling M, Eyal K Risking it all for love? Resetting beliefs about HIV risk among low-income South African teens. J Econ Behav Organ.

[121] Balkus JE, Zhang JY, Nair G, Palanee T, Ramjee G, Nakabiito C (2014). Development of a risk scoring tool to predict HIV-1 acquisition in African women. AIDS Res Hum Retroviruses.

[122] Sikkema KJ, Neufeld SA, Hansen NB, Mohlahlane R, Van Rensburg MJ, Watt MH (2010). Integrating HIV prevention into services for abused women in South Africa. AIDS Behav.

[123] Myers JE, Sepkowitz KA (2013). A pill for HIV prevention: deja vu all over again?. Clin Infect Dis.

# Sub article: Translating PrEP effectiveness into public health impact: key considerations for decision-makers on cost-effectiveness, price, regulatory issues, distributive justice and advocacy for access

**DOI:** 10.7448/IAS.18.4.19973

**Published:** 2015-07-20

**Authors:** Catherine Hankins, Ruth Macklin, Mitchell Warren

**Affiliations:** 1 Department of Global Health, Academic Medical Center, Amsterdam Institute for Global Health and Development, University of Amsterdam, Amsterdam, The Netherlands; 2 Department of Infectious Disease Epidemiology, Faculty of Epidemiology and Population Health, London School of Hygiene and Tropical Medicine, London, England; 3 Epidemiology & Population Health, Albert Einstein College of Medicine New York, NY, USA; 4 AVAC New York, NY, USA

**Keywords:** HIV prevention, pre-exposure prophylaxis, ethics, cost-effectiveness, regulatory, antiretroviral, tenofovir/emtricitabine, resource allocation

## Abstract

**Introduction:**

The extraordinary feat of proving the effectiveness of oral pre-exposure prophylaxis (PrEP) in clinical trials in different populations in a variety of settings may prove to have been easier than ensuring it is used well. Decision-makers must make difficult choices to realize the promise of antiretroviral prophylaxis for their countries. This paper outlines key economic, regulatory and distributive justice issues that must be addressed for effective and acceptable PrEP implementation.

**Discussion:**

In considering the role that PrEP can play in combination prevention programmes, decision-makers must determine who can benefit most from PrEP, how PrEP can be provided safely and efficiently, and what kind of health system support will ensure successful implementation. To do this, they need contextualized information on disease burden by population, analyses of how PrEP services might best be delivered, and projections of the human resource and infrastructure requirements for each potential delivery model. There are cost considerations, varying cost-effectiveness results and regulatory challenges. The principles of ethics can inform thorny discussions about who should be prioritized for oral PrEP and how best to introduce it fairly. We describe the cost-effectiveness of PrEP in different populations at higher risk of HIV exposure, its price in low- and middle-income countries, and the current regulatory situation. We explore the principles of ethics that can inform resource allocation decision-making about PrEP anchored in distributive justice, at a time when universal access to antiretroviral treatment remains to be assured. We then highlight the role of advocacy in moving the PrEP agenda forward.

**Conclusions:**

The time is ripe now for decisions about whether, how and for whom PrEP should be introduced into a country's HIV response. It has the potential to contribute significantly to high impact HIV prevention if it is tailored to those who can most benefit from it and if current regulatory and pricing barriers can be overcome. Advocacy at all levels can help inform decision-making and push the access agenda to avert HIV infections among those at highest risk of HIV exposure. The benefits will accrue beyond the individual level to slow HIV transmission at the population level.

## Introduction

In clinical trials conducted in Africa, Asia, Europe and North America over the past decade, pre-exposure prophylaxis (PrEP), using oral formulations of the antiretroviral drugs tenofovir or tenofovir/emtricitabine (TDF/FTC), has been shown to significantly reduce the risk of HIV acquisition among men who have sex with men (MSM) [1–3], heterosexual men and women [4, 5], and people who inject drugs (PWID) [6]. Trials involving monthly insertion of a vaginal ring containing the antiretroviral dapivirine will report results in 2016 [7, 8]. Following good safety signals [9], long-acting PrEP is currently being investigated in trials of injectable rilpivirine [10] and cabotegravir [11].

With vaginal rings and injectables potentially coming on the heels of proven oral PrEP, it is important to consider how best to introduce these new options into current HIV combination prevention strategies [12, 13] to achieve reductions in HIV risk at individual and community levels. Thus far, a daily oral PrEP product, TDF/FTC (Truvada^®^), has been approved for use but only in the United States [14]. This US Food and Drug Administration (FDA) regulatory approval in July 2012 was quickly followed by initial World Health Organization (WHO) guidelines for the conduct of PrEP demonstration projects [15]. More recently, WHO has issued additional guidance for key populations [16].

A large number of demonstration projects are now underway in trial-naïve populations [17], complementing the post-trial access studies among participants of the original trials reporting efficacy. In addition, the Partners PrEP trial randomized participants in the former placebo arm to either TDF/FTC or tenofovir alone, since both products reduced HIV acquisition risk, and found that the lower-cost single drug also provides high protection [18]. This suite of projects and studies is providing valuable data to inform country implementation strategies.

Decision-makers, faced with the results of a plethora of studies in diverse populations and regulatory approval for oral PrEP only in the United States, have difficult choices to make to realize the promise of antiretroviral prophylaxis [19] for their countries. They need contextualized information on disease burden by population, analyses of how PrEP services might best be delivered, and assessment of the human resource and infrastructure requirements for potential delivery models. There are cost considerations, varying cost-effectiveness results, regulatory challenges and ethical concerns that must be addressed to ensure that oral PrEP is a tangible HIV prevention choice for those individuals who can most benefit from it. Complementing other papers in this supplement addressing PrEP, this paper outlines key economic, regulatory, distributive justice, and access issues that must be addressed in each context to realize the full potential of effective and acceptable PrEP implementation.

## Discussion

### Cost-effectiveness, pricing and trade-offs

#### Cost-effectiveness

Cost-effectiveness analysis is a standard method used for allocating resources in health policy. It seeks to determine how the most effective policy can be implemented at the least cost. Studies of oral PrEP cost-effectiveness have used a variety of metrics, estimating cost per HIV infection averted [20–22], cost per quality-adjusted life year (QALY) gained [21, 23–27], cost per disability-adjusted life year (DALY) averted [28], cost per year life saved [29] and PrEP years per infection averted [30]. The studies have examined PrEP for heterosexual transmission in southern Africa [20] and South Africa [21, 22, 29, 31–33] and for other modes of transmission, among PWID in Ukraine [25] and MSM in the USA [23, 24, 26, 27] and Peru [28].

A systematic review of 13 cost-effectiveness studies found that key considerations to address in assessing cost-effectiveness of PrEP are cost, epidemic context, individual adherence level, PrEP programme coverage and prioritization strategy [34]. PrEP could be a potentially cost-effective addition to HIV-prevention programmes, particularly when those at highest risk of HIV exposure are prioritized, although drug costs would limit cost-effectiveness. While PrEP could have impact in key populations such as MSM, the first priority for PWID might be expanding access to antiretroviral treatment (ART) and opioid substitution therapy. In considering trade-offs, prioritizing PrEP for young women in southern Africa who are at alarmingly high risk of HIV acquisition can be cost-effective, especially when there are costly obstacles to recruiting HIV-positive people for treatment using the same drug [35].

Cost-effectiveness studies guide resource allocation decisions by indicating where resources can be applied for greatest impact. Funding PrEP while other potentially more cost-effective HIV prevention interventions remain under-funded may have high opportunity costs, diverting resources from early ART initiation or other prevention strategies [34]. It is therefore important, as oral PrEP moves into demonstration projects and regular use in some settings, to obtain and integrate real-world costing data for all PrEP programme elements to replace earlier hypothetical costs. This will assist policy-makers in planning future resource allocations for PrEP as part of high-impact combination prevention.

#### Pricing

The price of drugs is a key component of overall programme costing. The price of tenofovir-containing ART regimens from originator sources has remained static since 2007, while the lowest prices of stand-alone tenofovir fell by almost half in 12 months, from $48 per person year in 2013 to $26 in 2014 [36]. In 2012, it was estimated that over half of all people on ART in countries with generic access were on tenofovir-based regimens, with this proportion estimated to rise to 70% of patients on first-line treatment regimens by the end of 2014 [36].

#### Trade-offs

Paying for tenofovir-based PrEP when access to ART is not universal is an issue that requires careful reflection in each context. People who do not acquire HIV because of the effective use of PrEP when they are at most risk of HIV acquisition will avoid lifelong ART and its associated costs.

Drugs that are not used for ART, such as maraviroc, which is currently being assessed in the Next-PrEP clinical trial [37], would not present direct competition for drug use, but the overall issue of resource allocation remains. The dapivirine vaginal ring, replaced monthly, is being assessed in two Phase III trials [7, 8]. Phase I trials of the long-acting injectables, bi-monthly rilpivirine (TMC 278), a non-nuclease reverse transcriptase inhibitor [38], and tri-monthly cabotegravir (S/GSK1265744), an integrase inhibitor [39], have reported safety and tolerability [9] with Phase II trials following suit [10, 11]. Thus, the potential array of delivery options could expand to provide choices for PrEP that would not necessarily compete with treatment drug demands. Issues of cost, access, equity and trade-offs will remain. However, without regulatory approval no PrEP option can be rolled out where it is most needed.

### Regulatory issues

Following FDA approval of oral TDF/FTC for a prevention indication, its manufacturer Gilead Sciences, Inc. applied for approval in 4 of the 14 countries that hosted TDF/FTC PrEP trials: South Africa (December 2013), Thailand (April 2014), and Australia and Brazil (late 2014). Figure 1 outlines the countries in which PrEP trials have taken place and the current status of regulatory review for a prevention indication of oral TDF/FTC [40].

**Figure 1:** Regulatory approval in trial host countries for daily TFD/FTC. Permission to use granted by AVAC.

It is unclear when the four pending applications will be decided and when the company might apply in the other host trial countries. The drug has been registered for HIV treatment in 154 countries worldwide, including 110 low- and middle-income countries [41]. It is available as Gilead-branded Truvada^®^ or as generic versions in developing countries through Gilead's partnerships with generic manufacturers. Gilead Sciences was the first pharmaceutical company to commit to the Medicines Patent Pool, a United Nations-backed organization established in 2010 to improve access to appropriate, affordable HIV medicines and technologies for people living with HIV in developing countries, and recently signed a licence for the new medicine tenofovir alafenamide (TAF) [42].

Thus, while TDF/FTC is already available in many countries as an approved therapeutic, the absence of a prevention indication outside the United States limits programmatic and policy decisions to expand access to PrEP. However, recent clinical trial developments may move TDF/FTC onto a faster track for regulatory approval. The PROUD trial of daily oral PrEP in England was unblinded in October 2014 on the recommendation of its Data Safety Monitoring Board when it became no longer ethical, in light of the compelling findings, to continue the delayed arm that had no access for 12 months [43]. Two weeks later, the IPERGAY trial of event-driven PrEP (2 pills before sex and 1 pill 24 h and 48 h later) in France and Canada was unblinded and its placebo arm offered PrEP [44]. PROUD found that PrEP reduced HIV risk by 86% (90% CI: 58;98) compared with no-PrEP (*p*=0.0002) [2], while Ipergay also reported an 86% reduction in HIV incidence (95% CI: 40;99), *p*=0.002 [3]. Gilead Sciences is considering submission to the European Medicines Agency (EMA) for regulatory approval, which would expand access in Europe. However, EMA regulatory approval for low- and middle-income countries can only be done for medicinal products for human use that are intended exclusively for markets outside the European Union [45].

## Ethical considerations

### Principles of distributive justice

While cost-effectiveness analysis seeks to determine how the most effective policy can be implemented at the least cost, the result may conflict with the application of ethical principles designed to introduce important values other than monetary ones. Examining leading principles of justice can identify different priorities for allocation of PrEP although the principles can conflict, requiring a balance of competing concerns. There is no uniquely correct way of doing this balancing. Furthermore, there is no consensus on what weight to give to the different principles [46]. It has even been argued that the impossibility of achieving a consensus on which principle to choose requires abandoning the search for substantive principles of justice and instead, introducing a method that involves fairness in the procedural aspects of allocation decision-making [47]. However, procedural fairness does not guarantee fairness as an outcome that would accord with any of the leading substantive principles of distributive justice.

The principle of **utility** [48, 49] is the one most widely used in health policy: choose the option that has the most beneficial consequences and the fewest harmful consequences for society as a whole. The philosophical and economic literature contains numerous versions of utilitarian theory. A general form of consequentialist utility theory, known as Total Consequentialism, indicates that “… moral rightness depends only on the *total* net good in the consequences (as opposed to the average net good per person)” [50]. Applying this principle requires specifying which consequences are to count: minimizing costs, preventing new infections or ensuring fairness in the distribution of PrEP. It is evident that the utilitarian principle can yield different results depending on an array of empirical facts and circumstances.

Two principles that are sometimes conflated are the **egalitarian** principle [49] and the principle of **equity** [51, 52]. Whereas the egalitarian principle mandates treating all in need equally, the principle of equity allows for contextual factors to be considered in a fair distribution of resources. When deciding which groups should receive PrEP first as prevention programmes are scaled up, decision-makers should consider whether all in need should be treated equally or whether certain groups, such as those who are marginalized, stigmatized or typically underserved, should be given preference. A well-known application of the **egalitarian** principle is a lottery among the pool of potential users when supplies are limited. Egalitarian principles make no distinctions regarding who might benefit most from an intervention or what choice would best serve the goals of public health. The principle of **equity** has a specific meaning in the context of access to health care: “The dominant conceptualization of equitable access to health care among health service researchers builds on the idea that the utilization of services should reflect actual needs for care” [53]. Application of this principle to PrEP might focus on traditionally underserved populations, as well as on young women at high risk of infection because poverty, misogyny and their limited social capital make them less able than others to avoid unprotected sex.

The **prioritarian** principle [54] calls for ensuring that resources are provided to the least-advantaged members or groups in society. In the context of HIV prevention, these might be those at greatest risk of becoming infected, the poorest people, the most vulnerable or the most highly stigmatized.

It is clear that applying principles of distributive justice for making health care allocations cannot guarantee justice in outcomes. They are designed for selecting populations to be given priority in a particular context, with the principle of equity designed to eliminate socio-economic and other barriers to care.

### Allocating resources for HIV prevention

Because resources for PrEP are insufficient to meet the needs of all who could benefit, decisions are needed about which populations should be given priority for receiving PrEP. People who engage in behaviour that places them at higher risk of HIV acquisition and whose sexual networks likely extend beyond their own subpopulation are an obvious choice because they have the greatest likelihood of transmitting HIV if they acquire it. Providing them with access to antiretroviral prevention first may mean that HIV infection will spread more slowly in a country. Thus, a logical choice as early priority populations for receiving PrEP could be young women, sex workers, MSM and PWID; however, these may be among the hardest people to reach.

The **prioritarian** principle operates as a constraint on the utilitarian principle to ensure that the most disadvantaged individuals are not ignored in the effort to scale up HIV prevention [46]. Different criteria exist for determining who are the most disadvantaged. Populations already identified as early priorities for HIV prevention according to the utilitarian principle appear also to be among the least advantaged according to several criteria. In most societies, they are stigmatized, marginalized, and typically engaged in illegal behaviour, and are often at greater risk from authorities. This includes PWID in countries with punitive drug policies, MSM in some African countries where homosexual behaviour is a criminal offense and sex workers in most countries worldwide. Thus, the utilitarian and prioritarian principles concur that those who are most at risk of HIV exposure should be the first to receive PrEP.

The fastest growing group of newly infected people in sub-Saharan Africa is young women. According to UNAIDS, “In sub-Saharan Africa, women and girls account for almost 57% of adults living with HIV. Recent surveys reveal that in South Africa, Zambia, and Zimbabwe, young women (age 15–24) are five to six times more likely to be infected than young men of the same age” [55]. The principle of **equity** might, therefore, call for scaling up HIV testing and offering PrEP to young HIV-negative women who are likely to be at risk.

Finally, HIV serodiscordant couples might be prioritized for PrEP, when the HIV-positive partner, whether on ART or not, is not virally supressed. A 96% reduction in HIV-negative partners’ risk of HIV acquisition was recently reported in a study of HIV-serodiscordant couples offered immediate ART for the HIV-positive partner and PrEP for the HIV-negative partner [56]. According to the principle of **urgent need** [57], HIV-negative partners in serodiscordant couples where the HIV-positive partner is not virally suppressed would have priority for PrEP because they risk acquiring HIV with each sexual act.

The urgent need principle can be combined with the utilitarian principle in setting priorities for allocating PrEP, with the principle of equity giving priority to stigmatized and marginalized populations, such as MSM, sex workers and PWID, and young women and serodiscordant couples.

## Advocacy for access

Since the early PrEP trial controversies [58, 59], advocates have played an active role in monitoring trials, interpreting trial results, advocating for access, disseminating information and, in the United States, engaging in the regulatory process.

Shortly after several of the first oral PrEP trials were stopped in 2005 amidst controversy, AVAC and UNAIDS began a consultative process with civil society representatives, researchers and funders to develop approaches to guide productive engagement in research. The resulting Good Participatory Practice Guidelines [60] are now increasingly used across a range of HIV prevention trials and have been adapted for use in non-HIV research efforts [61].

Following the iPrEx trial results in 2010 [1], a number of advocacy groups played leading roles in their countries and communities to explain clinical trial results and push for evidence-based policies and programmes. A coalition of 14 US HIV and health advocacy organizations submitted extensive public comments to the FDA in 2012 to support approval of TDF/FTC as PrEP. Their written comments, as well as formal presentations at the public FDA Advisory Committee meeting, pointed to the compelling evidence on the efficacy of PrEP and highlighted the unique potential of this intervention [62].

Advocacy groups, representing diverse populations who need and could most benefit from PrEP, especially in countries where the PrEP trials took place, also called for an ambitious, well-coordinated scale-up of demonstration projects across diverse populations of men, women and transgender people at risk for HIV through sex [63, 64]. For example, the US Women and PrEP Working Group, a coalition of women from leading AIDS and women's health organizations, advocates for a national agenda to answer questions about the best way to make PrEP available to women as a prevention option.

Finally, web-based community efforts include AVAC's PrEP Watch website (www.prepwatch.org), a clearinghouse for information on PrEP, and the AIDS Foundation of Chicago's My PrEP Experience (www.myprepexperience.blogspot.com), featuring stories from people who have chosen to use PrEP.

## Conclusions

Decision-makers considering the introduction of PrEP in their countries are faced with competing priorities and the need to address key economic, regulatory, distributive justice and access issues. Unless these processes are informed by inputs relative to their own specific context, it will be difficult to realize the full potential of effective and acceptable PrEP implementation. Using disease burden, costing information and known effectiveness, cost-effectiveness studies that illustrate the utilitarian principle at work can provide an initial indication of the potential impact of PrEP programmes. The results may conflict with the application of egalitarian or prioritarian principles of distributive justice.

The price of PrEP varies widely and the US FDA is the only regulatory agency to approve it for HIV prevention thus far. This approval has helped pave the way for greater access and insurance reimbursement for PrEP in the United States, but even there access challenges remain for some seeking PrEP. Although the regulatory pathway in Europe seems clearer in light of the recent European trial results, answers will be needed to the question of who will pay. Countries that lack regulatory capacity to independently evaluate the use of TDF/FTC as PrEP might use WHO pre-qualification and guidance, when available. The question of who will pay for PrEP in low- and middle-income countries requires frank discussions at national level and with international donors supporting strategies to end AIDS. These will need to be underpinned by discussions of equity and ethical allocation under conditions of limited resources.

Transitioning from clinical trial efficacy to public health impact is never easy. Experiences with preventing vertical transmission, programming the female condom, providing post-exposure prophylaxis (PEP), delivering sterile needles and scaling up voluntary medical male circumcision, amongst others, provide cogent examples in HIV prevention of slow policy and programme responses, unrealized expectations and resultant limited impact. There are important lessons to be learned about the factors that facilitate and impede uptake of new HIV prevention innovations [65].

PrEP is not meant for everyone, all of the time. If done well, though, initial PrEP introduction activities will enable policy-makers and programme planners to answer the questions of who can benefit most from PrEP, how to provide it safely and efficiently, how to integrate PrEP into combination treatment and prevention programmes, and what kind of health system support is needed to ensure successful implementation.

The extraordinary feat of proving PrEP's efficacy may turn out to have been easier than ensuring that it is used well. This is not unique to PrEP and insights can be gleaned from experiences with implementation of other novel strategies. Ensuring that PrEP fulfils its potential as part of high-impact combination HIV prevention requires establishing the additional evidence, education, support services and resources that are needed, as well as the regulatory framework and cost scenarios for access to PrEP.

## References

[1] Grant RM, Lama JR, Anderson PL, McMahan V, Liu AY, Vargas L (2010). Preexposure chemoprophylaxis for HIV prevention in men who have sex with men. N Engl J Med.

[2] Medical Research Council U The PROUD study: examining the impact on gay men of using Pre-Exposure Prophylaxis (PrEP). Latest results [Internet].

[3] Molina J-M, Capitant C, Charreau I, Meyer L, Spire B, Pialoux G (2015). On demand PrEP with oral TDF-FTC in MSM: results of the ANRS ipergay trial.

[4] Baeten JM, Donnell D, Ndase P, Mugo NR, Campbell JD, Wangisi J (2012). Antiretroviral prophylaxis for HIV prevention in heterosexual men and women. N Engl J Med.

[5] Thigpen MC, Kebaabetswe PM, Paxton LA, Smith DK, Rose CE, Segolodi TM (2012). Antiretroviral preexposure prophylaxis for heterosexual HIV transmission in Botswana. N Engl J Med.

[6] Choopanya K, Martin M, Suntharasamai P, Sangkum U, Mock PA, Leethochawalit M (2013). Antiretroviral prophylaxis for HIV infection in injecting drug users in Bangkok, Thailand (the Bangkok Tenofovir Study): a randomised, double-blind, placebo-controlled phase 3 trial. Lancet.

[7] International Partnership for Microbicides The Ring Study [Internet].

[8] Microbicide Trials Network ASPIRE [Internet].

[9] Spreen W, Williams P, Margolis D, Ford SL, Crauwels H, Lou Y (2014). Pharmacokinetics, safety, and tolerability with repeat doses of GSK1265744 and rilpivirine (TMC278) long-acting nanosuspensions in healthy adults. J Acquir Immune Defic Syndr 1999.

[10] HIV Prevention Trials Network HPTN 076: phase II safety and acceptability of an investigational injectable product, TMC278 LA, for Pre Exposure Prophylaxis (PrEP) [Internet].

[11] HIV Prevention Trials Network HPTN 077 A Phase IIa safety, tolerability and acceptability study of an investigational injectable HIV integrase inhibitor, GSK1265744, for PrEP in HIV uninfected men and women [Internet].

[12] Hankins CA, de Zalduondo BO (2010). Combination prevention: a deeper understanding of effective HIV prevention. AIDS.

[13] Shattock RJ, Warren M, McCormack S, Hankins CA (2011). AIDS. Turning the tide against HIV. Science.

[14] FDA (2012). FDA, Press release: FDA approves first drug for reducing the risk of sexually acquired HIV infection [Internet].

[15] WHO (2012). Guidance on oral pre-exposure prophylaxis (PrEP) for serodiscordant couples, men and transgender women who have sex with men at high risk of HIV. Recommendations for use in the context of demonstration projects. [Internet].

[16] World Health Organization (2014). Consolidated guidelines on HIV prevention, diagnosis, treatment and care for key populations [Internet].

[17] AVAC (2015). Ongoing and planned PrEP evaluation studies [Internet].

[18] Baeten JM, Donnell D, Mugo NR, Ndase P, Thomas KK, Campbell JD (2014). Single-agent tenofovir versus combination emtricitabine plus tenofovir for pre-exposure prophylaxis for HIV-1 acquisition: an update of data from a randomised, double-blind, phase 3 trial. Lancet Infect Dis.

[19] Hankins CA, Dybul MR (2013). The promise of pre-exposure prophylaxis with antiretroviral drugs to prevent HIV transmission: a review. Curr Opin HIV AIDS.

[20] Abbas UL, Anderson RM, Mellors JW (2007). Potential impact of antiretroviral chemoprophylaxis on HIV-1 transmission in resource-limited settings. PLoS One.

[21] Hallett TB, Baeten JM, Heffron R, Barnabas R, de Bruyn G, Cremin Í (2011). Optimal uses of antiretrovirals for prevention in HIV-1 serodiscordant heterosexual couples in South Africa: a modelling study. PLoS Med.

[22] Pretorius C, Stover J, Bollinger L, Bacaër N, Williams B (2010). Evaluating the cost-effectiveness of pre-exposure prophylaxis (PrEP) and its impact on HIV-1 transmission in South Africa. PLoS One.

[23] Paltiel AD, Freedberg KA, Scott CA, Schackman BR, Losina E, Wang B (2009). HIV preexposure prophylaxis in the United States: impact on lifetime infection risk, clinical outcomes, and cost-effectiveness. Clin Infect Dis.

[24] Desai K, Sansom SL, Ackers ML, Stewart SR, Hall HI, Hu DJ (2008). Modeling the impact of HIV chemoprophylaxis strategies among men who have sex with men in the United States: HIV infections prevented and cost-effectiveness. AIDS.

[25] Alistar S Effectiveness and cost-effectiveness of oral pre-exposure prophylaxis for injection drug users in mixed HIV epidemics.

[26] Koppenhaver RT, Sorensen SW, Farnham PG, Sansom SL (2011). The cost-effectiveness of pre-exposure prophylaxis in men who have sex with men in the United States: an epidemic model. J Acquir Immune Defic Syndr.

[27] Juusola JL, Brandeau ML, Owens DK, Bendavid E (2012). The cost-effectiveness of preexposure prophylaxis for HIV prevention in the United States in men who have sex with men. Ann Intern Med.

[28] Gomez GB, Borquez A, Caceres CF, Segura ER, Grant RM, Garnett GP (2012). The potential impact of pre-exposure prophylaxis for HIV prevention among men who have sex with men and transwomen in Lima, Peru: a mathematical modelling study. PLoS Med.

[29] Walensky RP, Park J-E, Wood R, Freedberg KA, Scott CA, Bekker L-G (2012). The cost-effectiveness of pre-exposure prophylaxis for HIV infection in South African women. Clin Infect Dis.

[30] Vissers DCJ, Voeten HACM, Nagelkerke NJD, Habbema JDF, de Vlas SJ (2008). The impact of pre-exposure prophylaxis (PrEP) on HIV epidemics in Africa and India: a simulation study. PloS One.

[31] Williams BG, Abdool Karim SS, Karim QA, Gouws E (2011). Epidemiological impact of tenofovir gel on the HIV epidemic in South Africa. J Acquir Immune Defic Syndr.

[32] Alistar SS, Grant PM, Bendavid E (2014). Comparative effectiveness and cost-effectiveness of antiretroviral therapy and pre-exposure prophylaxis for HIV prevention in South Africa. BMC Med.

[33] Cremin I, Alsallaq R, Dybul M, Piot P, Garnett G, Hallett TB (2013). The new role of antiretrovirals in combination HIV prevention: a mathematical modelling analysis. AIDS.

[34] Gomez GB, Borquez A, Case KK, Wheelock A, Vassall A, Hankins C (2013). The cost and impact of scaling up pre-exposure prophylaxis for HIV prevention: a systematic review of cost-effectiveness modelling studies. PLoS Med.

[35] Over M (2014). Is PrEP cost-effective? [Internet]. Global Health Policy Blog.

[36] Medecins sans frontieres AC Untangling the Web of antiretroviral price reductions: 17th Edition – July 2014 [Internet]. 2014 Jul [cited 2014 Nov 21]; p. 26. Report No.: 17.

[37] HIV Prevention Trials Network HPTN 069: a phase II randomized, double-blind, study of the safety and tolerability of Maraviroc (MVC), Maraviroc+Emtricitabine (MVC+FTC), Maraviroc+Tenofovir disoproxil fumarate (MVC+TDF), or Tenofovir disoproxil fumarate+Emtricitabine (TDF+FTC) for Pre-Exposure Prophylaxis (PrEP) to prevent HIV transmission in at-risk men who have sex with men and in at-risk women [Internet].

[38] Jackson A, Else L, Tjia J, Seymour N, Stafford M, Back D Rilpivirine-LA formulation: pharmacokinetics in plasma, genital tract in HIV-females and rectum in males.

[39] Spreen W, Min S, Ford SL, Chen S, Lou Y, Bomar M (2013). Pharmacokinetics, safety, and monotherapy antiviral activity of GSK1265744, an HIV integrase strand transfer inhibitor. HIV Clin Trials.

[40] AVAC Pre-exposure prophylaxis (PrEP) by the numbers: efficacy, regulatory approval and more [Internet].

[41] Truvada Registration in the Developing World [Internet]. http://www.gilead.com/~/media/Files/pdfs/other/TruvadaRegistrationDocment%20030315.pdf.

[42] Medicines Patent Pool The Medicines Patent Pool broadens collaboration with Gilead Sciences: signs licence for Phase III medicine Tenofovir Alafenamide (TAF) [Internet].

[43] MRC Clinical Trials PHE PROUD study interim analysis finds pre-exposure prophylaxis (PrEP) is highly protective against HIV for gay men and other men who have sex with men in the UK [Internet].

[44] Collins S IPERGAY PrEP study shows early efficacy in protecting gay men from HIV: all participants to switch to active drug [Internet].

[45] (2009). EMEA procedural advice on medicinal products intended exclusively for markets outside the community under article 58 of regulation (ec) no 726/2004 in the context of co-operation with the World Health Organization (WHO) [Internet]. http://www.ema.europa.eu/docs/en_GB/document_library/Regulatory_and_procedural_guideline/2010/02/WC500074039.pdf.

[46] Macklin R, Cowan E (2012). Given financial constraints, it would be unethical to divert antiretroviral drugs from treatment to prevention. Health Aff Proj Hope.

[47] Daniels N (2000). Accountability for reasonableness: establishing a fair process for priority setting is easier than agreeing on principles. BMJ.

[48] Smart J, Edwards P (1967). Utilitarianism. The encyclopedia of philosophy.

[49] Beauchamp T, Childress J (2001). Principles of biomedical ethics.

[50] Consequentialism (2014). The Stanford encyclopedia of philosophy [Internet].

[51] Daniels N Just health care.

[52] Daniels N (2008). Just health: meeting health needs fairly.

[53] Justice and Access to Health Care Stanford encyclopedia of philosophy [Internet].

[54] Brock D, Rhodes R, Battin M, Silvers A (2002). Priority to the worse off in health-care resource prioritization. Medicine and social justice.

[55] UNAIDS The global coalition on women and AIDS [Internet].

[56] Baeten JM (2015). Near elimination of HIV transmission in a demonstration project of PrEP and ART.

[57] Brock D (2003). Separate spheres and indirect benefits, cost effectiveness and resource allocation. Cost Eff Resour Alloc.

[58] Singh JA, Mills EJ (2005). The Abandoned trials of pre-exposure prophylaxis for HIV: what went wrong?. PLoS Med.

[59] Mills E, Rachlis B, Wu P, Wong E, Wilson K, Singh S (2005). Media reporting of tenofovir trials in Cambodia and Cameroon. BMC Int Health Hum Rights.

[60] UNAIDS/AVAC (2011). Good participatory practice guidelines for biomedical hiv prevention trials 2011 [Internet]. Joint United Nations Programme on HIV/AIDS.

[61] Critical Path to TB Drug Regimens (2012). Good participatory practice guidelines for TB drug trials [Internet].

[62] (2012). Leading AIDS advocates support FDA approval of Pre-Exposure Prophylaxis (PrEP), a promising new HIV prevention method, for men and women. http://www.avac.org/press-release/leading-aids-advocates-support-fda-approval-pre-exposure-prophylaxis-prep-promising.

[63] AVAC (2011). AVAC report 2011: the end? [Internet].

[64] Project Inform PrEP roadmap to the real world: establishing the real-world effectiveness of PrEP through demonstration projects [Internet].

[65] Dickson KE, Tran NT, Samuelson JL, Njeuhmeli E, Cherutich P, Dick B (2011). Voluntary medical male circumcision: a framework analysis of policy and program implementation in eastern and southern Africa. PLoS Med.
